# The global burden of childhood diarrhea and its epidemiological characteristics from 1990 to 2021

**DOI:** 10.3389/fped.2025.1656234

**Published:** 2025-08-28

**Authors:** Hai-Yan Zhu, Fan Xu, Wen-Zhuo Zhao, Hai-Xiao Wang, Hong-Gang Wang

**Affiliations:** ^1^ Department of Pediatrics, The Affiliated Huaian No.1 People’s Hospital of Nanjing Medical University, Huai'an, Jiangsu, China; ^2^ Department of Gastroenterology, The Affiliated Huaian No.1 People’s Hospital of Nanjing Medical University, Huai'an, Jiangsu, China; ^3^ Department of General Surgery, The Affiliated Huaian No.1 People’s Hospital of Nanjing Medical University, Huai'an, Jiangsu, China

**Keywords:** diarrhea diseases, global burden of disease, disability-adjusted life years, incidence, prevalence

## Abstract

**Introduction:**

Diarrhea diseases remains a major contributor to global mortality and morbidity in children. This study aims to provide an updated assessment of rates in diarrhoea prevalence, incidence, mortality, and disability-adjusted life-years (DALYs) from 1990–2021, specifically focusing on including prevalence investigation alongside other measures. The analysis is stratified by sex, age, and socio-demographic index (SDI) at global, regional, and national levels.

**Methods:**

Data for this study was obtained from the 2021 Global Burden of Diseases, Injuries, and Risk Factors Study (GBD). Children aged 0–14 years with diarrhea were included in the analysis. The analyses were disaggregated by gender, 3 age categories, 21 GBD regions, 204 nations/territories, and 5 SDI quintiles. Incidence, all-cause mortality, cause-specific mortality, disability-adjusted life years (DALYs), and the corresponding estimated annual percent changes (EAPCs) were investigated.

**Results:**

In 2021, the global burden of diarrhea diseases remained substantial, with a total of 2,432,874,591 cases with an ASPR of 1,252.09 cases per 100,000 individuals (95% UI: 1,032.41–1,474.93). The ASIR was 83,866.84 per 100,000 people (95% UI: 66,140.64–101,854.13), while the ASDR was 18.6 per 100,000 persons (95% UI: 13.99–24.79). Additionally, the age-standardized DALY rate was 1,784.28 per 100,000 individuals (95%UI: 1,361.38–2,320.22). Regionally, areas with high-middle SDI exhibited the greatest ASPR, ASIR, ASDR, and age-standardized DALY rates, whereas high SDI regions had the lowest rates. The ASPR is mostly concentrated in children aged 10–14 years old, among which the ASPR of children under 5 years old has the most significant decline, from 3,138.81 per 100,000 people (95% UI: 2,749.19–3,557.51) in 1,990–885.07 per 100,000 people (95% UI: 755.93–1,029.39), a decrease of 71%. Geospatially, South Asia had the highest ASPR and the highest ASIR. The most pronounced increase was noted in the high-income Asia-Pacific region, which is the only area exhibiting growth, with an EAPC of 1.46 (95% CI: 1.11–1.82). Interestingly, between 1990 and 2021, the ASDR in Western Europe displayed the most pronounced rise, with an EAPC of 0.81, whereas the steepest decline was observed in East Asia. The greatest ASDR and age-standardized DALY rates were observed in the western region of sub-Saharan Africa, West Africa and Central Africa. Among countries, Madagascar had both the highest ASIR and ASDR. Furthermore, African countries exhibited the highest age-standardized DALY rate. Globally, unsafe water sources remained the primary risk factor for childhood diarrhea mortality and disability-adjusted life years (DALYs) from 1990–2021. While predominant in all Sociodemographic Index (SDI) regions except high-SDI areas, high-SDI regions reported non-exclusive breastfeeding and childhood wasting as leading mortality risk factors. Unsafe water sources are expected to persist as the principal contributor in the future.

**Discussion:**

The burden of childhood diarrhea diseases globally has been decreasing but remains a substantial contributor to DALYs. Low-middle SDI regions show persistently high age-standardized rates, with South Asia bearing the highest burden. Our study clarifies the global and regional epidemiology of childhood diarrheal diseases and identifies unsafe water sources as the predominant risk factor. These findings highlight the need for region-specific water safety initiatives in high-burden areas and are vital for shaping public health strategies and policy decisions for prevention.

## Introduction

Diarrhea disease remains a pressing public health concern globally, ranking as the third most fatal infectious disease among children under the age of five, following lower respiratory tract infections and neonatal preterm complications. Childhood diarrhea stems from interconnected environmental, host, and socioeconomic determinants where contaminated water and inadequate sanitation perpetuate fecal-oral transmission while nutritional deficiencies including nonexclusive breastfeeding, zinc deficiency, and malnutrition-compromise intestinal immunity. Young children face heightened vulnerability owing to immune immaturity, suboptimal vaccine coverage, and comorbidities, risks further amplified by poverty-driven conditions like overcrowding, limited healthcare access, and environmental disruptions. Despite a reduction in child mortality linked to diarrhea since the 1990s (1), the ailment persists as a substantial health challenge. Its mortality and morbidity rates weigh most heavily on low- and middle-income countries, where the impact is particularly severe. Furthermore, significant disparities in prevalence exist within national borders. Diarrhea during early childhood, especially within the first two years of life, impairs nutrient absorption and hinders healthy physical growth (2, 3). Given this substantial burden, gaining insights into the prevalence, incidence, and mortality associated with diarrhea diseases is essential for informing policy decisions, optimizing resource allocation, and designing effective interventions.

The Global Burden of Disease (GBD) investigation (4) reported a 47.0% (39.9–52.9) reduction in global diarrhea disease rates between 2010 and 2021. Although many countries have achieved notable reductions in mortality from diarrhea among children under five, the decrease in incidence has been comparatively modest. Diarrhea remains the fourth most significant contributor to disability-adjusted life years (DALYs) among children in this age group (5, 6). Despite the disruptions brought about by the COVID-19 pandemic, diarrhea diseases persisted as a prominent health issue, ranking within the top 25 Level 3 causes of DALYs in 2021 (4). Furthermore, diarrheal diseases have consistently ranked as the third leading cause of DALYs among children under ten years old over the nearly three decades since 1990 (6). To our current understanding, no comprehensive long-term global trend report exists on the epidemiology of diarrhea diseases in children under 14 years old. This investigation employs the GBD database to examine trends and patterns in morbidity, mortality, and DALYs associated with diarrhea diseases among children under 14 between 1990 and 2021, alongside the associated risk factors. By offering insights into the GBD 2021 estimates, this analysis aims to encourage the creation of novel preventive and therapeutic approaches, ultimately mitigating the health risks posed by childhood diarrhea diseases.

## Methods

### Data acquisition

The data employed in this investigation originates from the GBD 2021. This iteration of the GBD investigation provides a comprehensive assessment of health losses attributed to 371 diseases, injuries, and disorders, along with 88 risk factors, spanning 204 countries and regions, employing up-to-date epidemiological data and standardized methodologies (4, 7). Advanced statistical techniques are employed within the GBD database to address data gaps and mitigate the impact of confounding variables. The design and methodological framework of the GBD investigation has been comprehensively detailed in prior GBD publications (4). To facilitate analysis of the age distribution of the burden of childhood diarrhea diseases, data were categorized into the following age groups: under 5 years, 5–9 years, and 10–14 years.

### Estimation framework

The GBD investigation applies sophisticated modeling approaches to assess the impact of childhood diarrhea diseases. The DisMod-MR 2.1 (Disease Model—Bayesian Meta-Regression) tool is employed to determine both occurrence and prevalence. This Bayesian geospatial software synthesizes multiple disease parameters, epidemiological correlations, and geospatial data to produce reliable estimates. The GBD study adopts advanced statistical models to quantify the impact of childhood diarrhea diseases, with DisMod-MR 2.1 serving as a key tool for calculating incidence and prevalence. By integrating various disease metrics, epidemiological associations, and geospatial data, this software delivers precise and comprehensive estimations (4).

Mortality estimates are generated using the Cause of Death Ensemble model (CODEm) system. This approach integrates vital registration and autopsy data, encompassing records containing non-specific codes. Data undergo thorough refinement prior to analysis to enhance precision. CODEm employs multiple models to achieve greater precision in mortality estimation. These models are employed in the 2021 database, facilitating a thorough assessment of the burden posed by childhood diarrhea diseases. Methodological variations among data sources are accounted for to ensure consistency and reliability in estimating the incidence, prevalence, and mortality associated with these diseases. The calculation of DALYs related to childhood diarrhea diseases incorporates two elements: years lived with disability, which captures the burden of living with the condition, and years of life lost, reflecting the effect of premature death. Detailed descriptions of these methods are depicted in the Supplementary Methods section.

### Socio-demographic index (SDI)

The SDI employs fertility rates, educational attainment, and per capita income to quantify the social development level of countries or regions (8–10). This index is measured on a scale from 0–1, with higher values reflecting more advanced socio-economic development (10). It is well-established that SDI correlates with disease incidence and mortality. In this investigation, countries and regions were classified into five SDI categories—low, low-middle, middle, middle-high, and high—to explore the association between the burden of childhood diarrhea diseases and levels of social development.

### Risk factors

In addition to fundamental indicators such as incidence, prevalence, mortality, and DALYs, this investigation explores the influence of particular risk factors on the burden of childhood diarrhea diseases (7, 11–13). Particular attention is given to multiple critical risk factors highlighted in the GBD 2021 investigation: ambient particulate matter pollution, child stunting, child underweight, child wasting, discontinued breastfeeding, household air pollution from solid fuels, low birth weight for gestational age, lack of access to handwashing facilities, non-exclusive breastfeeding, short gestation for birth weight, unsafe sanitation, unsafe water sources, vitamin A deficiency, and zinc deficiency. The analysis includes DALYs and mortality data associated with these factors, stratified by SDI regions to highlight the geographical variations in their impacts.

### Statistical analysis

The patterns in age-standardized rates (ASR) of incidence, prevalence, mortality, and DALYs for childhood diarrhea diseases were evaluated using the estimated annual percentage change (EAPC) (14). The following formula was employed to calculate ASR per 100,000 individuals:

$$\mathrm{ASR}=\frac{\sum_{i=1}^{A}a_{i}\;w_{i}}{\sum_{i=1}^{A}w_{i}}\times 100,000$$

(*a_i_*: age-specific rate for the i-th age group; is the number of people in the standard population relative to the ith age group; number of age groups). The estimation of EAPCs relies on a regression model that captures the pattern of ASRs across a defined period (15). The model is denoted as Y = α + βX + e, where Y denotes the natural logarithm of ASR, X corresponds to the calendar year, α represents the intercept term, β indicates the slope or trend, and e denotes the error term. The formula for calculating EAPC is 100 × [exp(β) − 1], denoting the annual percentage change. A linear regression model is employed to determine the 95% confidence interval (CI). If both the EAPC value and the lower limit of its 95% CI are positive, the ASR is viewed as declining. Conversely, if both the EAPC and the upper limit of its 95% CI are negative, the ASR is regarded as decreasing. In cases where neither condition is met, the ASR is deemed stable (14). The association between SDI and the ASR of childhood diarrhea diseases is evaluated using Spearman's rank correlation. All analyses and visualizations were conducted with the World Health Organization's Health Equity Assessment Toolkit and R statistical software version 4.2.2.

## Results

### Global level

**Figure 1:**
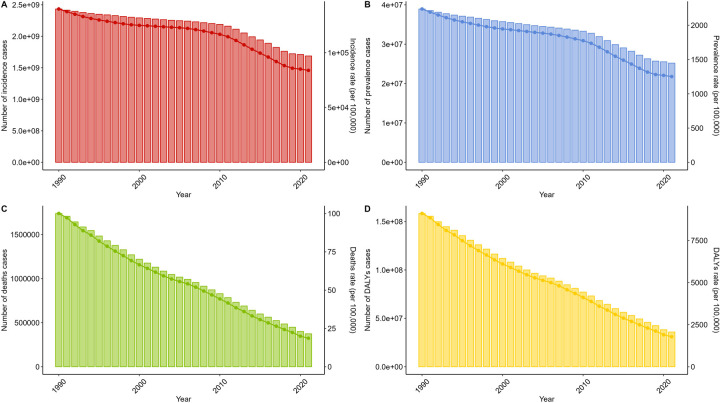
Trends in diarrhea diseases incidence, prevalence, deathes and disability-adjusted life-years from 1990 to 2021. **(A)** Trends in incident cases and incidence rate. **(B)** Trends in prevalence cases and prevalence rate. **(C)** Trends in death cases and death rate. **(D)** Trends in DALYs cases and DALYs rate.

In 2021, the global number of childhood diarrhea disease cases was estimated to be 2,432,874,591 [95% uncertainty interval (UI): 1,972,567,638–2,885,056,581], reflecting a reduction of approximately 31% relative to 1990. The age-standardized incidence rate (ASIR) also displayed a decline, decreasing from 139,888.61 per 100,000 (95% UI: 113,421.28–165,888.77) in 1990 to 83,866.84 per 100,000 (95% UI: 66,140.64–101,854.13) by 2021, marking a 41% decrease. The EAPC was recorded at −1.48 (95% CI: −1.69 to −1.28), confirming a consistent downward trend in the incidence of childhood diarrhea diseases (Figure 1A, Table 1).

**Table 1:** Incidence of diarrhea disease in children from 1990–2021 at the global, sex, age-group, and SDI regional levels.

Dimension	Rate per 100,000 (95% UI)				
	1990		2021		1990–2021
	Incidence cases	Incidence rate	Incidence cases	Incidence rate	EAPC
Global	2,432,874,591 (1,972,567,638–2,885,056,581)	139,888.61 (113,421.28–165,888.77)	1,687,284,354 (1,330,657,927–2,049,163,800)	83,866.84 (66,140.64–101,854.13)	−1.48(−1.69–1.28)
Sex					
Female	1,211,577,082 (984,362,446–1,421,170,167)	143,270.08 (116,401.74–168,054.65)	841,792,039 (66,98,89,221–1,010,314,344)	86,453.3 (68,798.63–103,760.8)	−1.47(−1.65–1.29)
Male	1,221,297,509 (986,772,738–1,462,183,662)	136,688.17 (110,440.05–163,648.26)	845,492,315 (660,768,705–1,036,678,153)	81,440.99 (63,647.72–99,856.73)	−1.49(−1.72–1.26)
Age					
<5 years	1,178,110,288 (1,000,354,993–1,366,541,108)	190,036.82 (161,363.74–220,431.93)	392,778,890 (324,124,687–463,633,235)	59,677.27 (49,246.22–70,442.6)	NA
5–9 years	730,238,621 (513,922,230–955,785,782)	125,141.42 (88,071.15–163,793.57)	58,58,84,337 (43,69,30,941–73,94,92,315)	85,274.91 (63,594.89–107,632.41)	NA
10–14 years	524,525,682 (357,214,824–703,791,388)	97,917.25 (66,684.04–131,382.16)	708,621,127 (531,723,162–912,639,477)	106,298.07 (79,762.15–136,902.23)	NA
SDI region					
High middle	167,766,090 (128,528,676–208,648,896)	61,312.84 (46,972.89–76,254.12)	66,211,292 (47,62,0,214–86,82,6,573)	28,676.51 (20,624.6–37,605.11)	−2.08(−2.38–1.78)
High	75,253,081 (52,673,613–101,077,757)	40,500.5 (28,348.44–54,399.1)	52,775,793 (37,30,2961–70,76,8129)	30588.19 (21,620.33–41,016.32)	−0.26(−0.67–0.15)
Low middle	955,639,990 (775,463,192–11,29,481302)	202,418 (164,254.02–239,240.04)	641,281,302 (505,232,840–786,770,217)	110,596.01 (87,132.96–135,687.17)	−1.88(−2.02–1.74)
Low	507,688,949 (424,458,090–590,965,715)	221,782.05 (185,422.96–258,161.2)	577,336,992 (471,311,290–686,562,080)	125,446.53 (102,408.76–149,179.47)	−1.66(−1.9–1.42)
Middle	725,250,106 (575,135,158–871,743,550)	125,646.68 (99,639.86–151,026.08)	348,701,324 (268,772,284–432,709,482)	61,514.44 (47,414.15–76,334.33)	−2.32(−2.43–2.21)

EAPC, estimated annual percentage change; SDI, sociodemographic index; UI, uncertainty interval. *EAPC is expressed as 95% CI.

In 2021, the global burden of childhood diarrhea diseases remained considerable, totaling 25,190,229 cases (95% UI: 20,770,723–29,673,552), representing a 35.3% decrease compared to 1990. Similarly, the age-standardized prevalence rate (ASPR) declined from 2,238.74 per 100,000 (95% UI: 1,865.21–2,629.59) in 1990 to 1,252.09 per 100,000 (95% UI: 1,032.41–1,474.93) in 2021. The EAPC for ASPR was −1.72 (95% CI: −1.92 to −1.52) (Figure 1B, Supplementary Table S1).

In 2021, deaths attributable to childhood diarrhea diseases were estimated at 374,246 (95% UI: 281,541–498,684), with an age-standardized death rate (ASDR) of 18.6 per 100,000 individuals (95% UI: 13.99–24.79). The estimated EAPC for ASDR stood at −5% (95% CI: −5.3 to −4.69) (Figure 1C, Supplementary Table S2).

The number of DALYs associated with childhood diarrhea diseases in 2021 was 35,897,190 cases (95% UI: 27,389,119–46,679,614). The age-standardized DALY rate was 1,784.28 per 100,000 individuals (95% UI: 1,361.38–2,320.22), with the EAPC for DALYs estimated at −4.85 (95% CI: −5.15 to −4.56) (Figure 1D, Supplementary Table S3).

### Age and gender level

**Figure 2:**
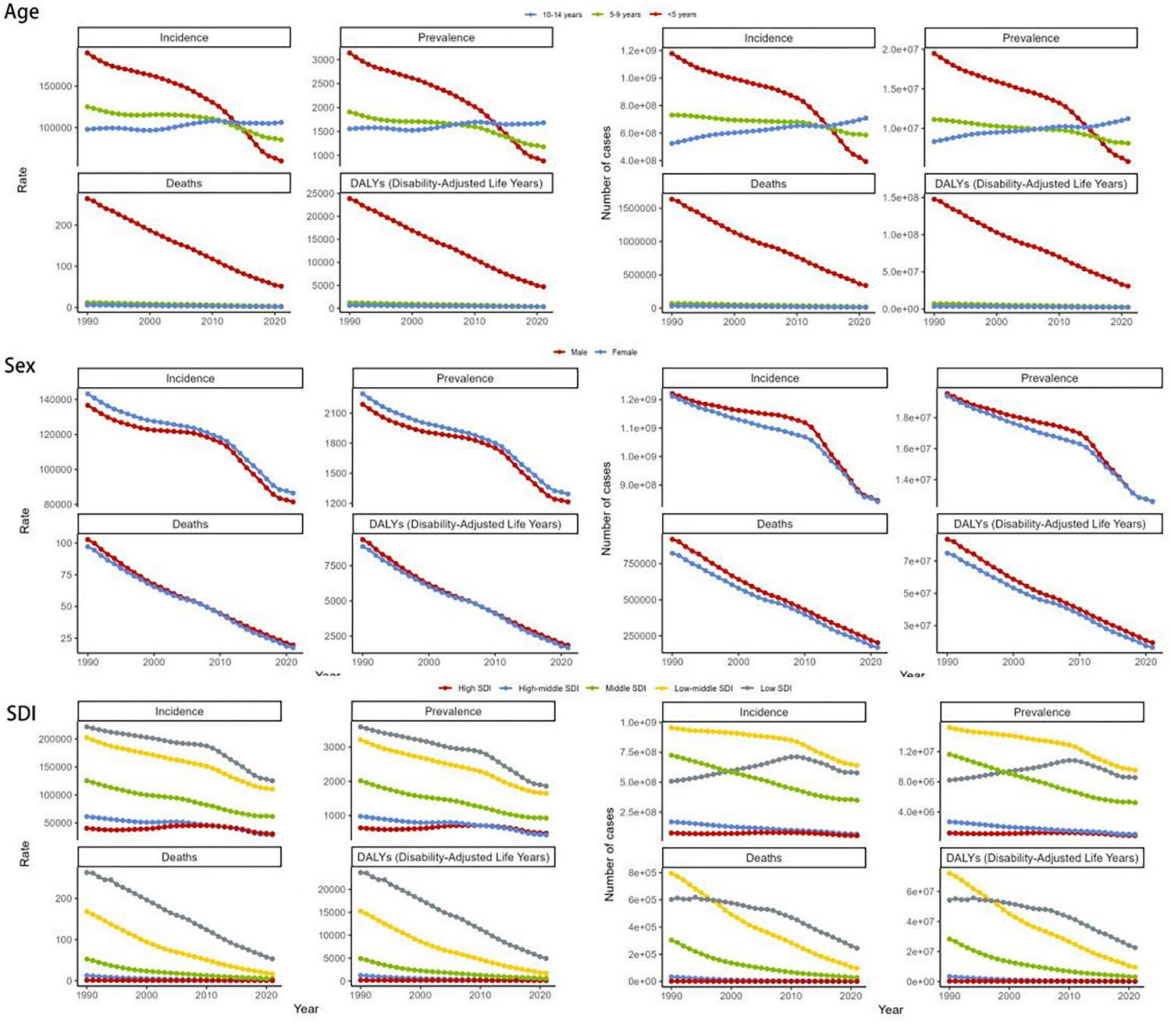
The global disease burden of diarrhea diseases for age, gender and SDI in 204 countries and territories. SDI, socio-demographic index.

In 2021, the ASPR of childhood diarrhea diseases was predominantly concentrated among children aged 10–14 years. Among them, the ASPR of children under 5 years old decreased the most significantly, from 3,138.81 per 100,000 people (95% UI: 2,749.19–3,557.51) in 1990 to 885.07 per 100,000 people (95% UI: 755.93–1,029.39) in 2021, a decrease of 71%. Similarly, the ASIR of childhood diarrhea diseases in 2021 also gradually increased with age. Compared with 1990, the ASDR of childhood diarrhea diseases in both males and females in 2021 has decreased significantly. However, analyzing the data, it can be found that the vast majority of childhood diarrhea disease deaths are mostly concentrated in children under 5 years old. However, a significant decline in the ASDR of diarrhea diseases was also observed in children under 5 years old. Compared to 1990, DALYs demonstrated a downward trend by 2021, with the most pronounced reduction occurring among children under 5 years of age. However, no notable differences were found between genders (Figure 2, Table 1, Supplementary Tables S1–S3).

### Regional level

The global burden of childhood diarrhea diseases demonstrates notable regional disparities, aligning largely with variations in SDI levels. The ASPR displays marked differences across regions. Its prevalence reaches a peak of 1,858.95 per 100,000 individuals (95% UI: 1,562.06–2,140.03) in low SDI areas, whereas it drops to its lowest point, 434.32 per 100,000 individuals (95% UI: 315.22–563.25), within middle-high SDI regions. The temporal trends of ASPR illustrate divergent trajectories across SDI categories, potentially reflecting distinct phases of epidemiological transition. A particularly substantial decline is observed in the middle SDI regions, reflected by an EAPC of −2.54 (95% CI: −2.65 to −2.43). Specifically, the ASPR within these areas contracted from 2,017.77 per 100,000 individuals (95% UI: 1,664.97–2,410.14) in 1990 to 923.36 per 100,000 individuals (95% UI: 734.65–1,126.47) by 2021, underscoring the efficacy of implemented prevention and management strategies. In comparison, high SDI regions experienced only a minimal decline in EAPC, measured at −0.27 (95% CI: −0.66–0.13), suggesting that the burden of childhood diarrhea diseases has remained relatively stable in these areas. This tendency may indicate the intricate interplay of various contributing factors (Figure 2, Supplementary Table S1).

The ASIR and ASDR accentuate these regional variations. Data reveals that low SDI regions report the highest ASIR and ASDR, while middle-high SDI regions report the lowest ASIR, and high SDI regions report the lowest ASDR. Specifically, the ASIR in low SDI regions stands at 125,446.53 per 100,000 individuals (95% UI: 102,408.76–149,179.47), compared to 28,676.51 per 100,000 individuals (95% UI: 20,624.6–37,605.11) in middle-high SDI regions. Furthermore, the ASDR in high SDI regions is documented at 0.28 per 100,000 individuals (95% UI: 0.24–0.31), while in low SDI regions, the ASDR is significantly higher at 53.31 per 100,000 individuals (95% UI: 38.36–72.95) (Figure 2, Table 1, Supplementary Table S2).

The disease burden quantified through the age-standardized DALY rate highlights the pronounced disparities across regions. The heaviest burden is observed in low SDI regions, with a DALY rate of 4,915.84 per 100,000 individuals (95% UI: 3,589.18–6,613.38), while the lightest burden is recorded in high SDI regions, at 80.55 per 100,000 individuals (95% UI: 57.58–108.46) (Figure 2, Supplementary Table S3).

**Figure 3:**
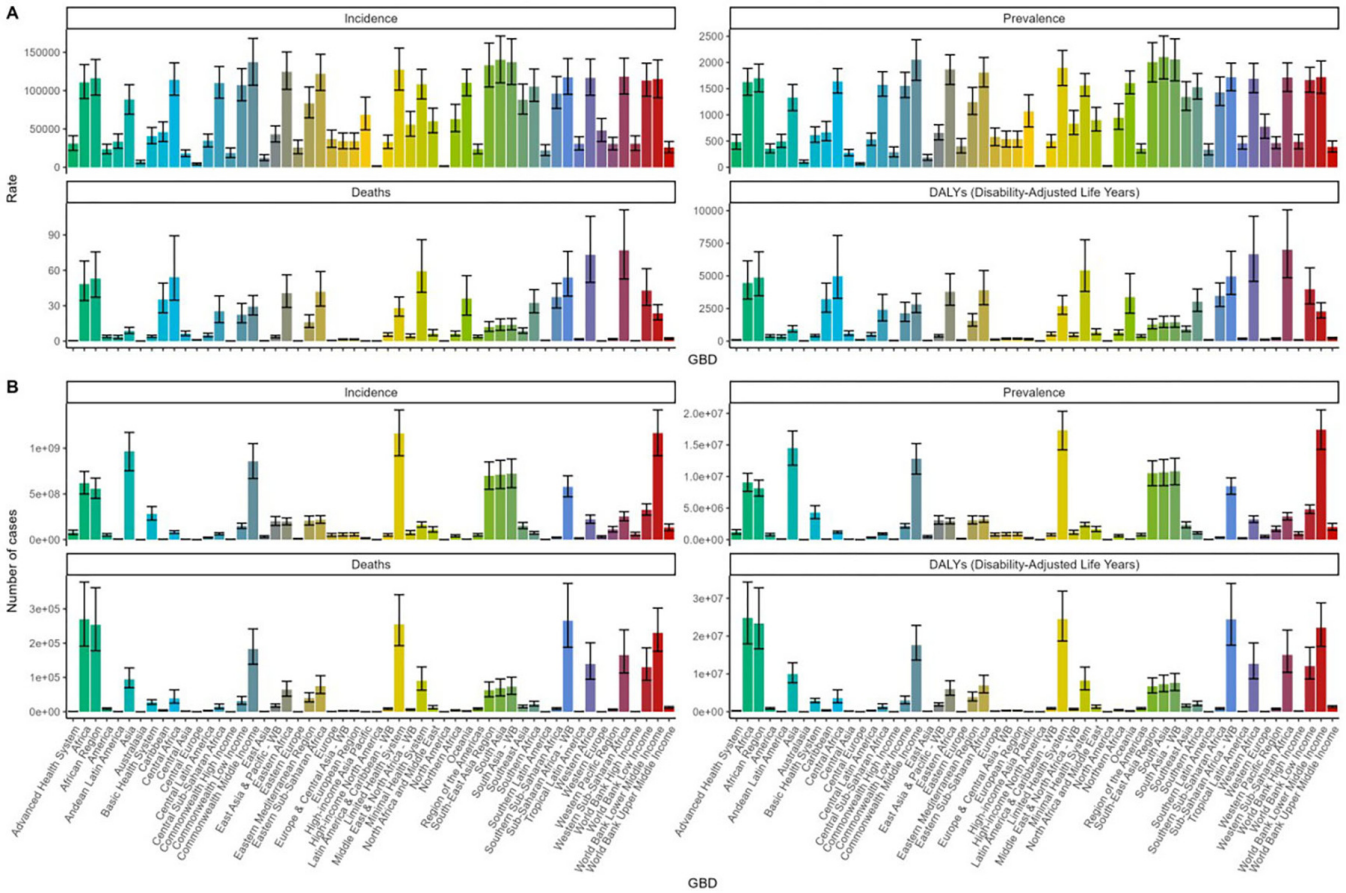
Epidemiological changes of children diarrhea diseases stratified by GBD regions in 2021: **(A)** Incidence, prevalence, mortality, and DALY rates; **(B)** Number of incident cases, prevalent cases, deaths, and DALYs. DALY, disability-adjusted life-year.

Our findings reveal that the South Asian region bears the highest prevalence of childhood diarrhea diseases on a global scale. Specifically, South Asia registers the highest ASPR, reaching 2,104.21 per 100,000 individuals (95% UI: 1,686.32–2,505.35). This region is closely followed by the Commonwealth middle-income region, with an ASPR of 2,052.72 per 100,000 individuals (95% UI: 1,659.43–2,435.71). Southeast Asia also exhibits a notably high ASPR of 2,006.92 per 100,000 individuals (95% UI: 1,627.42–2,379.65), ranking fourth among the GBD regions (Figure 3, Supplementary Table S1).

The findings indicate that South Asia ranks among the regions with the highest global ASIR. Specifically, South Asia reports an ASIR of 140,235.11 per 100,000 individuals (95% UI: 110,138.57–171,297.22), while the Commonwealth middle-income region records a comparable rate of 137,201.22 per 100,000 individuals (95% UI: 107,027.55–168,185.85). These values represent the highest and second-highest ASIRs, respectively. Conversely, the lowest ASIR is observed in high-income North America, at 1,496.63 per 100,000 individuals (95% UI: 1,146.69–1,927.62).Moreover, the temporal trend from 1990–2021 reveals substantial variation in the rates of increase and decline across different regions. The most pronounced increase is noted in the high-income Asia-Pacific region, which is the only area exhibiting growth, with an EAPC of 1.46 (95% CI: 1.11–1.82). Conversely, the steepest decline is found in high-income North America, where the EAPC stands at −7.58 (95% CI: −8.46 to −6.71) (Figure 3, Supplementary Table S4).

The age-standardized death rate (ASDR) associated with childhood diarrhea diseases is notably higher in West Africa, Central Africa, sub-Saharan Africa, and its western regions, surpassing 50 per 100,000 individuals. Interestingly, between 1990 and 2021, the ASDR in Western Europe displayed the most pronounced rise, with an EAPC of 0.81 (95% CI: −0.08–1.71), whereas the steepest decline was observed in East Asia, where the EAPC reached −13.15 (95% CI: −13.44 to −12.86) (Figure 3, Supplementary Table S2).

The highest age-standardized DALY rates are concentrated in the western region of sub-Saharan Africa, recorded at 7,003.5 per 100,000 individuals (95% UI: 4,853.98–10,059.29), followed by West Africa at 6,665.63 per 100,000 individuals (95% UI: 4,580.33–9,569.76), and Central Africa at 4,973.7 per 100,000 individuals (95% UI: 3,286.7–8,100.97). Between 1990 and 2021, the age-standardized DALY rate of childhood diarrhea diseases experienced the most pronounced increase in the high-income Asia-Pacific region, with an EAPC of 0.49 (95% CI: 0.2–0.78), while the steepest decline occurred in East Asia, with an EAPC of −11.88 (95% CI: −12.22 to −11.54) (Figure 3, Supplementary Table S3).

**Figure 4:**
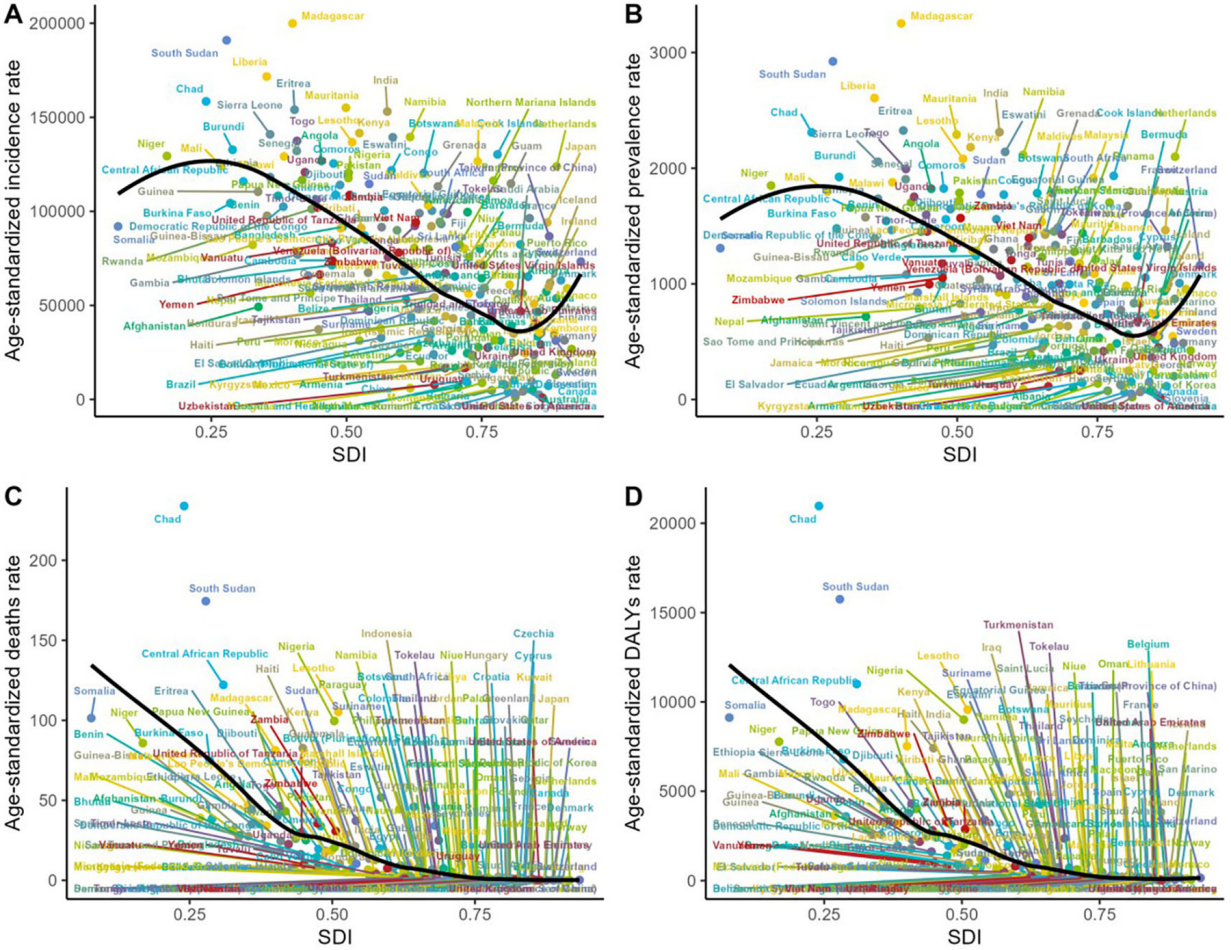
The relationship among the incidence, prevalence, mortality, disability and SDI of children diarrheal diseases in 2021. **(A)** The relationship between incidence and SDI. **(B)** The relationship between prevalence and SDI. **(C)** The relationship between mortality and SDI. **(D)** The relationship between DALYs rate and SDI. SDI, socio-demographic index; DALY, disability-adjusted life-year.

A bidirectional relationship exists between ASIR, ASPR, and SDI. On the one hand, these indicators tend to decline progressively as SDI improves, yet a trend of resurgence can be observed in regions with high SDI. Conversely, ASDR and the age-standardized DALY rate exhibit a negative correlation with SDI (Figure 4).

### National level

**Figure 5:**
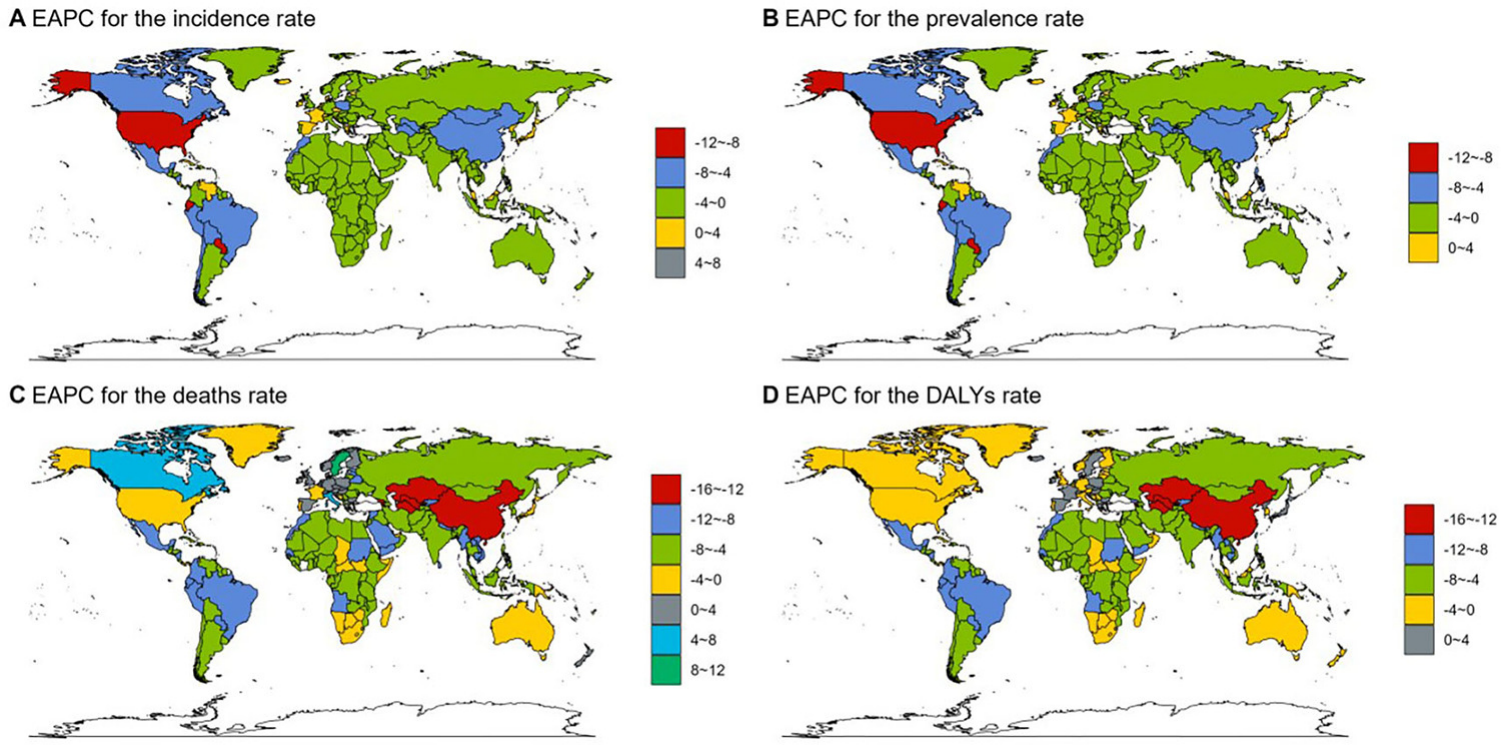
The EAPC of incidence, prevalence, mortality and DALYs rate of childhood diarrheal diseases in 204 countries and regions. **(A)** EAPC of incidence. **(B)** EAPC of prevalence. **(C)** EAPC of mortality. **(D)** EAPC of DALYs rate.

**Figure 6:**
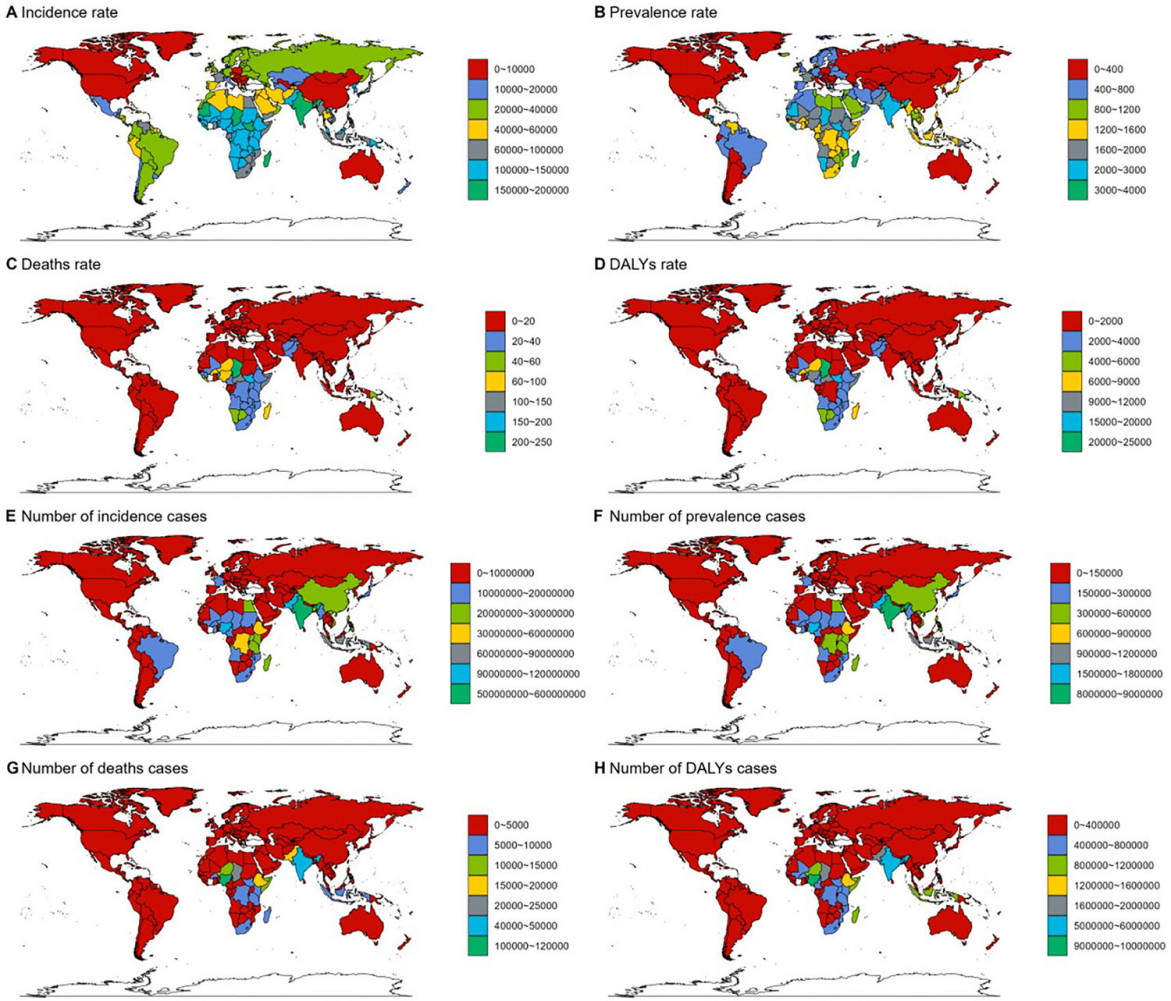
Epidemiological situation of children diarrhea diseases in 204 countries and regions in 2021: **(A–D)** incidence, prevalence, mortality and DALYs rate of childhood diarrhea in 204 countries and regions. **(E–H)** Number of cases, number of patients, number of deaths and number of disability-adjusted life years of children diarrhea diseases in 204 countries and regions. DALY, disability-adjusted life-year.

The ASPR of children diarrhea diseases ranges from 15.32–3,251.11 per 100,000 individuals. Among all countries, the highest ASPR is observed in Madagascar (3,251.11 per 100,000 individuals; 95% UI: 2,915.43–3,555.17), followed by South Sudan (2,922.97 per 100,000 individuals; 95% UI: 2,510.19–3,346.91) and Liberia (2,606.01 per 100,000 individuals; 95% UI: 2,209.45–2,954.55). It is noteworthy that these three countries, which report the highest ASPR, are all situated in Africa—Madagascar in southeastern Africa, South Sudan in East Africa, and Liberia in West Africa.The country with the most significant increase in ASPR of childhood diarrhea diseases is Taiwan, China (EAPC 3.85, 95% CI 3.46–4.24), and the country with the most significant decrease is the United States (EAPC −10.18, 95% CI −11.64 to −8.7) (Figures 5, 6, Supplementary Table S1).

Madagascar also reports the highest ASIR of childhood diarrhea diseases, with 199,871.61 per 100,000 individuals (95% UI: 178,926.12–217,359.68), whereas the lowest ASIR is recorded in the United States, at 895.21 per 100,000 individuals (95% UI: 707.63–1,136.65) (Figure 6A, Supplementary Table S3).

Based on the analysis of ASDR and DALY data for childhood diarrhea diseases in 2021, a clear alignment emerges among the countries most severely impacted. Chad, South Sudan, the Central African Republic, Lesotho, Somalia, Nigeria, and Niger are prominent in both indicators, holding identical ranks and occupying the top seven positions. Chad ranks first in both measures, reporting the highest ASDR at 233.98 per 100,000 individuals (95% UI: 157.22–393.9) and the highest DALY rate at 20,966.29 per 100,000 individuals (95% UI: 14,221.09–35,113.4). Notably, all these countries are situated in Africa. This outcome aligns with regional analyses, which highlight large areas of the continent—particularly West Africa, Central Africa, sub-Saharan Africa, and its western regions—as experiencing the highest global rates of childhood diarrhea disease mortality and DALY rates. The alignment between national and regional data reinforces the substantial challenges posed by childhood diarrhea diseases within African countries. In Uzbekistan (EAPC −13.09, 95% CI: −13.92 to −12.25), Equatorial Guinea (EAPC −13, 95% CI: −13.55 to −12.45), and Turkmenistan (EAPC −12.55, 95% CI: −13.16 to −11.94), the age-standardized number of DALYs attributed to childhood diarrhea diseases exhibited the most substantial decline (Figures 5, 6, Supplementary Tables S2, S3).

In Qatar, the number of cases surged by a remarkable 216%, representing the most significant increase, whereas the United States experienced the steepest reduction, with a decline of approximately 94%. A similar trend is observed in incidence rates, with Qatar showing the highest increase, rising by 223%, and the United States showing the most significant decrease, by 95%. Regarding mortality, Canada reported the largest rise in deaths, increasing by 171%. Meanwhile, Qatar recorded the same number of deaths from childhood diarrhea diseases in 2021 as in 1990, showing no noticeable change. Some countries, including the US Virgin Islands, reported no deaths from these diseases in 2021. Lastly, the Netherlands experienced the most substantial rise in DALYs, increasing by 71%, while Armenia recorded the greatest reduction, by 99% (Figure 6, Supplementary Tables S1–S3).

### Risk factors for childhood diarrhea diseases

This study gathered data on deaths and DALYs associated with childhood diarrhea diseases, which were attributed to 15 risk factors, including ambient particulate matter pollution, child stunting, child underweight, child wasting, discontinued breastfeeding, household air pollution from solid fuels, low birth weight for gestational age, lack of access to handwashing facilities, non-exclusive breastfeeding, preterm birth for weight, unsafe sanitation, unsafe water sources, vitamin A deficiency, and zinc deficiency. Additionally, the data were stratified according to SDI regions.

**Figure 7:**
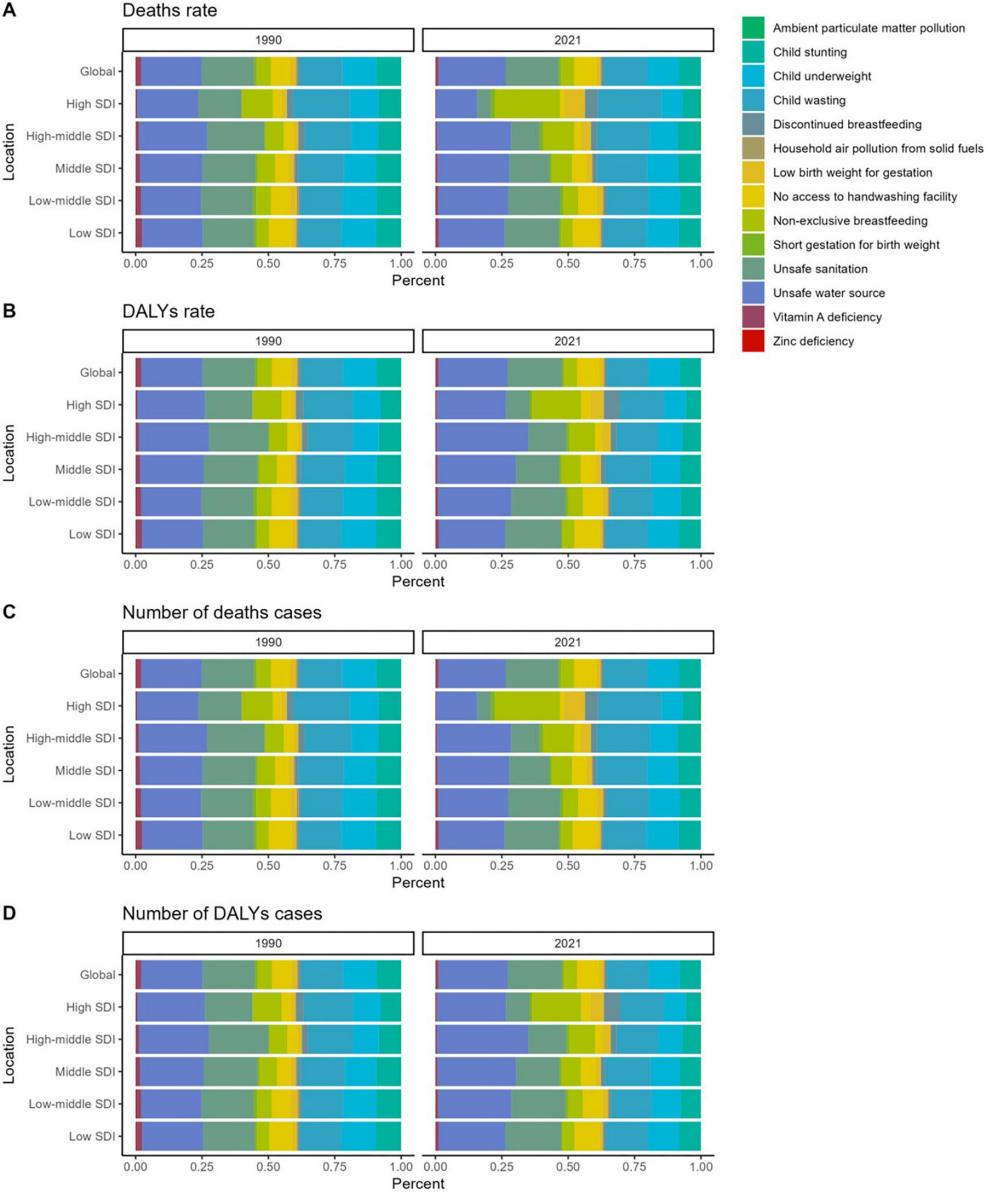
Comparative analysis of the proportion of risk factors for diarrhea disease burden among children in 1990 vs. 2021. **(A)** The proportion of risk factors to diarrhea disease-related mortality among children. **(B)** The proportion of risk factors to diarrhea disease-related DALYs rate among children. **(C)** Proportional contribution of risk factors to diarrhea disease-related number of deaths cases among children. **(D)** Proportional contribution of risk factors to diarrhea disease-related number of DALYs cases among children.

Between 1990 and 2021, unsafe water sources consistently remained the most significant contributor to childhood diarrhea disease-related deaths globally, with their proportion continuing to increase compared to 1990. In 2021, unsafe water sources were responsible for 25.3% of all childhood diarrhea deaths worldwide. Among the five SDI regions, the highest proportion of deaths attributed to unsafe water sources was noted in middle-high SDI regions, accounting for 28%, while the lowest was recorded in high SDI regions, at 15.4%. In high SDI regions, non-exclusive breastfeeding and child wasting were identified as the leading causes of death, whereas in the other four SDI regions, unsafe water sources remained the primary cause. The findings from the analysis of mortality proportions across global and regional levels align closely, and thus, further repetition here is unnecessary (Figure 7, Supplementary Table S4).

Unsafe water sources remain the primary factor influencing DALYs associated with childhood diarrhea diseases, a trend that has shown little change between 1990 and 2021. In 2021, unsafe water sources accounted for 26.0% of the global DALY rate, ranking first across all SDI regions. The highest proportion was recorded in middle-high SDI regions, at 34.5%, while the lowest proportion, 26.1%, was observed in high SDI regions. It is anticipated that unsafe water sources will likely continue to be the most significant factor contributing to global childhood diarrhea disease-related deaths and DALYs in the future (Figure 7, Supplementary Table S4).

## Discussion

This investigation examined the incidence, mortality, and DALYs associated with diarrhea diseases among children aged 0–14 years across all GBD regions and countries from 1990–2021. Our comprehensive analysis reveals a complex landscape of disparities and trends across regions and sociodemographic indices. Globally, the age-standardized prevalence rate (ASPR), incidence rate (ASIR), death rate (ASDR), and DALY rate for childhood diarrhea diseases exhibited a decline between 1990 and 2021. This reduction can be attributed to several key public health advances: Expanded access to improved water sources and sanitation facilities, coupled with hygiene promotion programs (including handwashing with soap), have reduced pathogen exposure and transmission globally. The introduction and scaling up of rotavirus vaccination programs since the mid-2000s substantially reduced the incidence of the leading viral cause of severe childhood diarrhea (16). Crucially, wider availability and utilization of low-osmolarity oral rehydration solution (ORS) and zinc supplementation for treatment have dramatically decreased case fatality rates (17). Enhanced healthcare access, strengthened primary care systems, and socioeconomic development, including increased maternal education and poverty reduction, further contributed to these gains. This finding of decreasing age-standardized rates aligns with previous Global Burden of Disease (GBD) studies (1, 18, 19), which also attribute declines to these multifaceted interventions.

Despite this progress, the absolute burden remains substantial. The global number of deaths and DALYs, while declining, underscores that diarrhea diseases continue to pose a significant public health challenge for children. Furthermore, the distribution of DALYs remained relatively stable throughout much of the study period. Critically, diarrhea diseases consistently ranked among the 25 leading tertiary causes of DALYs in 2021. It is also evident that the global burden is significantly underestimated when considering the long-term sequelae of diarrhea diseases and their detrimental impact on child development. Enhancing understanding of the epidemiology of childhood diarrhea diseases, including prevalence and characteristics across different age groups under 14 and varying country income levels over the past three decades, is therefore essential for effective prevention and control efforts.

Additionally, a notable negative association was identified between SDI and the incidence, mortality, and DALYs linked to diarrhea diseases. According to the 2021 GBD data, diarrhea diseases rank as the eighth leading cause of DALYs in low-middle SDI regions (4, 20). Globally, the burden of these diseases is predominantly concentrated in Western sub-Saharan Africa, South Asia, Central sub-Saharan Africa, and several regions listed among the top ten level 3 causes by DALY rate. The most substantial reductions in childhood diarrhea disease-related mortality and DALYs have been observed in low-middle and low SDI regions. This decline can be attributed not only to the high baseline prevalence and epidemiological burden in these regions but also to the implementation of various intervention strategies, including WASH infrastructure improvements (e.g., community water access and sanitation) which reduced transmission by 3%–50% in trial settings (21), alongside expanded oral rehydration salts (ORS) and zinc supplementation that cut mortality by up to 93% at >60% coverage. Further reductions were driven by rotavirus vaccination preventing 28%–61% of severe cases in Gavi-supported countries (22), and community health worker programs promoting breastfeeding and hygiene practices. These efforts were amplified through coordinated partnerships involving WHO and UNICEF's Diarrhoeal Disease Control Programme, Gavi's vaccine delivery systems, and World Bank-funded WASH infrastructure. Despite progress, subregional disparities persist due to fragmented primary care access.

Identifying the risk factors associated with diarrhea diseases is essential for alleviating their burden, particularly in low- and middle-income countries. Among the 15 risk factors examined in this study, unsafe water sources consistently emerged as the most significant contributor to childhood diarrhea disease-related deaths and DALYs from 1990–2021 (23). In these countries, one-tenth of deaths among children under five have been prevented through various diarrhea intervention strategies, which have contributed to a reduction in overall diarrhea-related mortality. Between 1990 and 2017, the mortality rate from diarrhea among children under five worldwide declined by 69.6% per 100,000 people. The most substantial reductions were achieved by minimizing exposure to unsafe sanitation facilities (a 13.3% decrease) and reducing child wasting (a 9.9% decrease), along with increasing the use of oral rehydration therapy (a 6.9% improvement) (24, 25).

Diarrhea diseases may impair nutrient absorption by inducing inflammation or causing the flattening of intestinal microvilli (26, 27), which can result in malnutrition among children. A bidirectional relationship exists between malnutrition and diarrhea in children, with each condition increasing the risk of the other. Evidence suggests that childhood malnutrition may lead to long-term consequences, including cognitive impairment, reduced physical fitness in adulthood, diminished vaccine responsiveness, and even chronic conditions such as cardiovascular disease and type 2 diabetes later in life (28–30). It can also impact growth, resulting in short stature. Prioritizing the prevention of diarrhea and intestinal infections—through the provision of safe water, improved sanitation, effective vaccination programs, and treatments such as oral rehydration therapy—has likely contributed significantly to progress in reducing the burden of these diseases (3, 31–33).

## Limitations

This investigation is susceptible to numerous critical constraints. The analysis primarily depended on data from the GBD database, whose accuracy is restricted by the availability of national registry records, the substantial quantity of undetected childhood diarrhea cases, and the lack of detailed information on other risk factors linked to childhood diarrhea.

## Conclusion

In the past 30 years, the burden of childhood diarrhea diseases globally has been decreasing year by year, but it still occupies an important position in DALYs. The results of this cross-sectional study show that although the changes in EAPC in childhood diarrhea disease-related mortality and childhood diarrhea disease-related DALYs are unbalanced worldwide. In low-middle SDI regions, childhood diarrhea disease-related mortality and childhood diarrhea disease-related DALYs have been decreasing year by year but are still relatively high. Our analysis identifies unsafe water sources as the predominant risk factor globally except in high-SDI regions where non-exclusive breastfeeding and child wasting dominate. Therefore, health care professionals urgently need to develop more cost-effective and targeted strategies prioritizing water safety infrastructure and targeted feeding programs to reduce the morbidity and mortality related to childhood diarrhea diseases, reduce the socioeconomic burden, and avoid corresponding risks.

## Data Availability

The data that support the findings of this study are available from the Global Burden of Disease Study 2021 (GBD 2021).

## References

[1] GBD 2017 Diarrhoeal Disease Collaborators. Quantifying risks and interventions that have affected the burden of diarrhoea among children younger than 5 years: an analysis of the global burden of disease study 2017. Lancet Infect Dis. (2020) 20:37–59. 10.1016/S1473-3099(19)30401-3 31678029 PMC7340495

[2] Schlaudecker EP Steinhoff MC Moore SR. Interactions of diarrhea, pneumonia, and malnutrition in childhood: recent evidence from developing countries. Curr Opin Infect Dis. (2011) 24:496–502. 10.1097/QCO.0b013e328349287d 21734569 PMC5454480

[3] Troeger C Colombara DV Rao PC Khalil IA Brown A Brewer TG Global disability-adjusted life-year estimates of long-term health burden and undernutrition attributable to diarrhoeal diseases in children younger than 5 years. Lancet Glob Health. (2018) 6:e255–69. 10.1016/S2214-109X(18)30045-7 29433665 PMC5861379

[4] GBD 2021 Diseases and Injuries Collaborators. Global incidence, prevalence, years lived with disability (YLDs), disability-adjusted life-years (DALYs), and healthy life expectancy (HALE) for 371 diseases and injuries in 204 countries and territories and 811 subnational locations, 1990–2021: a systematic analysis for the global burden of disease study 2021. Lancet. (2024) 403:2133–61. 10.1016/S0140-6736(24)00757-8 38642570 PMC11122111

[5] GBD 2021 Diabetes Collaborators. Global, regional, and national burden of diabetes from 1990 to 2021, with projections of prevalence to 2050: a systematic analysis for the global burden of disease study 2021. Lancet. (2023) 402:203–34. 10.1016/S0140-6736(23)01301-6 37356446 PMC10364581

[6] GBD 2019 Diseases and Injuries Collaborators. Global burden of 369 diseases and injuries in 204 countries and territories, 1990–2019: a systematic analysis for the global burden of disease study 2019. Lancet. (2020) 396:1204–22. 10.1016/S0140-6736(20)30925-9 33069326 PMC7567026

[7] GBD 2021 Risk Factors Collaborators. Global burden and strength of evidence for 88 risk factors in 204 countries and 811 subnational locations, 1990–2021: a systematic analysis for the global burden of disease study 2021. Lancet. (2024) 403:2162–203. 10.1016/S0140-6736(24)00933-4 38762324 PMC11120204

[8] Iqbal U, GBD 2019 Demographics. Collaborators global age-sex-specific fertility, mortality, healthy life expectancy (HALE), and population estimates in 204 countries and territories, 1950–2019: a comprehensive demographic analysis for the global burden of disease study 2019. Lancet. (2020) 396:1160–203. 10.1016/S0140-6736(20)30977-6 33069325 PMC7566045

[9] GBD 2016 Mortality Collaborators. Global, regional, and national under-5 mortality, adult mortality, age-specific mortality, and life expectancy, 1970–2016: a systematic analysis for the global burden of disease study 2016. Lancet. (2017) 390:1084–150. 10.1016/S0140-6736(17)31833-0 28919115 PMC5605514

[10] Degenhardt L Charlson F Ferrari A Santomauro D Erskine H Mantilla-Herrara A The global burden of disease attributable to alcohol and drug use in 195 countries and territories, 1990–2016: a systematic analysis for the global burden of disease study 2016. Lancet Psychiatry. (2018) 5:987––1012. 10.1016/S2215-0366(18)30337-7 30392731 PMC6251968

[11] Troeger C Blacker BF Khalil IA Rao PC Cao S Zimsen SR Estimates of the global, regional, and national morbidity, mortality, and aetiologies of diarrhoea in 195 countries: a systematic analysis for the global burden of disease study 2016. Lancet Infect Dis. (2018) 18:1211–28. 10.1016/S1473-3099(18)30362-1 30243583 PMC6202444

[12] GBD 2021 Diarrhoeal Diseases Collaborators. Global, regional, and national age-sex-specific burden of diarrhoeal diseases, their risk factors, and aetiologies, 1990–2021, for 204 countries and territories: a systematic analysis for the global burden of disease study 2021. Lancet Infect Dis. (2024) 25:S1473-3099(24)00691-1. 10.1016/S1473-3099(24)00691-1 39708822 PMC12018300

[13] GBD Diarrhoeal Diseases Collaborators. Estimates of global, regional, and national morbidity, mortality, and aetiologies of diarrhoeal diseases: a systematic analysis for the global burden of disease study 2015. Lancet Infect Dis. (2017) 17:909–48. 10.1016/S1473-3099(17)30276-1 28579426 PMC5589208

[14] Deng Y Zhao P Zhou L Xiang D Hu J Liu Y Epidemiological trends of tracheal, bronchus, and lung cancer at the global, regional, and national levels: a population-based study. J Hematol Oncol. (2020) 13:98. 10.1186/s13045-020-00915-0 32690044 PMC7370495

[15] Clegg LX Hankey BF Tiwari R Feuer EJ Edwards BK. Estimating average annual per cent change in trend analysis. Stat Med. (2009) 28:3670–82. 10.1002/sim.3733 19856324 PMC2843083

[16] Tate JE Burton AH Boschi-Pinto C Parashar UD, World Health Organization–Coordinated Global Rotavirus Surveillance Network. Global, regional, and national estimates of rotavirus mortality in children <5 years of age, 2000–2013. Clin Infect Dis. (2016) 62(2):S96–S105. 10.1093/cid/civ1013 27059362 PMC11979873

[17] Lazzerini M Ronfani L. Oral zinc for treating diarrhoea in children. Cochrane Database Syst Rev. (2013) 1:CD005436. 10.1002/14651858.CD005436.pub4 23440801

[18] Feigin VL Forouzanfar MH Krishnamurthi R Mensah GA Connor M Bennett DA Global and regional burden of stroke during 1990–2010: findings from the global burden of disease study 2010. Lancet. (2014) 383:245–54. 10.1016/s0140-6736(13)61953-4 24449944 PMC4181600

[19] GBD 2015 Neurological Disorders Collaborator Group. Global, regional, and national burden of neurological disorders during 1990–2015: a systematic analysis for the global burden of disease study 2015. Lancet Neurol. (2017) 16:877–97. 10.1016/S1474-4422(17)30299-5 28931491 PMC5641502

[20] Li J Gao Z Bai H Wang W Li Y Lian J Global, regional, and national total burden related to hepatitis B in children and adolescents from 1990 to 2021. BMC Public Health. (2024) 24:2936. 10.1186/s12889-024-20462-4 39443929 PMC11515762

[21] Prüss-Ustün A Wolf J Bartram J Clasen T Cumming O Freeman MC Burden of disease from inadequate water, sanitation and hygiene for selected adverse health outcomes: an updated analysis with a focus on low- and middle-income countries. Int J Hyg Environ Health. (2019) 222(5):765–77. 10.1016/j.ijheh.2019.05.004 31088724 PMC6593152

[22] Clark A van Zandvoort K Flasche S Sanderson C Bines J Tate J Efficacy of live oral rotavirus vaccines by duration of follow-up: a meta-regression of randomised controlled trials. Lancet Infect Dis. (2019) 19(7):717–27. 10.1016/S1473-3099(19)30126-4 31178289 PMC6595176

[23] Mebrahtom S Worku A Gage DJ. The risk of water, sanitation and hygiene on diarrhea-related infant mortality in eastern Ethiopia: a population-based nested case-control. BMC Public Health. (2022) 22:343. 10.1186/s12889-022-12735-7 35177054 PMC8855567

[24] Keddy KH Saha S Okeke IN Kalule JB Qamar FN Kariuki S. Combating childhood infections in LMICs: evaluating the contribution of big data big data, biomarkers and proteomics: informing childhood diarrhoeal disease management in low- and middle-income countries. EBioMedicine. (2021) 73:103668. 10.1016/j.ebiom.2021.103668 34742129 PMC8579132

[25] Li T Qiang N Bao Y Li Y Zhao S Chong KC Global burden of enteric infections related foodborne diseases, 1990–2021: findings from the global burden of disease study 2021. Sci One Health. (2024) 3:100075. 10.1016/j.soh.2024.100075 39282625 PMC11402448

[26] Guerrant RL Bolick DT Swann JR. Modeling enteropathy or diarrhea with the top bacterial and protozoal pathogens: differential determinants of outcomes. ACS Infect Dis. (2021) 7:1020–31. 10.1021/acsinfecdis.0c00831 33901398 PMC8154416

[27] Adams N Dhimal M Mathews S Iyer V Murtugudde R Liang X-Z El Niño southern oscillation, monsoon anomaly, and childhood diarrheal disease morbidity in Nepal. PNAS Nexus. (2022) 1:pgac032. 10.1093/pnasnexus/pgac032 36713319 PMC9802392

[28] Pinkerton R Oriá RB Lima AAM Rogawski ET Oriá MOB Patrick PD Early childhood diarrhea predicts cognitive delays in later childhood independently of malnutrition. Am J Trop Med Hyg. (2016) 95:1004–10. 10.4269/ajtmh.16-0150 27601523 PMC5094207

[29] Qadri F Bhuiyan TR Sack DA Svennerholm A-M. Immune responses and protection in children in developing countries induced by oral vaccines. Vaccine. (2013) 31:452–60. 10.1016/j.vaccine.2012.11.012 23153448

[30] Victora CG Adair L Fall C Hallal PC Martorell R Richter L Maternal and child undernutrition: consequences for adult health and human capital. Lancet. (2008) 371:340–57. 10.1016/S0140-6736(07)61692-4 18206223 PMC2258311

[31] Das JK Salam RA Bhutta ZA. Global burden of childhood diarrhea and interventions. Curr Opin Infect Dis. (2014) 27:451–8. 10.1097/QCO.0000000000000096 25101554

[32] Munos MK Walker CLF Black RE. The effect of oral rehydration solution and recommended home fluids on diarrhoea mortality. Int J Epidemiol. (2010) 39(Suppl 1):i75–87. 10.1093/ije/dyq025 20348131 PMC2845864

[33] Hutton G Chase C. The knowledge base for achieving the sustainable development goal targets on water supply, sanitation and hygiene. Int J Environ Res Public Health. (2016) 13:536. 10.3390/ijerph13060536 27240389 PMC4923993

