# Supplementary material for: The global burden of childhood diarrhea and its epidemiological characteristics from 1990 to 2021
Source: Front Pediatr. 2025 Aug 28;13:1656234. doi: 10.3389/fped.2025.1656234 (PMC12423085; doi:10.3389/fped.2025.1656234)
Supplement: Supplementary file 1 [file Table1.doc]

**eTable 1. Prevalence of diarrhea disease in children at the global, sex, age-group levels, regional and national levels from 1990 to 2021.**

| **Location** | **Rate per 100 000(95% UI)** |  |  |  |  |
| --- | --- | --- | --- | --- | --- |
|  | **1990** | | **2021** | | **1990-2021** |
|  | **Prevalence cases** | **Prevalence rate** | **Prevalence cases** | **Prevalence rate** | **EAPC** |
| Global | 38935022 (32438782-45732521) | 2238.74 (1865.21-2629.59) | 25190229 (20770723-29673552) | 1252.09 (1032.41-1474.93) | -1.72 (-1.92--1.52) |
| Sex |  |  |  |  |  |
| Female | 19389226 (16176251-22803776) | 2292.79 (1912.86-2696.57) | 12589356 (10422511-14710228) | 1292.95 (1070.41-1510.76) | -1.7 (-1.88--1.53) |
| Male | 19545796 (16240910-23147117) | 2187.57 (1817.69-2590.64) | 12600873 (10321243-14940378) | 1213.76 (994.18-1439.11) | -1.74 (-1.96--1.52) |
| Age |  |  |  |  |  |
| <5 years | 19458645 (17043262-22054325) | 3138.81 (2749.19-3557.51) | 5825270 (4975326-6775131) | 885.07 (755.93-1029.39) | NA |
| 5-9 years | 11133830 (8121785-14663667) | 1908.01 (1391.83-2512.92) | 8134704 (6206924-10183283) | 1184 (903.41-1482.17) | NA |
| 10-14 years | 8342546 (5908444-11233370) | 1557.37 (1102.97-2097.02) | 11230255 (8645948-14162914) | 1684.62 (1296.95-2124.54) | NA |
| SDI region |  |  |  |  |  |
| High-middle SDI | 2671092 (2124868-3269584) | 976.19 (776.57-1194.92) | 1002795 (727814-1300501) | 434.32 (315.22-563.25) | -2.24 (-2.53--1.94) |
| High SDI | 1186785 (854654-1584624) | 638.72 (459.97-852.83) | 833574 (601736-1084025) | 483.13 (348.76-628.29) | -0.27 (-0.66-0.13) |
| Low-middle SDI | 15187144 (12714932-17771633) | 3216.85 (2693.2-3764.28) | 9549793 (7838371-11342801) | 1646.97 (1351.81-1956.19) | -2.11 (-2.24--1.98) |
| Low SDI | 8222674 (7073787-9308728) | 3592.04 (3090.16-4066.48) | 8555384 (7188979-9848946) | 1858.95 (1562.06-2140.03) | -1.98 (-2.22--1.74) |
| Middle SDI | 11646857 (9610425-13911648) | 2017.77 (1664.97-2410.14) | 5234159 (4164460-6385513) | 923.36 (734.65-1126.47) | -2.54 (-2.65--2.43) |
| GBD region |  |  |  |  |  |
| Advanced Health System | 2204807 (1666444-2810709) | 758.95 (573.64-967.52) | 1212980 (867333-1576932) | 482.83 (345.24-627.7) | -0.87 (-1.25--0.48) |
| Africa | 9790745 (8522229-10985684) | 3481.46 (3030.39-3906.36) | 9050157 (7650676-10509921) | 1625.36 (1374.02-1887.52) | -2.4 (-2.68--2.12) |
| African Region | 8572467 (7427368-9651510) | 3704.94 (3210.04-4171.29) | 8135442 (6886222-9431655) | 1699.93 (1438.9-1970.78) | -2.48 (-2.75--2.21) |
| America | 2977272 (2474254-3542011) | 1356.87 (1127.63-1614.25) | 791879 (610848-998844) | 355.86 (274.51-448.87) | -4.53 (-4.74--4.32) |
| Andean Latin America | 479250 (431531-530114) | 3226.83 (2905.54-3569.31) | 89123 (68503-114221) | 492.53 (378.58-631.24) | -6.42 (-6.86--5.97) |
| Asia | 24555308 (20181316-29325603) | 2315.08 (1902.7-2764.82) | 14515054 (11781460-17217766) | 1331.71 (1080.91-1579.68) | -1.61 (-1.75--1.47) |
| Australasia | 16879 (12438-22206) | 368.06 (271.23-484.21) | 6113 (4517-7960) | 106.66 (78.82-138.88) | -3.34 (-3.72--2.95) |
| Basic Health System | 12569068 (10428484-14910613) | 1700.49 (1410.89-2017.29) | 4274895 (3319212-5378349) | 614.77 (477.33-773.45) | -3.23 (-3.38--3.09) |
| Caribbean | 134372 (114252-156787) | 1177.42 (1001.12-1373.83) | 76625 (58362-100833) | 666.01 (507.27-876.42) | -1.21 (-1.78--0.63) |
| Central Africa | 1010322 (896560-1125770) | 3251.76 (2885.61-3623.33) | 1177472 (1016707-1353106) | 1638.79 (1415.04-1883.24) | -2.03 (-2.44--1.63) |
| Central Asia | 317304 (283620-351775) | 1269.66 (1134.88-1407.59) | 77116 (60591-94484) | 278.64 (218.93-341.39) | -4.68 (-4.82--4.54) |
| Central Europe | 50610 (39886-62482) | 171.65 (135.28-211.92) | 12194 (9294-15406) | 68.89 (52.5-87.03) | -2.45 (-2.88--2.02) |
| Central Latin America | 1128011 (942728-1342499) | 1752.08 (1464.29-2085.23) | 337225 (265681-415095) | 531.19 (418.5-653.85) | -4.15 (-4.36--3.95) |
| Central Sub-Saharan Africa | 813486 (728305-905783) | 3215.54 (2878.83-3580.37) | 923986 (796363-1070941) | 1574.57 (1357.09-1825) | -2.26 (-2.67--1.86) |
| Commonwealth High Income | 149615 (105250-203198) | 653.66 (459.83-887.76) | 72887 (50398-98709) | 287.79 (198.99-389.75) | -2.07 (-2.88--1.26) |
| Commonwealth Low Income | 3510430 (3122797-3933240) | 3636.39 (3234.85-4074.37) | 2171170 (1856889-2531994) | 1555.21 (1330.09-1813.67) | -2.61 (-2.77--2.45) |
| Commonwealth Middle Income | 15877525 (12833669-19042318) | 3433.93 (2775.61-4118.39) | 12821155 (10364724-15213308) | 2052.72 (1659.43-2435.71) | -1.63 (-1.72--1.54) |
| East Asia | 3247614 (2607591-3996290) | 984.62 (790.58-1211.61) | 498310 (356100-658128) | 186.39 (133.2-246.17) | -5.85 (-6.15--5.55) |
| East Asia & Pacific - WB | 8240522 (6789267-9849689) | 1533.04 (1263.05-1832.4) | 3071142 (2451253-3804789) | 655.64 (523.3-812.26) | -2.66 (-2.82--2.5) |
| Eastern Africa | 3073787 (2672218-3490112) | 3720.27 (3234.24-4224.15) | 2960543 (2503234-3404396) | 1866.9 (1578.52-2146.79) | -2.19 (-2.48--1.9) |
| Eastern Europe | 527682 (411361-651391) | 1025.38 (799.35-1265.77) | 142297 (96617-194307) | 401.47 (272.59-548.21) | -2.22 (-2.59--1.85) |
| Eastern Mediterranean Region | 4266659 (3612030-4965082) | 2625.52 (2222.69-3055.3) | 3078076 (2490624-3777355) | 1243.6 (1006.26-1526.12) | -1.94 (-2.22--1.65) |
| Eastern Sub-Saharan Africa | 3395774 (2954087-3840489) | 3749.31 (3261.64-4240.32) | 3226021 (2735196-3740764) | 1807.99 (1532.91-2096.47) | -2.34 (-2.63--2.06) |
| Europe | 1581137 (1214874-1984116) | 902.26 (693.25-1132.21) | 813724 (579155-1054824) | 579 (412.09-750.55) | -0.85 (-1.35--0.35) |
| Europe & Central Asia - WB | 1830080 (1443219-2254194) | 943.92 (744.38-1162.67) | 875059 (628380-1128281) | 536.69 (385.4-691.99) | -1.25 (-1.71--0.78) |
| European Region | 1847072 (1456133-2275651) | 942.32 (742.87-1160.96) | 890361 (639030-1147272) | 536.18 (384.83-690.9) | -1.24 (-1.71--0.77) |
| High-income Asia Pacific | 290900 (203681-390581) | 826.43 (578.65-1109.62) | 238809 (172734-310491) | 1064.9 (770.26-1384.54) | 1.35 (1.01-1.68) |
| High-income North America | 191982 (133848-271467) | 311.27 (217.01-440.14) | 16262 (12618-20397) | 24.78 (19.23-31.08) | -7.59 (-8.44--6.74) |
| Latin America & Caribbean - WB | 2794526 (2336587-3285374) | 1759.92 (1471.52-2069.04) | 779404 (600072-983583) | 495.25 (381.3-624.99) | -4.35 (-4.55--4.14) |
| Limited Health System | 22011647 (18360884-25839729) | 3415.07 (2848.66-4008.99) | 17313489 (14218498-20338476) | 1899.07 (1559.59-2230.88) | -1.81 (-1.95--1.68) |
| Middle East & North Africa - WB | 2179240 (1875216-2501554) | 2008.26 (1728.09-2305.28) | 1139851 (856118-1486482) | 832.34 (625.15-1085.45) | -2.08 (-2.41--1.74) |
| Minimal Health System | 2129030 (1894371-2370414) | 3353.95 (2984.28-3734.21) | 2374342 (2067121-2721630) | 1561.25 (1359.23-1789.61) | -2.44 (-2.78--2.09) |
| North Africa and Middle East | 2823187 (2440292-3214939) | 2009.59 (1737.04-2288.45) | 1648529 (1267669-2096473) | 899.25 (691.5-1143.6) | -1.99 (-2.35--1.64) |
| North America | 192046 (133900-271547) | 311.39 (217.11-440.29) | 16359 (12684-20525) | 24.93 (19.33-31.28) | -7.58 (-8.42--6.73) |
| Northern Africa | 1023085 (912060-1137234) | 2108.02 (1879.26-2343.22) | 625573 (477297-803435) | 946.99 (722.53-1216.24) | -2.23 (-2.42--2.04) |
| Oceania | 57105 (50742-64683) | 2130.88 (1893.45-2413.65) | 81586 (71211-93415) | 1605.74 (1401.54-1838.56) | -1.18 (-1.31--1.05) |
| Region of the Americas | 2977272 (2474254-3542011) | 1356.87 (1127.63-1614.25) | 791879 (610848-998844) | 355.86 (274.51-448.87) | -4.53 (-4.74--4.32) |
| South-East Asia Region | 15921867 (13054200-18984655) | 3213.86 (2635.02-3832.09) | 10531248 (8539834-12487144) | 2006.92 (1627.42-2379.65) | -1.52 (-1.59--1.45) |
| South Asia | 14722995 (12007398-17672221) | 3397.39 (2770.76-4077.94) | 10668801 (8549986-12702666) | 2104.21 (1686.32-2505.35) | -1.5 (-1.56--1.45) |
| South Asia - WB | 14882889 (12142033-17853493) | 3357.23 (2738.96-4027.33) | 10833122 (8685543-12901281) | 2057.86 (1649.91-2450.73) | -1.53 (-1.59--1.48) |
| Southeast Asia | 4732876 (3929006-5512759) | 2771.86 (2301.06-3228.6) | 2317454 (1875052-2821813) | 1342.26 (1086.02-1634.38) | -2.34 (-2.45--2.23) |
| Southern Africa | 1500877 (1304511-1683318) | 3760.42 (3268.43-4217.53) | 1068884 (909422-1252702) | 1527.57 (1299.68-1790.27) | -3.05 (-3.34--2.75) |
| Southern Latin America | 180110 (136939-224784) | 1206.65 (917.43-1505.95) | 48742 (34107-64974) | 336.26 (235.29-448.23) | -3.15 (-4.25--2.03) |
| Southern Sub-Saharan Africa | 811203 (672231-943407) | 3920.88 (3249.17-4559.88) | 343939 (283216-414994) | 1429.16 (1176.84-1724.41) | -3.26 (-3.49--3.02) |
| Sub-Saharan Africa - WB | 8791706 (7634524-9875388) | 3766.1 (3270.4-4230.32) | 8460230 (7185660-9787426) | 1718.89 (1459.93-1988.54) | -2.5 (-2.78--2.22) |
| Tropical Latin America | 877436 (705104-1059494) | 1636.58 (1315.15-1976.15) | 230390 (174060-296931) | 459.01 (346.78-591.58) | -4.89 (-5.2--4.59) |
| Western Africa | 3182675 (2727179-3615758) | 4024.25 (3448.31-4571.84) | 3217685 (2708164-3775145) | 1690.42 (1422.74-1983.28) | -2.79 (-3.02--2.56) |
| Western Europe | 635028 (448589-845962) | 894.18 (631.65-1191.19) | 527929 (376514-691130) | 775.02 (552.74-1014.6) | -0.07 (-0.66-0.52) |
| Western Pacific Region | 5288261 (4294014-6389996) | 1240.5 (1007.27-1498.94) | 1704865 (1311017-2144359) | 463.97 (356.79-583.57) | -3.27 (-3.4--3.15) |
| Western Sub-Saharan Africa | 3501216 (3006608-3976213) | 3984.1 (3421.27-4524.6) | 3678779 (3103324-4280308) | 1712.94 (1444.99-1993.03) | -2.7 (-2.94--2.46) |
| World Bank High Income | 1387918 (1005765-1845212) | 636.55 (461.28-846.28) | 960336 (696940-1242432) | 485.84 (352.59-628.56) | -0.31 (-0.71-0.1) |
| World Bank Low Income | 4758261 (4201462-5298763) | 3346.05 (2954.51-3726.14) | 4806463 (4133959-5505595) | 1665.51 (1432.48-1907.77) | -2.11 (-2.46--1.75) |
| World Bank Lower Middle Income | 24868658 (20606520-29386903) | 3145.62 (2606.51-3717.13) | 17421038 (14292676-20548820) | 1720.15 (1411.25-2028.98) | -1.87 (-1.98--1.76) |
| World Bank Upper Middle Income | 7899626 (6510742-9439978) | 1346.13 (1109.46-1608.61) | 1987786 (1475834-2570972) | 388.75 (288.62-502.8) | -4.06 (-4.26--3.86) |
| Country |  |  |  |  |  |
| Afghanistan | 64868 (54994-76794) | 1505.73 (1276.54-1782.56) | 101800 (80462-129621) | 716.87 (566.61-912.78) | -2.34 (-2.83--1.85) |
| Albania | 3159 (2614-3777) | 282.78 (233.99-338.04) | 452 (333-588) | 101.81 (75.09-132.52) | -3.11 (-3.62--2.59) |
| Algeria | 173542 (142450-208432) | 1618.16 (1328.25-1943.49) | 104940 (73248-145076) | 788.92 (550.67-1090.65) | -2.25 (-2.34--2.16) |
| American Samoa | 193 (145-250) | 1017.14 (762.18-1315.16) | 204 (142-274) | 1437.16 (1001.89-1935.11) | 1.05 (0.98-1.12) |
| Andorra | 86 (62-118) | 901.15 (652.63-1246.14) | 95 (64-132) | 936.71 (629.08-1294.16) | 0.41 (-0.16-0.98) |
| Angola | 183931 (166033-206627) | 3901.15 (3521.53-4382.53) | 277772 (238435-322339) | 1822 (1563.97-2114.33) | -2.73 (-3.05--2.41) |
| Antigua and Barbuda | 140 (100-189) | 771.27 (547.58-1040.09) | 162 (106-233) | 957.8 (628-1380.94) | 0.69 (0.52-0.87) |
| Argentina | 143214 (107977-180927) | 1413.03 (1065.36-1785.13) | 38091 (25782-52195) | 374.05 (253.18-512.55) | -2.97 (-4.14--1.79) |
| Armenia | 14141 (12143-16746) | 1355.5 (1163.97-1605.25) | 1489 (1063-1954) | 251.36 (179.53-329.92) | -5.17 (-5.96--4.37) |
| Australia | 11681 (8824-15286) | 308.55 (233.09-403.77) | 3666 (2599-4961) | 77.19 (54.71-104.45) | -3.71 (-4.24--3.18) |
| Austria | 24469 (18572-30477) | 1814.7 (1377.42-2260.33) | 9528 (7397-11821) | 734.6 (570.33-911.4) | -3.37 (-4.38--2.36) |
| Azerbaijan | 26790 (23571-30314) | 1103.84 (971.21-1249.05) | 9558 (6823-12694) | 404.91 (289.06-537.73) | -3.33 (-3.53--3.13) |
| Bahamas | 527 (371-728) | 653.31 (460.1-902.2) | 456 (292-684) | 562.28 (360.27-842.12) | 0.21 (-0.33-0.75) |
| Bahrain | 1172 (841-1562) | 717.7 (515.41-956.89) | 1585 (1072-2205) | 534.34 (361.19-743.05) | -1.31 (-1.75--0.87) |
| Bangladesh | 1776205 (1572015-2021204) | 3631.47 (3214-4132.37) | 598821 (510389-703037) | 1308.44 (1115.22-1536.16) | -3 (-3.09--2.9) |
| Barbados | 516 (359-695) | 827.69 (575.32-1114.28) | 571 (368-826) | 1211.91 (781.98-1754.19) | 1.26 (1.06-1.46) |
| Belarus | 17271 (13743-21055) | 718.45 (571.72-875.89) | 7657 (5435-10142) | 485.16 (344.4-642.63) | -0.88 (-1.02--0.74) |
| Belgium | 15071 (10606-20510) | 834.44 (587.22-1135.59) | 9936 (7119-13274) | 519.63 (372.33-694.23) | -1.48 (-2.19--0.76) |
| Belize | 827 (683-1003) | 1010.12 (833.98-1225) | 948 (640-1327) | 770.06 (519.91-1077.64) | -0.49 (-0.75--0.22) |
| Benin | 78477 (67932-89890) | 3240.47 (2805.06-3711.72) | 93895 (78316-110942) | 1544.23 (1288.01-1824.59) | -2.44 (-2.58--2.29) |
| Bermuda | 94 (66-124) | 788.81 (550.82-1036.88) | 105 (68-150) | 1242.42 (804.56-1772.64) | 1.31 (1.12-1.51) |
| Bhutan | 4655 (3836-5631) | 1775.4 (1463.13-2147.76) | 1869 (1548-2318) | 998.81 (826.83-1238.29) | -2.42 (-2.79--2.04) |
| Bolivia (Plurinational State of) | 62313 (54769-69800) | 2320.04 (2039.18-2598.8) | 12540 (9832-15694) | 359.66 (282.01-450.13) | -6.49 (-7.16--5.81) |
| Bosnia and Herzegovina | 2951 (2307-3682) | 269.33 (210.57-336.07) | 709 (527-935) | 144.58 (107.52-190.7) | -1.86 (-2.35--1.38) |
| Botswana | 22624 (19537-25732) | 3831.72 (3308.89-4358.02) | 12459 (10468-14741) | 1784.25 (1499.15-2111.01) | -2.54 (-2.71--2.37) |
| Brazil | 815015 (647170-993915) | 1569.01 (1245.89-1913.42) | 224413 (169711-289480) | 465.73 (352.2-600.76) | -4.71 (-5.02--4.4) |
| Brunei Darussalam | 196 (136-267) | 216.89 (149.88-294.88) | 145 (96-199) | 153.02 (101.57-210.57) | -1.16 (-1.57--0.75) |
| Bulgaria | 2799 (2277-3417) | 161.21 (131.17-196.81) | 740 (581-920) | 75.82 (59.54-94.23) | -1.61 (-1.92--1.3) |
| Burkina Faso | 170322 (152378-188428) | 3609.23 (3229-3992.91) | 153396 (130844-178750) | 1478.92 (1261.49-1723.36) | -3.05 (-3.52--2.58) |
| Burundi | 92656 (80626-106622) | 3534.98 (3075.99-4067.78) | 112654 (95011-132879) | 1924.35 (1622.97-2269.82) | -1.65 (-2.15--1.16) |
| Cabo Verde | 6033 (5418-6752) | 3834.54 (3443.93-4292.03) | 1778 (1252-2491) | 1241.4 (874.51-1739.85) | -5.03 (-5.82--4.23) |
| Cambodia | 125557 (112284-141731) | 2693.81 (2409.04-3040.83) | 55378 (45461-67119) | 1082.33 (888.5-1311.8) | -3.26 (-3.63--2.9) |
| Cameroon | 170269 (149441-191393) | 3487.66 (3061.03-3920.35) | 209791 (181928-245117) | 1557.83 (1350.93-1820.14) | -2.48 (-2.93--2.02) |
| Canada | 30685 (21461-42397) | 533.49 (373.12-737.13) | 7147 (4997-9659) | 115.81 (80.97-156.5) | -4.54 (-5.44--3.64) |
| Central African Republic | 40118 (36028-44305) | 3281.21 (2946.69-3623.65) | 37892 (33182-43074) | 1659.13 (1452.94-1886.04) | -2.2 (-2.49--1.91) |
| Chad | 116275 (99805-133155) | 3973.37 (3410.53-4550.18) | 208168 (179903-236049) | 2309.15 (1995.62-2618.43) | -1.46 (-1.7--1.22) |
| Chile | 30694 (23210-39668) | 772.78 (584.35-998.72) | 9001 (7015-11240) | 246.47 (192.1-307.79) | -4.44 (-5.4--3.46) |
| China | 3170555 (2539772-3901012) | 995.85 (797.73-1225.28) | 384970 (275278-514660) | 148.28 (106.03-198.23) | -6.87 (-7.3--6.43) |
| Colombia | 153666 (131203-178781) | 1317.55 (1124.95-1532.89) | 53324 (38786-70755) | 502.44 (365.46-666.68) | -3.71 (-4.16--3.26) |
| Comoros | 7655 (6563-8875) | 3598.98 (3085.48-4172.88) | 4381 (3678-5206) | 1824.13 (1531.61-2167.68) | -2.39 (-2.74--2.02) |
| Congo | 34540 (30343-39084) | 3280.23 (2881.65-3711.8) | 34170 (28638-40666) | 1771.15 (1484.39-2107.86) | -1.8 (-2.22--1.37) |
| Cook Islands | 73 (51-99) | 1111.36 (774.63-1506.56) | 73 (50-103) | 1933.49 (1314.98-2728.32) | 1.74 (1.7-1.78) |
| Costa Rica | 8968 (7191-11111) | 797.71 (639.63-988.36) | 10131 (6728-13856) | 995.84 (661.38-1362.04) | 1.74 (1.39-2.09) |
| Croatia | 866 (675-1080) | 87.7 (68.42-109.4) | 366 (289-459) | 61.36 (48.44-76.87) | -0.6 (-0.77--0.42) |
| Cuba | 16914 (12104-22481) | 675.49 (483.4-897.78) | 17522 (11396-25150) | 986.02 (641.32-1415.3) | 1.36 (1.13-1.58) |
| Cyprus | 1831 (1300-2536) | 924.88 (656.68-1281.08) | 2306 (1579-3123) | 1054.19 (721.98-1428) | 1.02 (0.53-1.51) |
| Czechia | 1863 (1357-2466) | 84.56 (61.58-111.9) | 1353 (1039-1736) | 78.83 (60.52-101.14) | 0.22 (-0.17-0.61) |
| C?te d'Ivoire | 171258 (146537-196969) | 3002.79 (2569.34-3453.59) | 173640 (148629-204791) | 1500.54 (1284.41-1769.74) | -2.3 (-2.55--2.05) |
| Democratic People's Republic of Korea | 46227 (38083-55402) | 777.02 (640.12-931.25) | 74409 (52878-102328) | 1558.73 (1107.69-2143.57) | 3.36 (2.74-3.98) |
| Democratic Republic of the Congo | 536580 (476426-599959) | 3030.88 (2691.1-3388.88) | 556674 (477007-654648) | 1464.97 (1255.32-1722.81) | -2.17 (-2.62--1.72) |
| Denmark | 7590 (5436-10254) | 859.26 (615.4-1160.81) | 9599 (6706-12851) | 1006.06 (702.86-1346.82) | 0.9 (0.39-1.42) |
| Djibouti | 6269 (5407-7195) | 3600.72 (3105.63-4132.22) | 6801 (5566-8272) | 1646.1 (1347.09-2002.03) | -2.89 (-3.03--2.75) |
| Dominica | 161 (116-215) | 647.3 (467.26-866.45) | 112 (72-165) | 821.6 (524.04-1203.48) | 0.73 (0.5-0.96) |
| Dominican Republic | 49111 (43495-55476) | 1822 (1613.65-2058.15) | 18596 (14256-24381) | 632.85 (485.15-829.72) | -3.21 (-3.72--2.7) |
| Ecuador | 157899 (139494-176922) | 4085.05 (3608.89-4577.21) | 17568 (14162-21611) | 346.45 (279.28-426.18) | -8.88 (-9.54--8.22) |
| Egypt | 525494 (477024-580691) | 2369.04 (2150.53-2617.88) | 383755 (297302-489537) | 1041.25 (806.68-1328.27) | -2.12 (-2.35--1.9) |
| El Salvador | 38523 (34176-44186) | 1785.03 (1583.63-2047.47) | 8580 (6271-11341) | 471.76 (344.77-623.56) | -5.17 (-5.78--4.55) |
| Equatorial Guinea | 6955 (6216-7819) | 3531.73 (3156.81-3970.86) | 8436 (6820-10317) | 1442.08 (1165.88-1763.65) | -3.33 (-3.69--2.97) |
| Eritrea | 67670 (58581-76334) | 4250.93 (3679.96-4795.19) | 58713 (50151-67735) | 2325.6 (1986.48-2682.98) | -2.01 (-2.2--1.82) |
| Estonia | 2256 (1683-2857) | 646.31 (482-818.47) | 1295 (929-1715) | 599.38 (429.7-793.39) | 0.47 (0.02-0.91) |
| Eswatini | 15627 (13242-18055) | 4051.21 (3432.98-4680.69) | 8834 (7449-10380) | 2140.8 (1805.17-2515.45) | -2.14 (-2.3--1.98) |
| Ethiopia | 888238 (721041-1069870) | 3645.72 (2959.46-4391.21) | 769924 (616261-949240) | 1736 (1389.53-2140.32) | -2.44 (-2.72--2.15) |
| Fiji | 4623 (3863-5461) | 1642.63 (1372.74-1940.43) | 3637 (3054-4320) | 1334.4 (1120.75-1585.25) | -0.62 (-0.76--0.48) |
| Finland | 8843 (6323-12015) | 916.39 (655.22-1245.19) | 4652 (3305-6430) | 549.23 (390.11-759.03) | -1.52 (-2.36--0.69) |
| France | 112567 (80061-149492) | 960.94 (683.45-1276.16) | 186246 (124773-252081) | 1604.52 (1074.92-2171.68) | 2.02 (1.66-2.38) |
| Gabon | 11362 (9677-13126) | 2788.28 (2374.61-3221.06) | 9041 (7279-10972) | 1414.6 (1138.85-1716.69) | -2.11 (-2.35--1.87) |
| Gambia | 12023 (10448-13723) | 2606.4 (2264.94-2974.96) | 10739 (8916-12860) | 1080.97 (897.5-1294.46) | -3.13 (-3.71--2.54) |
| Georgia | 14763 (11856-18044) | 1078.63 (866.19-1318.32) | 2686 (2096-3332) | 365.01 (284.83-452.82) | -2.47 (-3.23--1.7) |
| Germany | 137366 (94728-190543) | 1061.08 (731.73-1471.84) | 58381 (46636-71871) | 487.94 (389.79-600.7) | -1.79 (-2.84--0.74) |
| Ghana | 236838 (211626-263744) | 3526.17 (3150.8-3926.76) | 167536 (142110-196721) | 1300.44 (1103.07-1526.97) | -3.4 (-3.67--3.13) |
| Greece | 19313 (13606-26207) | 954.41 (672.35-1295.09) | 10792 (7364-14843) | 773.77 (528.01-1064.19) | 0.1 (-0.59-0.78) |
| Greenland | 26 (19-35) | 185.33 (132.99-246.88) | 8 (5-11) | 66.07 (45.39-90.32) | -3.45 (-3.88--3.02) |
| Grenada | 347 (246-469) | 1039.76 (736.02-1404.43) | 390 (243-574) | 1785.18 (1112.18-2630.86) | 1.62 (1.25-1.98) |
| Guam | 464 (340-612) | 1113.44 (815.98-1468.11) | 612 (425-825) | 1673.92 (1160.56-2254.73) | 1.42 (1.24-1.59) |
| Guatemala | 124777 (114439-136522) | 3072.39 (2817.85-3361.59) | 51285 (45315-57753) | 1039.42 (918.43-1170.51) | -3.93 (-4.19--3.67) |
| Guinea | 97707 (84169-112260) | 3550.6 (3058.65-4079.45) | 95289 (80168-114074) | 1576.12 (1326.01-1886.84) | -2.88 (-3.13--2.63) |
| Guinea-Bissau | 14872 (12727-17025) | 3083.03 (2638.34-3529.25) | 12506 (10327-14862) | 1392.47 (1149.83-1654.73) | -2.84 (-3.23--2.45) |
| Guyana | 2420 (2078-2830) | 823.21 (706.84-962.78) | 943 (741-1238) | 442.12 (347.29-580.13) | -2.45 (-2.87--2.02) |
| Haiti | 33781 (29653-37970) | 1245.11 (1092.97-1399.53) | 23258 (20135-27372) | 534.33 (462.58-628.83) | -1.3 (-2.37--0.22) |
| Honduras | 38439 (33916-43714) | 1739.75 (1535.07-1978.54) | 23524 (19909-28176) | 717.82 (607.52-859.78) | -3.36 (-3.71--3.01) |
| Hungary | 6787 (4823-9018) | 318.5 (226.3-423.16) | 1788 (1302-2323) | 128.8 (93.74-167.33) | -3.53 (-3.94--3.12) |
| Iceland | 596 (424-809) | 939.85 (668.24-1275.77) | 674 (469-910) | 998.61 (694.08-1347.86) | 0.6 (0.09-1.11) |
| India | 10878249 (8647339-13274291) | 3331.56 (2648.33-4065.37) | 8468849 (6721212-10092800) | 2311.37 (1834.4-2754.59) | -1.23 (-1.31--1.15) |
| Indonesia | 2143016 (1754203-2558318) | 3163.72 (2589.72-3776.83) | 959264 (798710-1146594) | 1425.6 (1187-1704) | -2.45 (-2.6--2.29) |
| Iran (Islamic Republic of) | 543571 (427439-669739) | 2141.35 (1683.86-2638.37) | 128107 (85798-180179) | 634.84 (425.18-892.89) | -3.83 (-4.04--3.61) |
| Iraq | 159730 (135416-187305) | 1939.31 (1644.11-2274.09) | 107623 (79101-141440) | 799.46 (587.59-1050.67) | -2.76 (-3.08--2.45) |
| Ireland | 9090 (6316-12677) | 925.22 (642.84-1290.26) | 9640 (6499-13095) | 966.7 (651.72-1313.15) | 2.37 (1.32-3.42) |
| Israel | 12748 (8835-17351) | 831.56 (576.29-1131.8) | 14225 (10068-19030) | 541.34 (383.13-724.19) | -1.03 (-1.89--0.17) |
| Italy | 52057 (35276-72209) | 564.02 (382.2-782.35) | 18917 (14177-24488) | 248.91 (186.54-322.21) | -2.33 (-2.85--1.8) |
| Jamaica | 10899 (9098-13132) | 1304.91 (1089.33-1572.29) | 3728 (2461-5378) | 638.44 (421.53-920.97) | -1.82 (-2.13--1.5) |
| Japan | 259389 (180645-350189) | 1123.43 (782.38-1516.69) | 226076 (163986-293819) | 1463.81 (1061.78-1902.43) | 1.38 (1.02-1.74) |
| Jordan | 17026 (13787-20674) | 1042.37 (844.07-1265.7) | 21284 (14973-28777) | 585.85 (412.12-792.08) | -1.1 (-1.71--0.48) |
| Kazakhstan | 50728 (43734-57495) | 976.25 (841.64-1106.47) | 14504 (10014-19393) | 267.28 (184.54-357.37) | -3.82 (-4.13--3.51) |
| Kenya | 392353 (319664-471646) | 3512.51 (2861.77-4222.38) | 407174 (328750-494410) | 2181.46 (1761.3-2648.83) | -1.42 (-1.57--1.27) |
| Kiribati | 680 (601-771) | 2301.02 (2036.14-2611.83) | 541 (461-623) | 1287.85 (1097.37-1483.28) | -2.15 (-2.3--2) |
| Kuwait | 6498 (4892-8201) | 1172.25 (882.54-1479.34) | 6296 (4200-8724) | 744.63 (496.8-1031.8) | -0.4 (-0.65--0.14) |
| Kyrgyzstan | 18584 (16453-21223) | 1107.79 (980.75-1265.12) | 5570 (4291-7153) | 244.91 (188.65-314.51) | -3.98 (-4.26--3.7) |
| Lao People's Democratic Republic | 54173 (49465-59795) | 2939.21 (2683.78-3244.26) | 31554 (27894-36544) | 1374.11 (1214.72-1591.39) | -2.41 (-2.51--2.31) |
| Latvia | 3703 (2789-4685) | 650.89 (490.12-823.38) | 1111 (803-1439) | 374.05 (270.35-484.5) | -0.95 (-1.34--0.56) |
| Lebanon | 10763 (8315-13823) | 1029.12 (795.05-1321.64) | 13878 (9513-19229) | 1085.9 (744.35-1504.56) | -0.28 (-0.57-0.01) |
| Lesotho | 28699 (24418-32871) | 4204.41 (3577.29-4815.65) | 13121 (10946-15360) | 2081.01 (1736.08-2436.2) | -2.43 (-2.51--2.35) |
| Liberia | 42829 (37869-47989) | 3789.57 (3350.76-4246.16) | 56966 (48297-64584) | 2606.01 (2209.45-2954.55) | -1.08 (-1.5--0.66) |
| Libya | 23495 (19260-28303) | 1297.39 (1063.54-1562.87) | 12472 (8340-17848) | 836.11 (559.09-1196.54) | -1.3 (-1.49--1.11) |
| Lithuania | 5458 (4029-6956) | 657.01 (485.05-837.41) | 2264 (1612-2965) | 555.2 (395.33-727.01) | -0.21 (-0.4--0.01) |
| Luxembourg | 558 (396-745) | 844.63 (599.59-1126.91) | 553 (395-741) | 546.14 (390.61-732.16) | -1.42 (-2.14--0.69) |
| Madagascar | 312956 (287347-335070) | 5736.03 (5266.66-6141.35) | 381470 (342083-417147) | 3251.11 (2915.43-3555.17) | -1.71 (-1.88--1.55) |
| Malawi | 178604 (158536-200474) | 3925.68 (3484.61-4406.38) | 152551 (128810-183174) | 1877.81 (1585.58-2254.77) | -2.42 (-2.84--2) |
| Malaysia | 67106 (52852-83829) | 1020.94 (804.08-1275.35) | 145839 (102019-195200) | 1915.5 (1339.94-2563.81) | 2.47 (2.23-2.71) |
| Maldives | 2549 (2227-2870) | 2426.96 (2120.06-2732.44) | 1538 (1184-1986) | 1535.46 (1181.83-1982.59) | -1.41 (-1.72--1.1) |
| Mali | 171519 (150450-191658) | 4153.2 (3643.02-4640.84) | 208136 (172804-247547) | 1798.03 (1492.8-2138.49) | -2.86 (-3.17--2.54) |
| Malta | 816 (561-1135) | 932.47 (640.82-1296.47) | 630 (424-862) | 983.54 (661.84-1346.85) | 0.62 (0.18-1.06) |
| Marshall Islands | 287 (244-339) | 1309.69 (1114.01-1545.89) | 162 (124-210) | 926.83 (709.72-1202.39) | -1.77 (-2.11--1.43) |
| Mauritania | 30314 (25715-34808) | 3279.5 (2781.95-3765.65) | 42448 (35659-49231) | 2290.74 (1924.37-2656.79) | -1.33 (-1.71--0.94) |
| Mauritius | 7416 (6243-8826) | 2246.97 (1891.63-2673.96) | 2442 (1769-3204) | 1177.34 (853.14-1544.6) | -1.21 (-1.79--0.63) |
| Mexico | 650714 (504673-822848) | 1947.3 (1510.26-2462.42) | 86854 (66803-108772) | 270.85 (208.32-339.2) | -6.45 (-6.78--6.11) |
| Micronesia (Federated States of) | 607 (515-711) | 1322.55 (1121.8-1547.44) | 277 (201-365) | 903.83 (657.06-1192.11) | -2.16 (-2.72--1.61) |
| Monaco | 32 (23-44) | 903.92 (644.52-1248.3) | 47 (32-65) | 953.89 (645.42-1311.68) | 0.53 (0.08-0.98) |
| Mongolia | 8397 (6974-10117) | 933.04 (774.91-1124.12) | 962 (796-1142) | 88.58 (73.22-105.09) | -6.45 (-7.67--5.22) |
| Montenegro | 356 (269-461) | 220.27 (166.31-285.1) | 130 (93-174) | 116.34 (83.45-155.85) | -1.07 (-1.69--0.45) |
| Morocco | 226657 (205775-247846) | 2316.19 (2102.8-2532.72) | 54253 (41623-70611) | 554.07 (425.07-721.12) | -4.74 (-5.19--4.28) |
| Mozambique | 197441 (178044-219351) | 3182.4 (2869.75-3535.55) | 164890 (138888-197130) | 1155.83 (973.56-1381.82) | -3.71 (-3.97--3.46) |
| Myanmar | 381325 (343944-424678) | 2580.65 (2327.67-2874.04) | 200780 (169253-237962) | 1285.83 (1083.93-1523.96) | -2.68 (-2.93--2.43) |
| Namibia | 20540 (17197-23851) | 3419.2 (2862.75-3970.3) | 17466 (14286-20747) | 2115.97 (1730.73-2513.47) | -1.37 (-1.56--1.17) |
| Nauru | 42 (34-53) | 1006.2 (801.58-1244.05) | 46 (34-60) | 1146.62 (859.49-1504.75) | 0.27 (0.2-0.34) |
| Nepal | 247010 (217247-279631) | 2931.81 (2578.55-3318.99) | 72770 (60122-88604) | 788.65 (651.57-960.24) | -3.55 (-3.88--3.22) |
| Netherlands | 32086 (22224-43513) | 1177.34 (815.47-1596.66) | 56299 (37508-76349) | 2099.12 (1398.49-2846.7) | 2.42 (1.78-3.06) |
| New Zealand | 5198 (3651-7165) | 649.66 (456.34-895.43) | 2447 (1850-3090) | 249.27 (188.46-314.74) | -2.72 (-3.29--2.15) |
| Nicaragua | 35822 (31265-40610) | 1966.95 (1716.74-2229.85) | 8943 (6629-12019) | 451.57 (334.72-606.91) | -5.81 (-6.31--5.3) |
| Niger | 173830 (155672-196375) | 4278.43 (3831.5-4833.3) | 236113 (204820-269089) | 1849.84 (1604.67-2108.19) | -2.77 (-3.23--2.32) |
| Nigeria | 1704173 (1399294-2000800) | 4355.76 (3576.51-5113.92) | 1747093 (1426990-2123972) | 1719.76 (1404.67-2090.75) | -2.92 (-3.1--2.74) |
| Niue | 9 (7-12) | 1132.97 (868.74-1469.16) | 4 (3-6) | 1081.74 (794.75-1433.95) | 0.33 (-0.08-0.74) |
| North Macedonia | 3454 (3073-3865) | 655.69 (583.4-733.71) | 456 (338-584) | 139.07 (103.29-178.35) | -4.23 (-4.64--3.81) |
| Northern Mariana Islands | 132 (97-171) | 1081.93 (798.36-1402.88) | 192 (133-267) | 1702.96 (1180.97-2370.81) | 1.33 (1.27-1.39) |
| Norway | 5587 (3925-7654) | 699.78 (491.68-958.75) | 3910 (2808-5230) | 423.28 (303.92-566.17) | -2.42 (-2.8--2.03) |
| Oman | 7837 (6466-9395) | 932.58 (769.43-1117.92) | 5495 (3958-7404) | 449.33 (323.67-605.41) | -1.66 (-2.21--1.1) |
| Pakistan | 1816876 (1483890-2170925) | 3689.68 (3013.46-4408.68) | 1526491 (1222833-1864317) | 1786.58 (1431.18-2181.97) | -2.03 (-2.24--1.83) |
| Palau | 58 (46-74) | 1284.14 (1010.24-1613.94) | 33 (24-45) | 1014.71 (741.44-1378.54) | -1.18 (-1.46--0.9) |
| Palestine | 8579 (6699-10742) | 886.04 (691.82-1109.39) | 7282 (5026-10131) | 390 (269.17-542.58) | -1.2 (-1.95--0.45) |
| Panama | 17601 (14856-20691) | 2110.69 (1781.44-2481.16) | 14740 (11446-18206) | 1277.97 (992.45-1578.53) | -1.88 (-2.25--1.51) |
| Papua New Guinea | 41624 (37115-46530) | 2448.4 (2183.13-2736.94) | 66777 (58529-76242) | 1704.77 (1494.21-1946.43) | -1.48 (-1.62--1.35) |
| Paraguay | 62421 (55267-69905) | 3738.74 (3310.25-4186.98) | 5976 (4361-7821) | 297.67 (217.23-389.57) | -9.02 (-9.47--8.57) |
| Peru | 259038 (231533-290497) | 3120.6 (2789.25-3499.59) | 59015 (44395-76957) | 618.78 (465.48-806.89) | -5.25 (-5.63--4.88) |
| Philippines | 939137 (768769-1107234) | 3724.61 (3048.94-4391.29) | 418779 (337706-504017) | 1231.76 (993.3-1482.47) | -4.01 (-4.25--3.76) |
| Poland | 12987 (9509-17683) | 135.61 (99.3-184.65) | 1236 (977-1564) | 21.01 (16.59-26.57) | -5.42 (-6.4--4.42) |
| Portugal | 13394 (9998-17469) | 633.01 (472.54-825.6) | 5738 (4454-7236) | 421.25 (326.97-531.21) | -1.01 (-1.34--0.68) |
| Puerto Rico | 8966 (6002-12516) | 900.38 (602.7-1256.96) | 3688 (2467-5357) | 829.86 (555.23-1205.5) | -0.04 (-0.47-0.4) |
| Qatar | 864 (651-1123) | 691.23 (520.97-897.96) | 2728 (1923-3634) | 552.35 (389.35-735.7) | -0.97 (-1.25--0.7) |
| Republic of Korea | 30908 (21091-41188) | 271.82 (185.49-362.23) | 12278 (8226-16698) | 202.12 (135.42-274.88) | -0.76 (-1.22--0.3) |
| Republic of Moldova | 11832 (10077-13896) | 957.36 (815.38-1124.36) | 1841 (1416-2321) | 352.5 (271.13-444.37) | -2.39 (-2.91--1.86) |
| Romania | 8980 (7713-10402) | 161.27 (138.52-186.81) | 2232 (1769-2769) | 74.16 (58.76-91.98) | -2.07 (-2.42--1.72) |
| Russian Federation | 404448 (314207-501846) | 1165.6 (905.53-1446.3) | 101628 (68902-138906) | 389.72 (264.22-532.67) | -2.53 (-3.01--2.04) |
| Rwanda | 120722 (107153-136269) | 3558.07 (3158.15-4016.28) | 72111 (60179-87186) | 1450.79 (1210.73-1754.1) | -3.01 (-3.5--2.53) |
| Saint Kitts and Nevis | 115 (94-140) | 814.29 (662.89-992.53) | 86 (62-116) | 872.12 (625.37-1173.69) | 0.19 (0.03-0.36) |
| Saint Lucia | 431 (328-556) | 835.37 (637.29-1079.67) | 431 (279-634) | 1452.77 (938.02-2133.75) | 1.87 (1.67-2.06) |
| Saint Vincent and the Grenadines | 391 (308-498) | 951.27 (749.85-1211.23) | 215 (141-308) | 862.62 (563.76-1233.33) | -0.28 (-0.39--0.17) |
| Samoa | 794 (614-1010) | 1114.75 (861.12-1417.53) | 1028 (738-1382) | 1285.69 (923.29-1728.33) | 0.61 (0.5-0.73) |
| San Marino | 34 (24-46) | 826.7 (588.46-1131.79) | 38 (26-52) | 870.38 (587.19-1183.55) | 0.57 (0.04-1.1) |
| Sao Tome and Principe | 1567 (1361-1817) | 2765.38 (2401.83-3207.34) | 645 (495-845) | 829.02 (635.56-1086.27) | -3.75 (-4.31--3.18) |
| Saudi Arabia | 88026 (72697-104686) | 1343.22 (1109.31-1597.43) | 62990 (42942-86943) | 832.62 (567.62-1149.23) | -0.78 (-1.26--0.31) |
| Senegal | 151726 (135529-168534) | 4155.43 (3711.82-4615.75) | 121122 (103529-140185) | 1904.26 (1627.67-2203.98) | -2.47 (-2.82--2.13) |
| Serbia | 4150 (2954-5491) | 191.35 (136.18-253.2) | 1647 (1168-2154) | 124.02 (87.94-162.25) | -0.18 (-1.09-0.75) |
| Seychelles | 230 (182-290) | 969.78 (766.11-1220.84) | 150 (109-201) | 640.3 (465.48-858.31) | -0.4 (-0.81-0.02) |
| Sierra Leone | 76357 (66982-86054) | 4212.49 (3695.29-4747.46) | 73514 (61927-85999) | 2055.65 (1731.65-2404.76) | -2.61 (-2.83--2.4) |
| Singapore | 407 (323-499) | 62.68 (49.72-76.77) | 311 (236-405) | 38.25 (29.09-49.93) | -1.27 (-1.57--0.97) |
| Slovakia | 834 (618-1094) | 62.87 (46.62-82.49) | 435 (332-556) | 50.77 (38.79-64.94) | -0.08 (-0.28-0.13) |
| Slovenia | 615 (446-794) | 148.69 (107.97-192.09) | 473 (351-616) | 151.38 (112.29-197.24) | 0.87 (0.46-1.29) |
| Solomon Islands | 2245 (1931-2620) | 1441.58 (1240.38-1682.55) | 2404 (1990-2881) | 924.45 (765.26-1107.89) | -1.54 (-1.61--1.48) |
| Somalia | 121590 (104978-138810) | 3121.16 (2694.75-3563.19) | 135162 (116216-158891) | 1308.51 (1125.1-1538.23) | -2.69 (-3.15--2.23) |
| South Africa | 617279 (505072-731703) | 4534.32 (3710.08-5374.83) | 225852 (179278-285130) | 1485.36 (1179.06-1875.22) | -3.64 (-3.88--3.39) |
| South Sudan | 108748 (94229-124439) | 4144.1 (3590.84-4742.06) | 125540 (107812-143748) | 2922.97 (2510.19-3346.91) | -1.02 (-1.25--0.79) |
| Spain | 63267 (44520-85301) | 807.38 (568.15-1088.57) | 47872 (35687-61052) | 738.68 (550.66-942.05) | 0.12 (-0.27-0.51) |
| Sri Lanka | 92476 (78145-108453) | 1671.34 (1412.33-1960.1) | 60984 (43659-82097) | 1194.82 (855.39-1608.48) | -1.3 (-1.4--1.21) |
| Sudan | 271192 (244213-303533) | 3049.69 (2746.3-3413.39) | 294567 (246826-345404) | 1775.65 (1487.87-2082.09) | -1.82 (-2.37--1.27) |
| Suriname | 1409 (1184-1662) | 1081.92 (909.29-1275.91) | 997 (728-1348) | 695.83 (507.98-941) | -1.42 (-1.59--1.24) |
| Sweden | 9629 (6849-13183) | 623.45 (443.44-853.58) | 8846 (6362-12056) | 485.87 (349.41-662.15) | -0.25 (-0.56-0.06) |
| Switzerland | 12739 (9644-16653) | 1102.32 (834.49-1440.99) | 15460 (11436-20250) | 1160.26 (858.24-1519.7) | 0.56 (0.15-0.97) |
| Syrian Arab Republic | 96351 (81602-113968) | 1626.97 (1377.92-1924.44) | 32594 (21976-44716) | 889.74 (599.89-1220.65) | -1.87 (-2.08--1.65) |
| Taiwan (Province of China) | 30832 (23982-38891) | 559.75 (435.4-706.07) | 38931 (27948-52760) | 1321.15 (948.45-1790.47) | 3.85 (3.46-4.24) |
| Tajikistan | 61012 (55214-67476) | 2627.53 (2377.83-2905.87) | 27062 (23306-31664) | 755.01 (650.23-883.4) | -4.32 (-4.53--4.1) |
| Thailand | 339629 (285577-398686) | 2014.58 (1693.96-2364.89) | 83644 (62773-109553) | 856.45 (642.76-1121.75) | -2.83 (-3.1--2.55) |
| Timor-Leste | 10526 (9279-11782) | 3164.5 (2789.49-3542.12) | 8319 (7012-9770) | 1597.93 (1346.83-1876.59) | -2.09 (-2.29--1.88) |
| Togo | 74711 (65730-84674) | 4238.82 (3729.31-4804.13) | 65961 (56140-77129) | 1993.37 (1696.55-2330.84) | -2.51 (-2.73--2.29) |
| Tokelau | 6 (5-8) | 1057.52 (833.75-1325.63) | 6 (4-8) | 1426.56 (1002.88-1953.96) | 0.85 (0.79-0.91) |
| Tonga | 443 (336-575) | 1060.14 (803.48-1375.43) | 518 (340-749) | 1328.65 (871.75-1918.95) | 0.99 (0.91-1.08) |
| Trinidad and Tobago | 2533 (1915-3242) | 623.48 (471.39-797.92) | 1732 (1142-2505) | 635.95 (419.37-919.47) | 0.23 (-0.02-0.49) |
| Tunisia | 43582 (35530-52535) | 1403.4 (1144.12-1691.67) | 27705 (19144-37754) | 1001.74 (692.19-1365.08) | -0.5 (-0.71--0.28) |
| Turkmenistan | 26566 (23894-29771) | 1770.08 (1592.01-1983.61) | 3816 (2840-4947) | 250.41 (186.38-324.61) | -6.89 (-7.21--6.57) |
| Tuvalu | 44 (37-51) | 1260.32 (1060.87-1479.92) | 40 (29-53) | 1070.64 (788.57-1410.66) | -0.41 (-0.57--0.26) |
| Türkiye | 326176 (270833-389627) | 1591.98 (1321.86-1901.67) | 132430 (90342-186697) | 715.05 (487.79-1008.06) | -2.19 (-2.6--1.78) |
| Uganda | 313530 (277494-353493) | 3723.8 (3295.81-4198.44) | 347616 (293936-413289) | 1752.48 (1481.85-2083.56) | -2.26 (-2.54--1.97) |
| Ukraine | 82714 (63807-103268) | 727.16 (560.94-907.86) | 26501 (17258-37706) | 417.65 (271.99-594.25) | -1.86 (-2.04--1.68) |
| United Arab Emirates | 5588 (4230-7066) | 948.04 (717.76-1198.91) | 7830 (5378-10627) | 584.83 (401.73-793.78) | -1.31 (-1.79--0.83) |
| United Kingdom | 94739 (66690-131515) | 867.53 (610.69-1204.29) | 53079 (36322-72602) | 450.47 (308.26-616.15) | -1.49 (-2.41--0.56) |
| United Republic of Tanzania | 455219 (402944-510556) | 3769.77 (3336.87-4228.03) | 354493 (292932-426811) | 1452.67 (1200.4-1749.02) | -3.14 (-3.51--2.76) |
| United States of America | 161267 (111435-230190) | 288.44 (199.31-411.71) | 9106 (7352-11232) | 15.32 (12.37-18.9) | -10.18 (-11.64--8.7) |
| United States Virgin Islands | 240 (165-328) | 751.02 (515.46-1026.96) | 91 (58-132) | 678.01 (435.42-986.27) | -0.38 (-0.8-0.05) |
| Uruguay | 6193 (4981-7571) | 756.54 (608.5-924.89) | 1648 (1143-2191) | 249.86 (173.26-332.23) | -3.85 (-4.94--2.75) |
| Uzbekistan | 96322 (85305-108779) | 1125.83 (997.06-1271.44) | 11467 (8040-15266) | 113.64 (79.67-151.29) | -6.93 (-7.21--6.65) |
| Vanuatu | 1135 (968-1315) | 1667.03 (1421.39-1931.61) | 1370 (1148-1597) | 1175.62 (985.38-1370.17) | -1.33 (-1.56--1.09) |
| Venezuela (Bolivarian Republic of) | 59503 (51424-68358) | 838.79 (724.9-963.6) | 79844 (57359-107148) | 1205.38 (865.93-1617.59) | 0.67 (0.4-0.94) |
| Viet Nam | 562890 (455176-681719) | 2123.11 (1716.84-2571.31) | 345551 (261436-442755) | 1395.45 (1055.77-1788) | -0.8 (-1.06--0.55) |
| Yemen | 220630 (198714-245091) | 3109.93 (2801.01-3454.73) | 137376 (113036-165120) | 996.31 (819.78-1197.52) | -1.33 (-2.36--0.29) |
| Zambia | 129697 (116810-143587) | 3454.42 (3111.17-3824.37) | 129732 (110288-153091) | 1568.42 (1333.34-1850.81) | -2.83 (-3.33--2.32) |
| Zimbabwe | 106433 (91139-124091) | 2209.85 (1892.29-2576.48) | 66208 (56355-77909) | 1051.95 (895.4-1237.86) | -2.14 (-2.42--1.86) |

eTable 2. Mortality of diarrhea disease in children at the global, sex, age-group levels, regional and national levels from 1990 to 2021.

|  | Rate per 100 000(95% UI) | |  | |  |
| --- | --- | --- | --- | --- | --- |
|  | 1990 | | 2021 | | 1990-2021 |
|  | Deaths cases | Deaths rate | Deaths cases | Deaths rate | EAPC |
| Global | 1739616 (1373131-2048271) | 100.03 (78.95-117.77) | 374246 (281541-498684) | 18.6 (13.99-24.79) | -5 (-5.3--4.69) |
| Sex |  |  |  |  |  |
| Female | 821547 (654001-975494) | 97.15 (77.34-115.35) | 170860 (128150-227361) | 17.55 (13.16-23.35) | -5.08 (-5.43--4.72) |
| Male | 918070 (678676-1141561) | 102.75 (75.96-127.76) | 203386 (142571-286434) | 19.59 (13.73-27.59) | -4.93 (-5.18--4.67) |
| Age |  |  |  |  |  |
| <5 years | 1636314 (1285402-1930943) | 263.95 (207.34-311.47) | 340429 (250952-464258) | 51.72 (38.13-70.54) | NA |
| 5-9 years | 70289 (40220-99719) | 12.05 (6.89-17.09) | 20943 (11638-34889) | 3.05 (1.69-5.08) | NA |
| 10-14 years | 33013 (20546-50102) | 6.16 (3.84-9.35) | 12873 (7498-21218) | 1.93 (1.12-3.18) | NA |
| SDI region |  |  |  |  |  |
| High-middle SDI | 34407 (27556-41287) | 12.57 (10.07-15.09) | 1873 (1456-2295) | 0.81 (0.63-0.99) | -8.29 (-8.45--8.13) |
| High SDI | 2207 (1723-2936) | 1.19 (0.93-1.58) | 475 (411-539) | 0.28 (0.24-0.31) | -3.99 (-4.22--3.76) |
| Low-middle SDI | 794757 (647654-931121) | 168.34 (137.18-197.22) | 97622 (76595-126806) | 16.84 (13.21-21.87) | -6.93 (-7.23--6.64) |
| Low SDI | 602563 (450216-752458) | 263.23 (196.68-328.71) | 245329 (176547-335720) | 53.31 (38.36-72.95) | -4.96 (-5.29--4.63) |
| Middle SDI | 304639 (238428-360188) | 52.78 (41.31-62.4) | 28625 (21974-37114) | 5.05 (3.88-6.55) | -6.93 (-7.09--6.78) |
| GBD region |  |  |  |  |  |
| Advanced Health System | 9580 (7618-12885) | 3.3 (2.62-4.44) | 1012 (850-1186) | 0.4 (0.34-0.47) | -6.44 (-6.64--6.24) |
| Africa | 655089 (479794-806648) | 232.94 (170.61-286.83) | 269489 (191365-378328) | 48.4 (34.37-67.95) | -4.8 (-5.2--4.4) |
| African Region | 586647 (413088-735110) | 253.54 (178.53-317.71) | 253628 (178160-362014) | 53 (37.23-75.64) | -4.8 (-5.22--4.37) |
| America | 95632 (88322-103268) | 43.58 (40.25-47.06) | 8844 (6630-11306) | 3.97 (2.98-5.08) | -7.39 (-7.72--7.05) |
| Andean Latin America | 8212 (6923-9810) | 55.29 (46.61-66.05) | 632 (419-893) | 3.49 (2.31-4.93) | -8.95 (-9.23--8.67) |
| Asia | 978063 (788873-1156444) | 92.21 (74.38-109.03) | 94728 (70024-127719) | 8.69 (6.42-11.72) | -7 (-7.32--6.68) |
| Australasia | 15 (13-17) | 0.32 (0.29-0.36) | 7 (6-8) | 0.12 (0.1-0.14) | -0.38 (-1.63-0.89) |
| Basic Health System | 368235 (304836-427429) | 49.82 (41.24-57.83) | 27504 (21675-34821) | 3.96 (3.12-5.01) | -7.53 (-7.69--7.36) |
| Caribbean | 12716 (10416-15028) | 111.42 (91.27-131.68) | 4071 (2766-5663) | 35.38 (24.04-49.22) | -3.25 (-3.77--2.74) |
| Central Africa | 68482 (47539-87920) | 220.41 (153.01-282.97) | 38909 (24973-64174) | 54.15 (34.76-89.32) | -4.35 (-5--3.7) |
| Central Asia | 13834 (12480-15425) | 55.36 (49.94-61.72) | 1760 (1274-2384) | 6.36 (4.6-8.62) | -7.3 (-7.62--6.98) |
| Central Europe | 779 (695-875) | 2.64 (2.36-2.97) | 191 (157-224) | 1.08 (0.89-1.27) | -2.11 (-3.3--0.91) |
| Central Latin America | 42096 (38923-45864) | 65.39 (60.46-71.24) | 3239 (2407-4255) | 5.1 (3.79-6.7) | -7.75 (-8.2--7.29) |
| Central Sub-Saharan Africa | 62352 (43854-78493) | 246.46 (173.35-310.27) | 14838 (9240-22500) | 25.28 (15.75-38.34) | -6.85 (-7.83--5.86) |
| Commonwealth High Income | 81 (74-89) | 0.36 (0.32-0.39) | 45 (38-52) | 0.18 (0.15-0.21) | -0.51 (-1.73-0.71) |
| Commonwealth Low Income | 156475 (112472-197184) | 162.09 (116.51-204.26) | 31287 (21746-44638) | 22.41 (15.58-31.97) | -6.08 (-6.26--5.9) |
| Commonwealth Middle Income | 828010 (659657-990331) | 179.08 (142.67-214.18) | 183175 (138597-241638) | 29.33 (22.19-38.69) | -5.43 (-5.75--5.11) |
| East Asia | 75116 (56021-94299) | 22.77 (16.98-28.59) | 922 (712-1225) | 0.34 (0.27-0.46) | -13.15 (-13.44--12.86) |
| East Asia & Pacific - WB | 256098 (177722-321273) | 47.64 (33.06-59.77) | 17721 (13503-22649) | 3.78 (2.88-4.84) | -7.68 (-7.81--7.55) |
| Eastern Africa | 181739 (127184-239041) | 219.96 (153.93-289.32) | 64440 (45354-89124) | 40.64 (28.6-56.2) | -5.36 (-5.52--5.19) |
| Eastern Europe | 1435 (1362-1513) | 2.79 (2.65-2.94) | 98 (88-108) | 0.28 (0.25-0.31) | -7.36 (-7.74--6.97) |
| Eastern Mediterranean Region | 180750 (150204-216602) | 111.23 (92.43-133.29) | 39721 (28625-55489) | 16.05 (11.57-22.42) | -5.61 (-5.85--5.38) |
| Eastern Sub-Saharan Africa | 214097 (138815-289873) | 236.39 (153.27-320.05) | 74830 (53024-105219) | 41.94 (29.72-58.97) | -5.47 (-5.66--5.27) |
| Europe | 9771 (7851-13098) | 5.58 (4.48-7.47) | 859 (702-1063) | 0.61 (0.5-0.76) | -7.02 (-7.25--6.79) |
| Europe & Central Asia - WB | 21182 (18623-25370) | 10.93 (9.61-13.09) | 2408 (1873-3086) | 1.48 (1.15-1.89) | -6.84 (-7.13--6.55) |
| European Region | 21318 (18741-25518) | 10.88 (9.56-13.02) | 2426 (1892-3106) | 1.46 (1.14-1.87) | -6.85 (-7.14--6.55) |
| High-income Asia Pacific | 198 (163-245) | 0.56 (0.46-0.7) | 50 (43-56) | 0.22 (0.19-0.25) | -2.18 (-2.53--1.84) |
| High-income North America | 228 (215-242) | 0.37 (0.35-0.39) | 127 (111-143) | 0.19 (0.17-0.22) | -2.01 (-2.55--1.48) |
| Latin America & Caribbean - WB | 95420 (88106-103057) | 60.09 (55.49-64.9) | 8719 (6516-11171) | 5.54 (4.14-7.1) | -7.35 (-7.69--7.01) |
| Limited Health System | 1181782 (932015-1401109) | 183.35 (144.6-217.38) | 255253 (192644-341452) | 28 (21.13-37.45) | -5.7 (-5.98--5.42) |
| Middle East & North Africa - WB | 64056 (50242-79004) | 59.03 (46.3-72.81) | 6134 (4261-8358) | 4.48 (3.11-6.1) | -7.49 (-7.65--7.33) |
| Minimal Health System | 178976 (125594-223639) | 281.95 (197.85-352.31) | 90155 (62920-130726) | 59.28 (41.37-85.96) | -5 (-5.41--4.59) |
| North Africa and Middle East | 91953 (69092-117564) | 65.45 (49.18-83.68) | 12021 (8528-18091) | 6.56 (4.65-9.87) | -6.98 (-7.23--6.74) |
| North America | 228 (215-242) | 0.37 (0.35-0.39) | 127 (111-143) | 0.19 (0.17-0.22) | -2.02 (-2.55--1.48) |
| Northern Africa | 42245 (32666-54348) | 87.04 (67.31-111.98) | 4136 (3009-5698) | 6.26 (4.56-8.62) | -7.52 (-7.74--7.31) |
| Oceania | 1981 (1393-2899) | 73.92 (51.99-108.18) | 1832 (1119-2823) | 36.05 (22.02-55.56) | -1.71 (-1.95--1.47) |
| Region of the Americas | 95632 (88322-103268) | 43.58 (40.25-47.06) | 8844 (6630-11306) | 3.97 (2.98-5.08) | -7.39 (-7.72--7.05) |
| South-East Asia Region | 744893 (592129-900121) | 150.36 (119.52-181.69) | 62920 (43623-87013) | 11.99 (8.31-16.58) | -7.71 (-7.99--7.43) |
| South Asia | 679060 (551103-817209) | 156.7 (127.17-188.57) | 69140 (46159-95753) | 13.64 (9.1-18.89) | -7.32 (-7.65--6.99) |
| South Asia - WB | 687562 (557060-827341) | 155.1 (125.66-186.63) | 73184 (50632-100870) | 13.9 (9.62-19.16) | -7.26 (-7.58--6.93) |
| Southeast Asia | 180145 (118500-231473) | 105.5 (69.4-135.56) | 15057 (11419-19372) | 8.72 (6.61-11.22) | -7.7 (-7.81--7.6) |
| Southern Africa | 89814 (67775-110699) | 225.03 (169.81-277.36) | 22649 (16800-30550) | 32.37 (24.01-43.66) | -5.97 (-6.48--5.45) |
| Southern Latin America | 879 (808-955) | 5.89 (5.41-6.4) | 97 (78-123) | 0.67 (0.54-0.85) | -5.8 (-6.2--5.4) |
| Southern Sub-Saharan Africa | 24989 (21603-28687) | 120.78 (104.42-138.66) | 9011 (6757-11789) | 37.44 (28.08-48.99) | -3.19 (-3.7--2.67) |
| Sub-Saharan Africa - WB | 613900 (439582-764611) | 262.98 (188.3-327.54) | 265624 (187981-374481) | 53.97 (38.19-76.08) | -4.88 (-5.27--4.48) |
| Tropical Latin America | 31948 (27302-36795) | 59.59 (50.92-68.63) | 818 (648-1022) | 1.63 (1.29-2.04) | -11.1 (-11.29--10.91) |
| Western Africa | 272809 (191476-337204) | 344.95 (242.11-426.37) | 139356 (94737-201335) | 73.21 (49.77-105.77) | -4.72 (-5.18--4.26) |
| Western Europe | 176 (162-192) | 0.25 (0.23-0.27) | 146 (125-167) | 0.22 (0.18-0.25) | 0.81 (-0.08-1.71) |
| Western Pacific Region | 109245 (82151-135089) | 25.63 (19.27-31.69) | 6377 (4769-8197) | 1.74 (1.3-2.23) | -8.3 (-8.51--8.09) |
| Western Sub-Saharan Africa | 297407 (210024-367825) | 338.42 (238.99-418.55) | 165359 (113118-238860) | 77 (52.67-111.22) | -4.52 (-4.96--4.08) |
| World Bank High Income | 3022 (2520-3806) | 1.43 (1.19-1.8) | 709 (609-803) | 0.37 (0.31-0.42) | -3.61 (-3.89--3.33) |
| World Bank Low Income | 339411 (250943-432800) | 222.59 (164.57-283.84) | 130178 (91870-186315) | 42.9 (30.28-61.4) | -5.26 (-5.56--4.96) |
| World Bank Lower Middle Income | 1203982 (946752-1417065) | 157.8 (124.08-185.72) | 230353 (176088-302402) | 23.55 (18-30.91) | -5.67 (-5.95--5.38) |
| World Bank Upper Middle Income | 192157 (167831-217000) | 31.47 (27.49-35.54) | 12682 (10372-15328) | 2.37 (1.94-2.86) | -7.77 (-7.93--7.62) |
| Country |  |  |  |  |  |
| Afghanistan | 7356 (4728-10627) | 170.75 (109.75-246.69) | 3983 (2582-6001) | 28.05 (18.18-42.26) | -6.55 (-7.2--5.89) |
| Albania | 100 (73-136) | 8.97 (6.56-12.18) | 6 (3-9) | 1.36 (0.73-2.03) | -6.33 (-7.31--5.35) |
| Algeria | 1098 (415-1818) | 10.24 (3.87-16.95) | 202 (94-295) | 1.52 (0.71-2.21) | -4.87 (-5.22--4.52) |
| American Samoa | 1 (1-1) | 5.17 (3.54-7.39) | 0 (0-1) | 2.53 (1.63-3.76) | -2.16 (-2.41--1.91) |
| Andorra | 0 (0-0) | 0.05 (0.03-0.07) | 0 (0-0) | 0.01 (0.01-0.02) | -4.42 (-4.76--4.07) |
| Angola | 21714 (15143-29186) | 460.55 (321.18-619.03) | 3834 (2518-5407) | 25.15 (16.51-35.47) | -8.96 (-9.75--8.17) |
| Antigua and Barbuda | 1 (1-1) | 3.92 (3.16-4.8) | 0 (0-0) | 1.11 (0.88-1.35) | -4.23 (-4.77--3.68) |
| Argentina | 657 (591-719) | 6.49 (5.83-7.09) | 70 (55-89) | 0.69 (0.54-0.88) | -6.05 (-6.44--5.65) |
| Armenia | 380 (322-444) | 36.46 (30.87-42.6) | 4 (3-5) | 0.68 (0.51-0.9) | -13.57 (-14.16--12.98) |
| Australia | 11 (10-13) | 0.29 (0.26-0.33) | 4 (3-5) | 0.08 (0.06-0.1) | -1.45 (-2.53--0.35) |
| Austria | 2 (2-2) | 0.16 (0.13-0.18) | 1 (1-1) | 0.08 (0.06-0.09) | 0.2 (-0.85-1.27) |
| Azerbaijan | 1507 (1152-1940) | 62.1 (47.48-79.93) | 176 (107-276) | 7.44 (4.54-11.71) | -6.5 (-6.85--6.15) |
| Bahamas | 4 (3-5) | 4.79 (3.8-5.98) | 1 (0-1) | 0.68 (0.49-0.94) | -6.58 (-7.32--5.83) |
| Bahrain | 9 (7-12) | 5.42 (3.99-7.22) | 2 (1-3) | 0.75 (0.5-1) | -5.39 (-5.73--5.04) |
| Bangladesh | 53800 (40057-79695) | 110 (81.9-162.94) | 3937 (2608-5905) | 8.6 (5.7-12.9) | -7.93 (-8.25--7.6) |
| Barbados | 2 (2-2) | 3.11 (2.53-3.7) | 0 (0-0) | 0.59 (0.4-0.84) | -5.51 (-6.01--5) |
| Belarus | 36 (29-47) | 1.51 (1.2-1.95) | 2 (1-3) | 0.13 (0.09-0.19) | -8.55 (-9.2--7.89) |
| Belgium | 9 (8-11) | 0.5 (0.43-0.59) | 11 (8-13) | 0.56 (0.43-0.71) | 1.59 (0.69-2.5) |
| Belize | 27 (24-32) | 33.5 (29.04-38.49) | 4 (3-5) | 2.99 (2.3-3.72) | -7.55 (-8.08--7.02) |
| Benin | 5118 (3377-7240) | 211.35 (139.44-298.94) | 2137 (1039-4236) | 35.15 (17.09-69.67) | -5.35 (-5.63--5.08) |
| Bermuda | 0 (0-0) | 1.71 (1.35-2.1) | 0 (0-0) | 0.19 (0.13-0.25) | -6.8 (-7.63--5.97) |
| Bhutan | 608 (267-1044) | 231.78 (101.78-398.29) | 27 (14-48) | 14.57 (7.46-25.78) | -9.1 (-9.33--8.87) |
| Bolivia (Plurinational State of) | 2329 (1548-3347) | 86.72 (57.63-124.63) | 311 (188-461) | 8.93 (5.41-13.21) | -7.4 (-7.57--7.22) |
| Bosnia and Herzegovina | 16 (12-22) | 1.5 (1.05-2.02) | 4 (3-5) | 0.74 (0.51-1.08) | -1.38 (-1.89--0.87) |
| Botswana | 637 (487-827) | 107.86 (82.41-140.01) | 321 (204-469) | 45.96 (29.21-67.23) | -2.54 (-3--2.08) |
| Brazil | 31412 (26733-36164) | 60.47 (51.46-69.62) | 762 (596-951) | 1.58 (1.24-1.97) | -11.2 (-11.4--11) |
| Brunei Darussalam | 1 (0-1) | 0.57 (0.39-0.77) | 0 (0-1) | 0.42 (0.28-0.59) | -0.37 (-0.62--0.12) |
| Bulgaria | 23 (19-28) | 1.33 (1.1-1.61) | 12 (9-16) | 1.27 (0.95-1.63) | 0.62 (-0.46-1.71) |
| Burkina Faso | 14978 (10017-20323) | 317.4 (212.26-430.67) | 7767 (5188-11353) | 74.88 (50.02-109.45) | -4.57 (-4.85--4.28) |
| Burundi | 4618 (2997-6690) | 176.17 (114.33-255.23) | 2250 (1068-4437) | 38.44 (18.24-75.8) | -4.03 (-4.58--3.48) |
| Cabo Verde | 164 (116-223) | 104.35 (73.58-141.83) | 10 (6-15) | 6.93 (4.53-10.14) | -8.71 (-9.43--7.97) |
| Cambodia | 5688 (4031-7735) | 122.03 (86.48-165.96) | 316 (216-457) | 6.17 (4.22-8.93) | -10 (-10.3--9.7) |
| Cameroon | 7649 (4870-11515) | 156.67 (99.74-235.86) | 4558 (2295-8703) | 33.84 (17.04-64.62) | -4.25 (-4.97--3.51) |
| Canada | 7 (6-9) | 0.13 (0.11-0.15) | 19 (15-23) | 0.3 (0.24-0.38) | 5.39 (3.73-7.07) |
| Central African Republic | 3982 (2422-5419) | 325.69 (198.09-443.24) | 2790 (1643-4487) | 122.17 (71.92-196.48) | -2.71 (-2.94--2.49) |
| Chad | 15486 (10057-21170) | 529.2 (343.67-723.44) | 21093 (14173-35510) | 233.98 (157.22-393.9) | -2.54 (-2.74--2.34) |
| Chile | 167 (150-183) | 4.21 (3.79-4.62) | 20 (16-24) | 0.54 (0.45-0.66) | -5.09 (-5.82--4.35) |
| China | 74923 (55844-94112) | 23.53 (17.54-29.56) | 898 (692-1201) | 0.35 (0.27-0.46) | -13.24 (-13.53--12.95) |
| Colombia | 3584 (3087-4053) | 30.73 (26.47-34.75) | 242 (166-346) | 2.28 (1.57-3.26) | -9.2 (-9.97--8.43) |
| Comoros | 240 (145-377) | 112.61 (68.13-177.3) | 47 (24-76) | 19.61 (10.04-31.8) | -5.52 (-5.93--5.1) |
| Congo | 1658 (1065-2502) | 157.5 (101.15-237.63) | 384 (174-802) | 19.91 (9.02-41.58) | -6.22 (-6.95--5.48) |
| Cook Islands | 0 (0-0) | 0.84 (0.56-1.24) | 0 (0-0) | 0.53 (0.32-0.79) | -3.91 (-4.74--3.07) |
| Costa Rica | 94 (83-105) | 8.35 (7.34-9.35) | 9 (7-11) | 0.84 (0.67-1.04) | -7.15 (-7.85--6.46) |
| Croatia | 6 (5-8) | 0.63 (0.52-0.77) | 5 (4-6) | 0.82 (0.59-1.07) | 2.48 (0.51-4.48) |
| Cuba | 148 (134-163) | 5.89 (5.35-6.49) | 12 (10-15) | 0.7 (0.55-0.87) | -6 (-6.88--5.11) |
| Cyprus | 3 (2-5) | 1.64 (0.8-2.36) | 1 (0-1) | 0.34 (0.23-0.51) | -4.58 (-4.86--4.29) |
| Czechia | 34 (28-41) | 1.56 (1.27-1.87) | 31 (25-39) | 1.83 (1.44-2.26) | 2.41 (0.48-4.38) |
| C?te d'Ivoire | 8703 (6044-11988) | 152.59 (105.98-210.2) | 3605 (2056-6009) | 31.16 (17.77-51.93) | -4.28 (-4.76--3.81) |
| Democratic People's Republic of Korea | 124 (78-193) | 2.09 (1.3-3.24) | 20 (11-31) | 0.43 (0.23-0.65) | -4.62 (-4.81--4.43) |
| Democratic Republic of the Congo | 33863 (23321-45203) | 191.27 (131.73-255.33) | 7725 (3803-13993) | 20.33 (10.01-36.83) | -6.58 (-7.83--5.32) |
| Denmark | 4 (4-5) | 0.5 (0.44-0.57) | 4 (3-5) | 0.43 (0.34-0.52) | 0.49 (-0.2-1.17) |
| Djibouti | 305 (205-435) | 175.27 (117.91-249.64) | 76 (42-135) | 18.43 (10.07-32.61) | -6.84 (-7.28--6.39) |
| Dominica | 2 (1-2) | 6.05 (4.28-8.21) | 0 (0-1) | 3.28 (1.99-5.07) | -1.75 (-2.36--1.14) |
| Dominican Republic | 2178 (1730-2772) | 80.79 (64.18-102.84) | 272 (151-400) | 9.26 (5.14-13.61) | -6.82 (-7.34--6.31) |
| Ecuador | 1902 (1742-2082) | 49.2 (45.07-53.87) | 69 (49-95) | 1.35 (0.97-1.88) | -11.51 (-11.97--11.04) |
| Egypt | 28011 (20477-37379) | 126.28 (92.31-168.51) | 2880 (1935-4127) | 7.81 (5.25-11.2) | -7.88 (-8.28--7.48) |
| El Salvador | 1827 (1488-2215) | 84.68 (68.94-102.62) | 69 (45-100) | 3.79 (2.47-5.49) | -10.22 (-10.9--9.54) |
| Equatorial Guinea | 735 (427-1052) | 373.41 (216.66-534.2) | 45 (23-80) | 7.66 (3.87-13.69) | -13.64 (-14.21--13.06) |
| Eritrea | 4604 (3250-6040) | 289.22 (204.15-379.41) | 1009 (596-1563) | 39.96 (23.61-61.9) | -6.26 (-6.42--6.1) |
| Estonia | 5 (4-6) | 1.4 (1.21-1.6) | 0 (0-0) | 0.08 (0.07-0.11) | -9.37 (-9.83--8.9) |
| Eswatini | 756 (589-948) | 195.87 (152.71-245.74) | 214 (134-321) | 51.82 (32.45-77.89) | -4.02 (-4.61--3.43) |
| Ethiopia | 58261 (30826-89502) | 239.13 (126.52-367.35) | 15580 (11042-22410) | 35.13 (24.9-50.53) | -6.43 (-6.62--6.25) |
| Fiji | 36 (26-50) | 12.83 (9.07-17.89) | 18 (11-29) | 6.76 (4.11-10.51) | -1.35 (-1.73--0.97) |
| Finland | 1 (1-1) | 0.11 (0.1-0.13) | 1 (1-1) | 0.12 (0.09-0.14) | 1.45 (0.4-2.51) |
| France | 57 (50-65) | 0.49 (0.43-0.55) | 32 (26-38) | 0.27 (0.22-0.33) | -1.61 (-2.19--1.03) |
| Gabon | 399 (203-737) | 97.94 (49.91-180.97) | 59 (19-132) | 9.28 (3.03-20.66) | -7.01 (-7.36--6.66) |
| Gambia | 790 (521-1077) | 171.31 (112.85-233.49) | 244 (160-342) | 24.53 (16.14-34.39) | -6.24 (-6.6--5.89) |
| Georgia | 202 (168-239) | 14.73 (12.3-17.5) | 2 (2-3) | 0.3 (0.21-0.41) | -12.65 (-13.26--12.04) |
| Germany | 37 (31-43) | 0.28 (0.24-0.33) | 29 (23-35) | 0.24 (0.19-0.29) | 1.47 (0.6-2.34) |
| Ghana | 8129 (5231-11435) | 121.03 (77.88-170.26) | 1754 (1108-2539) | 13.62 (8.6-19.71) | -6.47 (-6.77--6.16) |
| Greece | 0 (0-1) | 0.02 (0.02-0.03) | 1 (0-1) | 0.05 (0.04-0.06) | 3.79 (3.01-4.58) |
| Greenland | 0 (0-0) | 1.38 (0.61-2.09) | 0 (0-0) | 0.48 (0.32-0.7) | -2.5 (-2.93--2.08) |
| Grenada | 2 (2-2) | 5.76 (4.62-7.29) | 0 (0-0) | 0.82 (0.62-1.05) | -5.64 (-6.39--4.88) |
| Guam | 1 (1-1) | 1.89 (1.39-2.61) | 1 (0-1) | 1.67 (1.1-2.45) | 0.27 (-0.27-0.82) |
| Guatemala | 6245 (5640-6997) | 153.77 (138.89-172.29) | 1050 (801-1369) | 21.29 (16.23-27.75) | -5.74 (-6.04--5.44) |
| Guinea | 8487 (5525-11769) | 308.41 (200.77-427.68) | 1643 (789-3036) | 27.18 (13.05-50.22) | -7.12 (-7.43--6.81) |
| Guinea-Bissau | 1227 (859-1691) | 254.39 (178.06-350.51) | 248 (150-382) | 27.56 (16.65-42.48) | -6.97 (-7.85--6.08) |
| Guyana | 183 (149-220) | 62.39 (50.56-74.88) | 16 (11-22) | 7.35 (5.36-10.1) | -6.79 (-7.15--6.44) |
| Haiti | 9453 (7403-11435) | 348.44 (272.86-421.46) | 3596 (2371-5056) | 82.61 (54.47-116.14) | -4.17 (-4.68--3.65) |
| Honduras | 2318 (1907-2758) | 104.93 (86.32-124.84) | 341 (211-486) | 10.4 (6.42-14.84) | -7.41 (-7.64--7.18) |
| Hungary | 30 (24-37) | 1.42 (1.14-1.76) | 31 (23-39) | 2.2 (1.64-2.81) | 2.9 (0.95-4.89) |
| Iceland | 0 (0-0) | 0.11 (0.1-0.14) | 0 (0-0) | 0.12 (0.09-0.15) | 1.34 (0.41-2.29) |
| India | 527824 (411620-642302) | 161.65 (126.06-196.71) | 46810 (29591-67358) | 12.78 (8.08-18.38) | -7.71 (-8.11--7.32) |
| Indonesia | 115147 (79638-144701) | 169.99 (117.57-213.62) | 9083 (6778-11882) | 13.5 (10.07-17.66) | -7.86 (-7.99--7.73) |
| Iran (Islamic Republic of) | 3625 (2276-6230) | 14.28 (8.97-24.54) | 106 (80-137) | 0.53 (0.39-0.68) | -7.93 (-8.52--7.33) |
| Iraq | 2314 (1587-3275) | 28.1 (19.27-39.77) | 522 (353-740) | 3.88 (2.62-5.5) | -6.7 (-6.99--6.41) |
| Ireland | 1 (1-1) | 0.08 (0.07-0.09) | 1 (1-1) | 0.08 (0.06-0.1) | 1.36 (0.03-2.71) |
| Israel | 10 (8-11) | 0.62 (0.53-0.72) | 12 (9-14) | 0.44 (0.35-0.54) | 0.84 (-0.09-1.77) |
| Italy | 9 (8-9) | 0.09 (0.09-0.1) | 19 (15-23) | 0.24 (0.2-0.3) | 6.79 (4.99-8.62) |
| Jamaica | 161 (139-189) | 19.34 (16.63-22.57) | 10 (7-14) | 1.76 (1.26-2.41) | -6.44 (-7.86--4.99) |
| Japan | 73 (69-78) | 0.32 (0.3-0.34) | 34 (29-38) | 0.22 (0.19-0.25) | -0.54 (-1.21-0.15) |
| Jordan | 100 (69-140) | 6.1 (4.2-8.58) | 41 (29-58) | 1.12 (0.79-1.6) | -5.62 (-5.84--5.41) |
| Kazakhstan | 1666 (1458-1864) | 32.05 (28.07-35.88) | 31 (22-42) | 0.57 (0.41-0.78) | -13.75 (-14.46--13.03) |
| Kenya | 17703 (10955-23064) | 158.48 (98.07-206.48) | 5362 (4004-6967) | 28.73 (21.45-37.33) | -4.91 (-5.41--4.4) |
| Kiribati | 34 (25-47) | 115.7 (84.86-157.89) | 9 (6-14) | 22.6 (14.53-32.81) | -4.78 (-5.1--4.46) |
| Kuwait | 11 (9-13) | 2.01 (1.7-2.34) | 3 (2-3) | 0.31 (0.24-0.4) | -3.3 (-4.36--2.24) |
| Kyrgyzstan | 776 (664-887) | 46.23 (39.59-52.87) | 58 (46-73) | 2.56 (2.02-3.23) | -9.3 (-9.88--8.71) |
| Lao People's Democratic Republic | 5493 (3521-7697) | 298.01 (191.02-417.61) | 410 (255-637) | 17.84 (11.11-27.74) | -9.06 (-9.29--8.83) |
| Latvia | 7 (6-8) | 1.21 (1.01-1.46) | 0 (0-0) | 0.08 (0.06-0.11) | -9.02 (-9.56--8.48) |
| Lebanon | 106 (59-156) | 10.18 (5.68-14.91) | 29 (18-44) | 2.24 (1.42-3.46) | -4.53 (-4.79--4.26) |
| Lesotho | 1457 (1187-1769) | 213.4 (173.95-259.1) | 664 (441-921) | 105.29 (70.01-146.07) | -2.14 (-2.43--1.85) |
| Liberia | 4612 (3296-5947) | 408.08 (291.66-526.19) | 1028 (540-1919) | 47.01 (24.7-87.78) | -6.89 (-7.35--6.43) |
| Libya | 337 (191-525) | 18.62 (10.56-29) | 24 (8-39) | 1.58 (0.56-2.61) | -7.08 (-7.89--6.28) |
| Lithuania | 14 (12-17) | 1.74 (1.47-2.05) | 1 (1-1) | 0.18 (0.14-0.24) | -7.79 (-8.63--6.94) |
| Luxembourg | 0 (0-0) | 0.35 (0.3-0.41) | 0 (0-0) | 0.31 (0.24-0.4) | 0.55 (-0.07-1.16) |
| Madagascar | 15969 (12545-19611) | 292.69 (229.93-359.44) | 9531 (5595-15732) | 81.23 (47.69-134.08) | -3.53 (-3.88--3.17) |
| Malawi | 17322 (11013-22855) | 380.73 (242.07-502.35) | 2769 (1695-4085) | 34.09 (20.87-50.29) | -7.74 (-7.99--7.49) |
| Malaysia | 415 (284-552) | 6.32 (4.32-8.4) | 90 (63-125) | 1.18 (0.83-1.65) | -4.72 (-5.49--3.94) |
| Maldives | 138 (92-175) | 131.32 (87.55-166.8) | 4 (3-5) | 3.73 (2.55-5.3) | -10.4 (-11.33--9.46) |
| Mali | 11614 (7259-16438) | 281.23 (175.78-398.03) | 4516 (2971-6985) | 39.01 (25.67-60.34) | -6.02 (-6.39--5.65) |
| Malta | 0 (0-0) | 0.06 (0.05-0.07) | 0 (0-0) | 0.11 (0.09-0.15) | 3.85 (2.95-4.75) |
| Marshall Islands | 3 (2-5) | 14.69 (8.4-22.53) | 1 (1-1) | 5.37 (3.26-8.12) | -3.08 (-3.9--2.25) |
| Mauritania | 1362 (838-2089) | 147.31 (90.63-226.05) | 346 (172-653) | 18.69 (9.28-35.25) | -6.44 (-7.08--5.8) |
| Mauritius | 30 (27-33) | 8.95 (8.06-10.09) | 5 (4-6) | 2.26 (1.82-2.67) | -3.01 (-3.46--2.56) |
| Mexico | 22467 (20262-25168) | 67.23 (60.64-75.32) | 865 (645-1140) | 2.7 (2.01-3.55) | -9.6 (-10.02--9.16) |
| Micronesia (Federated States of) | 8 (5-11) | 17.09 (11.5-24.81) | 1 (1-2) | 3.29 (2.08-4.94) | -4.94 (-6.14--3.72) |
| Monaco | 0 (0-0) | 0.09 (0.04-0.13) | 0 (0-0) | 0.09 (0.06-0.13) | -2.13 (-3.01--1.24) |
| Mongolia | 207 (123-333) | 23.05 (13.65-37.02) | 23 (9-49) | 2.11 (0.82-4.53) | -6.75 (-7.15--6.35) |
| Montenegro | 0 (0-1) | 0.3 (0.2-0.46) | 0 (0-0) | 0.07 (0.04-0.13) | -4.33 (-4.72--3.94) |
| Morocco | 11127 (8517-14261) | 113.7 (87.04-145.73) | 656 (400-1031) | 6.7 (4.08-10.52) | -8.55 (-8.88--8.23) |
| Mozambique | 15577 (9470-23630) | 251.07 (152.64-380.87) | 4494 (2657-7439) | 31.5 (18.62-52.14) | -6.59 (-6.98--6.2) |
| Myanmar | 27298 (13338-44441) | 184.74 (90.27-300.76) | 1896 (1271-2757) | 12.15 (8.14-17.65) | -8.74 (-8.93--8.54) |
| Namibia | 854 (633-1109) | 142.23 (105.37-184.63) | 367 (220-544) | 44.48 (26.69-65.95) | -3.22 (-3.58--2.85) |
| Nauru | 1 (0-1) | 12.55 (8.2-18.29) | 0 (0-0) | 5.33 (3.37-8.09) | -2.71 (-3.36--2.06) |
| Nepal | 14733 (11106-19064) | 174.87 (131.82-226.27) | 746 (468-1095) | 8.09 (5.07-11.87) | -9.7 (-9.92--9.48) |
| Netherlands | 5 (4-5) | 0.17 (0.15-0.19) | 6 (5-7) | 0.23 (0.19-0.28) | 2.06 (1.33-2.8) |
| New Zealand | 4 (3-4) | 0.47 (0.41-0.53) | 3 (2-3) | 0.29 (0.24-0.35) | 2.29 (0.34-4.28) |
| Nicaragua | 2134 (1724-2753) | 117.18 (94.69-151.14) | 104 (63-151) | 5.27 (3.19-7.61) | -9.74 (-10.21--9.25) |
| Niger | 23765 (17464-30445) | 584.93 (429.85-749.33) | 10946 (6859-17485) | 85.76 (53.74-136.98) | -6.62 (-7.1--6.13) |
| Nigeria | 165703 (114492-205853) | 423.53 (292.63-526.15) | 101195 (66645-145969) | 99.61 (65.6-143.69) | -4.39 (-4.9--3.87) |
| Niue | 0 (0-0) | 6.18 (4.33-8.98) | 0 (0-0) | 9.37 (5.57-12.86) | -1.31 (-2.24--0.37) |
| North Macedonia | 127 (99-167) | 24.08 (18.78-31.66) | 7 (5-10) | 2.06 (1.48-2.91) | -6.25 (-7.4--5.07) |
| Northern Mariana Islands | 0 (0-0) | 1.75 (1.22-2.46) | 0 (0-0) | 0.95 (0.66-1.28) | -1.79 (-2.13--1.45) |
| Norway | 0 (0-1) | 0.06 (0.05-0.07) | 1 (1-1) | 0.08 (0.07-0.1) | 2.96 (1.69-4.24) |
| Oman | 80 (43-138) | 9.51 (5.11-16.46) | 18 (12-26) | 1.48 (0.97-2.12) | -4.24 (-5.37--3.11) |
| Pakistan | 82095 (65234-102672) | 166.72 (132.48-208.5) | 17620 (11249-26993) | 20.62 (13.17-31.59) | -5.68 (-6.08--5.28) |
| Palau | 1 (0-1) | 12.81 (7.83-20.98) | 0 (0-0) | 4.26 (2.93-6.11) | -3.39 (-3.53--3.25) |
| Palestine | 154 (101-239) | 15.93 (10.43-24.72) | 17 (12-26) | 0.92 (0.62-1.39) | -8.27 (-8.66--7.87) |
| Panama | 178 (147-210) | 21.3 (17.65-25.16) | 62 (46-82) | 5.39 (4-7.11) | -3.3 (-3.69--2.91) |
| Papua New Guinea | 1692 (1143-2508) | 99.5 (67.2-147.51) | 1689 (1015-2643) | 43.11 (25.91-67.47) | -2.07 (-2.32--1.82) |
| Paraguay | 537 (405-699) | 32.15 (24.27-41.87) | 57 (31-97) | 2.83 (1.57-4.82) | -8.34 (-8.63--8.05) |
| Peru | 3981 (3125-4982) | 47.96 (37.65-60.02) | 252 (148-385) | 2.64 (1.55-4.03) | -9.4 (-9.94--8.87) |
| Philippines | 17056 (12268-22402) | 67.64 (48.66-88.85) | 2729 (2047-3730) | 8.03 (6.02-10.97) | -6.34 (-6.53--6.15) |
| Poland | 61 (53-68) | 0.63 (0.56-0.71) | 41 (32-51) | 0.69 (0.54-0.86) | 3.19 (0.84-5.59) |
| Portugal | 13 (11-14) | 0.59 (0.52-0.68) | 2 (2-3) | 0.16 (0.13-0.19) | -3.92 (-4.5--3.33) |
| Puerto Rico | 15 (13-17) | 1.49 (1.32-1.7) | 2 (2-3) | 0.53 (0.42-0.67) | -4.39 (-5.5--3.28) |
| Qatar | 3 (2-5) | 2.51 (1.64-3.68) | 3 (2-4) | 0.53 (0.35-0.76) | -4.13 (-4.4--3.87) |
| Republic of Korea | 119 (85-165) | 1.05 (0.75-1.45) | 14 (10-20) | 0.24 (0.16-0.34) | -4.19 (-4.5--3.89) |
| Republic of Moldova | 95 (78-114) | 7.68 (6.31-9.23) | 4 (2-5) | 0.69 (0.48-0.99) | -7.62 (-8.31--6.92) |
| Romania | 322 (271-376) | 5.78 (4.87-6.75) | 40 (31-51) | 1.34 (1.02-1.69) | -4.37 (-5.16--3.56) |
| Russian Federation | 1095 (1045-1149) | 3.15 (3.01-3.31) | 80 (70-89) | 0.3 (0.27-0.34) | -7.25 (-7.74--6.75) |
| Rwanda | 6486 (4011-9197) | 191.16 (118.2-271.07) | 1295 (833-1909) | 26.05 (16.77-38.41) | -7.16 (-7.95--6.36) |
| Saint Kitts and Nevis | 3 (3-4) | 21.93 (19.19-24.93) | 0 (0-1) | 3.94 (2.91-5.42) | -5.22 (-6.03--4.41) |
| Saint Lucia | 5 (4-7) | 10.5 (8.52-12.86) | 0 (0-1) | 1.47 (1.05-2.09) | -6.03 (-6.91--5.14) |
| Saint Vincent and the Grenadines | 8 (6-10) | 18.93 (14.84-23.53) | 1 (0-1) | 2.16 (1.61-2.89) | -6.52 (-7.19--5.83) |
| Samoa | 2 (1-4) | 3.45 (1.42-5.52) | 1 (0-2) | 1.35 (0.62-2.15) | -2.83 (-3.17--2.5) |
| San Marino | 0 (0-0) | 0.11 (0.06-0.16) | 0 (0-0) | 0.03 (0.02-0.06) | -3.54 (-3.71--3.36) |
| Sao Tome and Principe | 92 (70-115) | 161.52 (123.13-203.57) | 5 (3-7) | 6.24 (4.06-9.37) | -10.18 (-10.86--9.5) |
| Saudi Arabia | 1259 (800-1970) | 19.21 (12.21-30.06) | 84 (50-123) | 1.11 (0.66-1.63) | -8.81 (-9.05--8.57) |
| Senegal | 9675 (7246-12201) | 264.98 (198.44-334.15) | 1143 (710-1682) | 17.96 (11.15-26.44) | -8.31 (-8.94--7.67) |
| Serbia | 37 (15-57) | 1.69 (0.7-2.61) | 5 (3-8) | 0.4 (0.26-0.63) | -4.47 (-5.02--3.92) |
| Seychelles | 1 (1-2) | 5.73 (4.21-7.61) | 1 (0-1) | 2.18 (1.39-3.15) | -2.21 (-2.79--1.62) |
| Sierra Leone | 5621 (3719-8153) | 310.08 (205.2-449.8) | 1587 (996-2615) | 44.37 (27.84-73.11) | -6.42 (-7.03--5.81) |
| Singapore | 5 (4-6) | 0.74 (0.66-0.85) | 1 (1-2) | 0.17 (0.13-0.21) | -3.11 (-3.84--2.37) |
| Slovakia | 9 (6-12) | 0.68 (0.48-0.9) | 5 (3-9) | 0.64 (0.38-1.08) | 1.13 (0.39-1.88) |
| Slovenia | 1 (1-1) | 0.25 (0.2-0.31) | 1 (1-1) | 0.24 (0.18-0.33) | 1.72 (-0.08-3.55) |
| Solomon Islands | 53 (34-79) | 34.33 (22.11-51.04) | 19 (12-26) | 7.16 (4.7-10.07) | -4.58 (-4.78--4.38) |
| Somalia | 12858 (7345-18425) | 330.06 (188.54-472.96) | 10473 (6221-15588) | 101.39 (60.22-150.9) | -3.9 (-4.09--3.71) |
| South Africa | 18873 (16059-21977) | 138.64 (117.97-161.43) | 5199 (4050-6741) | 34.19 (26.63-44.33) | -3.86 (-4.34--3.38) |
| South Sudan | 9821 (6107-13886) | 374.27 (232.74-529.17) | 7494 (4702-11133) | 174.49 (109.47-259.21) | -2.21 (-2.39--2.02) |
| Spain | 15 (13-16) | 0.19 (0.16-0.21) | 11 (9-13) | 0.18 (0.14-0.21) | 1.36 (0.27-2.47) |
| Sri Lanka | 1008 (772-1331) | 18.22 (13.96-24.06) | 57 (38-81) | 1.12 (0.75-1.59) | -8.12 (-8.51--7.73) |
| Sudan | 15493 (8227-27851) | 174.23 (92.52-313.19) | 1725 (684-4559) | 10.4 (4.12-27.48) | -8.83 (-9.34--8.32) |
| Suriname | 59 (43-78) | 45.51 (33.07-59.9) | 13 (9-19) | 9.35 (5.96-13.39) | -5.2 (-5.49--4.91) |
| Sweden | 0 (0-0) | 0.02 (0.02-0.03) | 3 (2-3) | 0.15 (0.12-0.17) | 8.34 (6.98-9.71) |
| Switzerland | 3 (3-4) | 0.3 (0.26-0.34) | 3 (2-4) | 0.22 (0.17-0.26) | 0.4 (-0.37-1.18) |
| Syrian Arab Republic | 758 (497-1135) | 12.8 (8.39-19.17) | 30 (18-45) | 0.82 (0.48-1.22) | -8.12 (-8.76--7.49) |
| Taiwan (Province of China) | 69 (62-76) | 1.26 (1.13-1.38) | 4 (3-5) | 0.13 (0.1-0.15) | -7.05 (-7.94--6.15) |
| Tajikistan | 3419 (2769-4204) | 147.26 (119.24-181.04) | 1334 (903-1871) | 37.22 (25.2-52.21) | -4.61 (-4.79--4.43) |
| Thailand | 3273 (1375-5568) | 19.41 (8.16-33.03) | 254 (190-331) | 2.6 (1.94-3.39) | -5.95 (-6.38--5.51) |
| Timor-Leste | 940 (525-1292) | 282.52 (157.96-388.3) | 85 (50-130) | 16.25 (9.66-24.91) | -9.72 (-10.17--9.26) |
| Togo | 4222 (2733-6273) | 239.51 (155.07-355.91) | 1534 (829-2723) | 46.35 (25.07-82.29) | -4.71 (-5.23--4.18) |
| Tokelau | 0 (0-0) | 17.25 (9.62-29.4) | 0 (0-0) | 24.96 (15.34-39.53) | -3.53 (-5.47--1.55) |
| Tonga | 2 (1-3) | 4.16 (2.59-6.16) | 1 (0-1) | 1.42 (0.8-2.33) | -2.8 (-3.01--2.6) |
| Trinidad and Tobago | 33 (26-39) | 8.06 (6.45-9.7) | 4 (3-5) | 1.38 (0.99-1.93) | -5.49 (-5.77--5.21) |
| Tunisia | 311 (138-488) | 10 (4.44-15.7) | 29 (12-45) | 1.03 (0.42-1.63) | -6.05 (-6.54--5.55) |
| Turkmenistan | 1587 (1372-1849) | 105.75 (91.42-123.23) | 45 (33-60) | 2.92 (2.14-3.91) | -12.83 (-13.47--12.19) |
| Tuvalu | 1 (1-2) | 35.59 (20.4-54.1) | 0 (0-0) | 4.02 (2.68-5.7) | -6.28 (-6.95--5.6) |
| Türkiye | 5313 (3677-8327) | 25.93 (17.95-40.64) | 256 (183-362) | 1.38 (0.99-1.95) | -9.31 (-9.49--9.12) |
| Uganda | 16890 (7583-26956) | 200.6 (90.07-320.16) | 4474 (2361-7939) | 22.56 (11.9-40.02) | -6.85 (-7.08--6.62) |
| Ukraine | 183 (157-214) | 1.61 (1.38-1.88) | 12 (9-15) | 0.19 (0.15-0.24) | -7.87 (-8.57--7.17) |
| United Arab Emirates | 25 (15-35) | 4.18 (2.54-5.99) | 13 (9-17) | 0.94 (0.66-1.29) | -2.24 (-3.04--1.44) |
| United Kingdom | 7 (6-7) | 0.06 (0.06-0.07) | 11 (9-12) | 0.09 (0.08-0.1) | 1.66 (-1.13-4.52) |
| United Republic of Tanzania | 23078 (16462-31367) | 191.11 (136.32-259.76) | 7368 (4467-11140) | 30.19 (18.31-45.65) | -5.75 (-6.05--5.45) |
| United States of America | 220 (208-234) | 0.39 (0.37-0.42) | 108 (95-123) | 0.18 (0.16-0.21) | -2.66 (-3.18--2.15) |
| United States Virgin Islands | 1 (1-1) | 2.59 (1.75-3.77) | 0 (0-0) | 0.71 (0.41-1.09) | -3.91 (-4.19--3.62) |
| Uruguay | 54 (48-61) | 6.63 (5.91-7.49) | 7 (5-9) | 1.09 (0.83-1.39) | -5.45 (-5.83--5.07) |
| Uzbekistan | 4090 (3596-4639) | 47.8 (42.03-54.22) | 87 (65-117) | 0.87 (0.64-1.16) | -13.53 (-14.38--12.67) |
| Vanuatu | 20 (11-32) | 28.79 (16.6-47.26) | 9 (5-16) | 7.67 (4.18-13.88) | -4.01 (-4.23--3.79) |
| Venezuela (Bolivarian Republic of) | 3249 (3035-3488) | 45.8 (42.79-49.18) | 497 (246-693) | 7.51 (3.72-10.47) | -6 (-6.89--5.12) |
| Viet Nam | 3399 (1649-5488) | 12.82 (6.22-20.7) | 108 (59-166) | 0.43 (0.24-0.67) | -10.06 (-10.43--9.69) |
| Yemen | 14413 (9529-20824) | 203.16 (134.32-293.52) | 1390 (510-2766) | 10.08 (3.7-20.06) | -9.22 (-9.66--8.77) |
| Zambia | 10214 (6593-14191) | 272.04 (175.61-377.96) | 2541 (1655-3694) | 30.72 (20-44.65) | -6.85 (-7.51--6.18) |
| Zimbabwe | 2412 (1726-3042) | 50.08 (35.83-63.16) | 2246 (1283-3257) | 35.68 (20.38-51.74) | -0.24 (-1.23-0.77) |

eTable 3. DALYs of diarrhea disease in children at the global, sex, age-group levels, regional and national levels from 1990 to 2021.

|  | Rate per 100 000(95% UI) | |  | |  |
| --- | --- | --- | --- | --- | --- |
|  | 1990 | | 2021 | | 1990-2021 |
|  | DALYs cases | DALYs rate | DALYs cases | DALYs rate | EAPC |
| Global | 158413390 (126760674-185602330) | 9108.66 (7288.65-10672.01) | 35897190 (27389119-46679614) | 1784.28 (1361.38-2320.22) | -4.85 (-5.15--4.56) |
| Sex |  |  |  |  |  |
| Female | 74908185 (60024767-88806007) | 8857.96 (7097.98-10501.39) | 16503879 (12727309-21250218) | 1694.97 (1307.11-2182.43) | -4.92 (-5.26--4.58) |
| Male | 83505205 (62158819-103713723) | 9345.94 (6956.84-11607.69) | 19393312 (13762516-26924602) | 1868.04 (1325.66-2593.48) | -4.8 (-5.05--4.54) |
| Age |  |  |  |  |  |
| <5 years | 147785002 (116823042-173680475) | 23838.68 (18844.31-28015.78) | 30931280 (23118226-41966936) | 4699.58 (3512.49-6376.29) | NA |
| 5-9 years | 7108937 (4661437-9722175) | 1218.26 (798.83-1666.09) | 2675633 (1816153-3921738) | 389.44 (264.34-570.81) | NA |
| 10-14 years | 3519451 (2489451-4849749) | 657 (464.72-905.34) | 2290278 (1667694-3130116) | 343.56 (250.17-469.54) | NA |
| SDI region |  |  |  |  |  |
| High-middle SDI | 3362598 (2758712-3973494) | 1228.92 (1008.22-1452.18) | 281851 (221045-347787) | 122.07 (95.74-150.63) | -6.93 (-7.1--6.75) |
| High SDI | 334090 (259655-420058) | 179.8 (139.74-226.07) | 138983 (99353-187128) | 80.55 (57.58-108.46) | -1.87 (-2.13--1.6) |
| Low-middle SDI | 72050129 (59031650-83983357) | 15261.23 (12503.73-17788.86) | 9692659 (7782839-12297503) | 1671.61 (1342.24-2120.84) | -6.7 (-6.96--6.43) |
| Low SDI | 54223241 (40610342-67507253) | 23687.22 (17740.48-29490.3) | 22623976 (16518315-30436449) | 4915.84 (3589.18-6613.38) | -4.89 (-5.21--4.57) |
| Middle SDI | 28348316 (22528152-33406278) | 4911.23 (3902.91-5787.5) | 3129480 (2494521-3866863) | 552.07 (440.06-682.15) | -6.51 (-6.65--6.37) |
| GBD region |  |  |  |  |  |
| Advanced Health System | 1109282 (910385-1428870) | 381.85 (313.38-491.86) | 230738 (171175-302407) | 91.85 (68.14-120.37) | -4.08 (-4.22--3.94) |
| Africa | 59156591 (43838115-72652871) | 21035.28 (15588.24-25834.37) | 24834452 (17959427-34292470) | 4460.12 (3225.41-6158.73) | -4.74 (-5.14--4.34) |
| African Region | 52929561 (37574955-66033896) | 22875.65 (16239.53-28539.21) | 23323876 (16660149-32789452) | 4873.61 (3481.2-6851.48) | -4.74 (-5.16--4.32) |
| America | 8850482 (8184421-9529132) | 4033.56 (3730-4342.85) | 875064 (676898-1110739) | 393.25 (304.19-499.16) | -7.2 (-7.51--6.9) |
| Andean Latin America | 782964 (666103-915696) | 5271.77 (4484.93-6165.46) | 66014 (46513-87809) | 364.82 (257.05-485.27) | -8.69 (-8.92--8.46) |
| Asia | 89253908 (72666533-104603693) | 8414.88 (6851.01-9862.06) | 9985661 (7675958-12952010) | 916.16 (704.25-1188.31) | -6.64 (-6.91--6.36) |
| Australasia | 3276 (2495-4366) | 71.45 (54.41-95.2) | 1314 (1013-1731) | 22.92 (17.68-30.2) | -2.18 (-2.65--1.71) |
| Basic Health System | 34140176 (28491594-39237630) | 4618.89 (3854.68-5308.54) | 2927556 (2404741-3556687) | 421.01 (345.82-511.48) | -7.14 (-7.31--6.98) |
| Caribbean | 1147514 (943540-1353018) | 10054.95 (8267.66-11855.66) | 370982 (254899-511888) | 3224.5 (2215.53-4449.23) | -3.21 (-3.72--2.71) |
| Central Africa | 6188274 (4329959-7898855) | 19917.18 (13936.13-25422.75) | 3573598 (2361495-5820542) | 4973.7 (3286.7-8100.97) | -4.3 (-4.95--3.66) |
| Central Asia | 1269276 (1145070-1413357) | 5078.89 (4581.89-5655.42) | 165572 (121361-220565) | 598.26 (438.51-796.96) | -7.2 (-7.5--6.89) |
| Central Europe | 75537 (67851-83948) | 256.2 (230.13-284.73) | 18484 (15382-21537) | 104.42 (86.9-121.67) | -2.13 (-3.12--1.13) |
| Central Latin America | 3863553 (3582555-4199362) | 6001.06 (5564.6-6522.65) | 324788 (249385-415470) | 511.6 (392.83-654.44) | -7.52 (-7.96--7.08) |
| Central Sub-Saharan Africa | 5625950 (3981253-7059754) | 22238.16 (15737.03-27905.68) | 1415474 (910379-2103007) | 2412.12 (1551.39-3583.76) | -6.71 (-7.67--5.74) |
| Commonwealth High Income | 24610 (17653-34358) | 107.52 (77.13-150.11) | 12435 (8794-17042) | 49.1 (34.72-67.29) | -1.63 (-2.52--0.73) |
| Commonwealth Low Income | 14234883 (10358987-17840882) | 14745.66 (10730.69-18481.05) | 2991359 (2115233-4160644) | 2142.71 (1515.14-2980.27) | -5.94 (-6.12--5.77) |
| Commonwealth Middle Income | 74959699 (60213890-89220258) | 16211.98 (13022.81-19296.19) | 17617483 (13663994-22827851) | 2820.63 (2187.66-3654.83) | -5.27 (-5.58--4.97) |
| East Asia | 7037108 (5334164-8756072) | 2133.53 (1617.23-2654.69) | 139431 (108850-175174) | 52.15 (40.71-65.52) | -11.88 (-12.22--11.54) |
| East Asia & Pacific - WB | 23649989 (16722381-29391819) | 4399.76 (3110.97-5467.95) | 1923382 (1526574-2351676) | 410.61 (325.9-502.04) | -7.21 (-7.35--7.07) |
| Eastern Africa | 16441429 (11598209-21542824) | 19899.39 (14037.54-26073.71) | 6015631 (4365285-8199181) | 3793.42 (2752.72-5170.35) | -5.26 (-5.43--5.09) |
| Eastern Europe | 189203 (167793-216887) | 367.66 (326.05-421.45) | 25276 (18291-33915) | 71.31 (51.6-95.68) | -4.89 (-5.15--4.63) |
| Eastern Mediterranean Region | 16557402 (13854315-19773516) | 10188.73 (8525.36-12167.79) | 3863986 (2840702-5237021) | 1561.12 (1147.7-2115.85) | -5.44 (-5.67--5.21) |
| Eastern Sub-Saharan Africa | 19338231 (12656906-26123279) | 21351.52 (13974.61-28842.96) | 6957216 (4977983-9653583) | 3899.1 (2789.86-5410.25) | -5.38 (-5.58--5.19) |
| Europe | 1054643 (867878-1361189) | 601.82 (495.24-776.74) | 170889 (130418-221387) | 121.6 (92.8-157.53) | -4.74 (-4.88--4.61) |
| Europe & Central Asia - WB | 2099748 (1863690-2465931) | 1083.01 (961.26-1271.88) | 315803 (251450-392321) | 193.69 (154.22-240.62) | -5.68 (-5.89--5.47) |
| European Region | 2113907 (1876372-2482825) | 1078.45 (957.26-1266.66) | 319218 (254325-396310) | 192.24 (153.16-238.66) | -5.67 (-5.88--5.46) |
| High-income Asia Pacific | 51249 (36424-68993) | 145.59 (103.48-196) | 32177 (21058-45937) | 143.48 (93.9-204.84) | 0.49 (0.2-0.78) |
| High-income North America | 42783 (33895-55577) | 69.36 (54.96-90.11) | 13174 (11447-14870) | 20.08 (17.44-22.66) | -3.42 (-3.9--2.94) |
| Latin America & Caribbean - WB | 8810174 (8140896-9489477) | 5548.42 (5126.93-5976.23) | 862552 (665438-1096666) | 548.09 (422.84-696.85) | -7.16 (-7.47--6.85) |
| Limited Health System | 106974984 (84640489-126422722) | 16597 (13131.84-19614.28) | 24472366 (18696835-31887482) | 2684.31 (2050.81-3497.66) | -5.54 (-5.81--5.28) |
| Middle East & North Africa - WB | 5956738 (4717098-7295647) | 5489.37 (4346.99-6723.23) | 676055 (501297-875424) | 493.67 (366.06-639.25) | -6.97 (-7.13--6.81) |
| Minimal Health System | 16093932 (11376271-20069207) | 25353.43 (17921.51-31615.85) | 8236288 (5794365-11815227) | 5415.76 (3810.08-7769.09) | -4.95 (-5.36--4.54) |
| North Africa and Middle East | 8513194 (6451687-10798416) | 6059.84 (4592.43-7686.5) | 1258454 (937891-1759388) | 686.47 (511.61-959.73) | -6.6 (-6.82--6.38) |
| North America | 42790 (33896-55584) | 69.38 (54.96-90.13) | 13182 (11451-14878) | 20.09 (17.45-22.67) | -3.42 (-3.89--2.94) |
| Northern Africa | 3880790 (3016751-4954147) | 7996.2 (6215.89-10207.81) | 439730 (329476-574696) | 665.66 (498.76-869.97) | -7.12 (-7.34--6.9) |
| Oceania | 182375 (129926-264278) | 6805.37 (4848.24-9861.61) | 171829 (108873-263151) | 3381.88 (2142.8-5179.25) | -1.69 (-1.91--1.46) |
| Region of the Americas | 8850482 (8184421-9529132) | 4033.56 (3730-4342.85) | 875064 (676898-1110739) | 393.25 (304.19-499.16) | -7.2 (-7.51--6.9) |
| South-East Asia Region | 67556945 (53894046-81009987) | 13636.52 (10878.63-16352.04) | 6717207 (4966177-8956953) | 1280.08 (946.39-1706.91) | -7.31 (-7.53--7.08) |
| South Asia | 61600304 (50207351-73760584) | 14214.52 (11585.55-17020.55) | 7273101 (5298682-9701403) | 1434.48 (1045.06-1913.41) | -6.96 (-7.23--6.68) |
| South Asia - WB | 62375192 (50743355-74680779) | 14070.4 (11446.52-16846.25) | 7652626 (5592482-10128781) | 1453.69 (1062.35-1924.06) | -6.91 (-7.18--6.63) |
| Southeast Asia | 16507496 (10991965-21027703) | 9667.78 (6437.55-12315.08) | 1599799 (1269047-1989337) | 926.59 (735.03-1152.21) | -7.27 (-7.39--7.15) |
| Southern Africa | 8132223 (6184899-9995724) | 20375.16 (15496.17-25044.14) | 2117559 (1596253-2796125) | 3026.26 (2281.25-3996.02) | -5.89 (-6.39--5.38) |
| Southern Latin America | 99286 (88683-110197) | 665.17 (594.13-738.27) | 14278 (11397-17954) | 98.5 (78.63-123.86) | -4.83 (-5.33--4.33) |
| Southern Sub-Saharan Africa | 2311316 (2003880-2648238) | 11171.54 (9685.57-12800.02) | 835390 (638923-1074881) | 3471.27 (2654.89-4466.42) | -3.2 (-3.71--2.69) |
| Sub-Saharan Africa - WB | 55371971 (40068938-68750122) | 23719.7 (17164.34-29450.5) | 24422671 (17581878-33930513) | 4962.02 (3572.16-6893.76) | -4.82 (-5.22--4.43) |
| Tropical Latin America | 2955747 (2531200-3388127) | 5513.02 (4721.16-6319.48) | 99058 (80340-119317) | 197.35 (160.06-237.72) | -10.48 (-10.64--10.32) |
| Western Africa | 24513875 (17382987-30149666) | 30995.89 (21979.44-38121.91) | 12687933 (8718585-18215900) | 6665.63 (4580.33-9569.76) | -4.68 (-5.14--4.22) |
| Western Europe | 89563 (59825-127945) | 126.11 (84.24-180.16) | 74393 (50350-105012) | 109.21 (73.92-154.16) | 0.07 (-0.53-0.67) |
| Western Pacific Region | 10297613 (7874700-12710414) | 2415.57 (1847.22-2981.56) | 761841 (600534-934118) | 207.33 (163.43-254.21) | -7.61 (-7.83--7.39) |
| Western Sub-Saharan Africa | 26727468 (18973861-32874376) | 30413.66 (21590.69-37408.33) | 15040987 (10424598-21603703) | 7003.5 (4853.98-10059.29) | -4.49 (-4.93--4.05) |
| World Bank High Income | 425406 (343290-518539) | 201.01 (162.21-245.01) | 172203 (128038-225935) | 88.99 (66.17-116.76) | -1.89 (-2.12--1.66) |
| World Bank Low Income | 30645078 (22772220-38949509) | 20097.67 (14934.49-25543.89) | 12064504 (8652709-17063394) | 3975.94 (2851.56-5623.35) | -5.18 (-5.47--4.88) |
| World Bank Lower Middle Income | 109201706 (86456188-127632938) | 14312.14 (11331.08-16727.77) | 22238002 (17289974-28800811) | 2273.29 (1767.47-2944.17) | -5.5 (-5.76--5.24) |
| World Bank Upper Middle Income | 18017731 (15848816-20211568) | 2951.09 (2595.85-3310.41) | 1361605 (1132113-1610357) | 254.42 (211.54-300.9) | -7.4 (-7.55--7.26) |
| Country |  |  |  |  |  |
| Afghanistan | 663083 (429834-953939) | 15391.65 (9977.41-22143.06) | 366931 (242261-547235) | 2583.91 (1705.99-3853.59) | -6.47 (-7.11--5.83) |
| Albania | 9322 (6934-12534) | 834.39 (620.66-1121.83) | 593 (346-859) | 133.62 (78.03-193.62) | -6.12 (-6.96--5.26) |
| Algeria | 117822 (57485-183020) | 1098.61 (536.01-1706.54) | 30059 (18994-41065) | 225.98 (142.79-308.72) | -4.17 (-4.46--3.89) |
| American Samoa | 109 (81-146) | 574.58 (424.66-765.49) | 55 (39-74) | 388.4 (278.25-519.49) | -1.2 (-1.38--1.01) |
| Andorra | 10 (6-16) | 109.14 (67.02-167.6) | 11 (6-17) | 109.82 (63.28-172.06) | 0.31 (-0.25-0.87) |
| Angola | 1944452 (1363759-2607603) | 41241.49 (28925.09-55306.82) | 369612 (252789-506626) | 2424.41 (1658.12-3323.12) | -8.79 (-9.55--8.02) |
| Antigua and Barbuda | 79 (66-95) | 436.94 (363.62-524.07) | 35 (27-47) | 208.69 (156.82-280.28) | -2.51 (-2.86--2.15) |
| Argentina | 75256 (66713-83920) | 742.52 (658.23-828) | 10647 (8278-13596) | 104.55 (81.29-133.51) | -4.87 (-5.47--4.28) |
| Armenia | 35590 (30358-41459) | 3411.63 (2910.08-3974.21) | 531 (414-678) | 89.63 (69.93-114.51) | -12.24 (-12.64--11.83) |
| Australia | 2342 (1806-3095) | 61.87 (47.7-81.76) | 775 (584-1066) | 16.31 (12.3-22.44) | -2.72 (-3.19--2.25) |
| Austria | 3038 (1964-4384) | 225.31 (145.63-325.15) | 1199 (807-1720) | 92.47 (62.22-132.58) | -3.18 (-4.14--2.2) |
| Azerbaijan | 137614 (106197-176364) | 5670.29 (4375.76-7266.95) | 16731 (10906-25893) | 708.75 (462-1096.89) | -6.37 (-6.71--6.04) |
| Bahamas | 405 (327-499) | 501.7 (405.24-618.36) | 102 (75-138) | 125.62 (92.02-169.87) | -4.27 (-4.77--3.76) |
| Bahrain | 922 (712-1190) | 564.85 (435.95-729.19) | 379 (275-498) | 127.84 (92.63-167.7) | -4.22 (-4.49--3.94) |
| Bangladesh | 4937514 (3754966-7263500) | 10094.8 (7677.07-14850.3) | 406207 (287424-574895) | 887.57 (628.03-1256.16) | -7.56 (-7.88--7.23) |
| Barbados | 232 (188-277) | 371.63 (301.36-444.42) | 91 (59-132) | 192.65 (124.7-280.66) | -2.06 (-2.36--1.75) |
| Belarus | 5240 (4299-6493) | 217.97 (178.83-270.12) | 1077 (692-1565) | 68.23 (43.83-99.14) | -4.07 (-4.41--3.73) |
| Belgium | 2557 (1791-3594) | 141.55 (99.14-199.01) | 2101 (1569-2772) | 109.86 (82.08-144.98) | -0.43 (-1.07-0.23) |
| Belize | 2537 (2217-2896) | 3099.69 (2708.77-3538.02) | 437 (349-552) | 355.15 (283.6-448.35) | -6.73 (-7.25--6.2) |
| Benin | 461318 (308063-646180) | 19048.71 (12720.51-26682.06) | 198825 (101744-384026) | 3269.93 (1673.31-6315.8) | -5.27 (-5.54--5) |
| Bermuda | 29 (23-35) | 243.12 (195.15-296.69) | 14 (8-21) | 161.25 (98.25-244.65) | -1.13 (-1.45--0.81) |
| Bhutan | 54317 (24225-92563) | 20716.39 (9239.43-35303.05) | 2611 (1410-4479) | 1395.2 (753.31-2392.97) | -8.91 (-9.13--8.68) |
| Bolivia (Plurinational State of) | 213677 (143937-303765) | 7955.68 (5359.09-11309.85) | 28922 (18192-41855) | 829.53 (521.79-1200.48) | -7.37 (-7.51--7.22) |
| Bosnia and Herzegovina | 1809 (1343-2331) | 165.08 (122.56-212.78) | 407 (299-558) | 82.97 (61.05-113.77) | -1.49 (-1.85--1.13) |
| Botswana | 58898 (45399-75428) | 9975.25 (7688.94-12774.77) | 29790 (19417-42856) | 4266.21 (2780.69-6137.44) | -2.55 (-2.99--2.1) |
| Brazil | 2900765 (2478218-3328417) | 5584.37 (4770.91-6407.65) | 93307 (75993-113142) | 193.64 (157.71-234.81) | -10.55 (-10.71--10.39) |
| Brunei Darussalam | 68 (51-88) | 75.53 (56.72-97.34) | 52 (38-71) | 55.31 (40.41-74.84) | -0.64 (-0.78--0.5) |
| Bulgaria | 2381 (1998-2812) | 137.14 (115.1-161.96) | 1193 (923-1497) | 122.26 (94.54-153.33) | 0.39 (-0.55-1.33) |
| Burkina Faso | 1346057 (905702-1815319) | 28523.84 (19192.43-38467.83) | 705153 (477960-1025195) | 6798.51 (4608.1-9884.1) | -4.54 (-4.83--4.25) |
| Burundi | 417988 (272004-599369) | 15946.89 (10377.38-22866.83) | 210281 (104615-405524) | 3592 (1787.03-6927.11) | -3.95 (-4.5--3.4) |
| Cabo Verde | 15306 (10964-20548) | 9728.7 (6969.03-13061.07) | 1082 (759-1515) | 755.79 (530.37-1058.22) | -8.39 (-9.09--7.68) |
| Cambodia | 519616 (371616-704299) | 11148.31 (7972.98-15110.66) | 34350 (25003-46898) | 671.34 (488.66-916.59) | -9.48 (-9.76--9.21) |
| Cameroon | 696665 (450098-1039147) | 14269.95 (9219.46-21285.1) | 426864 (228139-791311) | 3169.73 (1694.07-5875.97) | -4.18 (-4.9--3.46) |
| Canada | 4228 (2811-6216) | 73.51 (48.88-108.07) | 2500 (1979-3096) | 40.5 (32.06-50.16) | -0.88 (-1.81-0.06) |
| Central African Republic | 358353 (219753-486587) | 29308.94 (17973.12-39796.89) | 251305 (149673-402192) | 11003.68 (6553.63-17610.47) | -2.71 (-2.94--2.49) |
| Chad | 1383830 (903862-1889305) | 47288.37 (30886.86-64561.54) | 1890089 (1282016-3165437) | 20966.29 (14221.09-35113.4) | -2.53 (-2.73--2.33) |
| Chile | 18460 (16411-20734) | 464.76 (413.18-522.01) | 2804 (2275-3468) | 76.79 (62.28-94.97) | -4.94 (-5.14--4.75) |
| China | 7011080 (5307338-8731762) | 2202.14 (1667-2742.59) | 124113 (96767-153731) | 47.8 (37.27-59.21) | -12.26 (-12.59--11.92) |
| Colombia | 336342 (289927-381541) | 2883.84 (2485.87-3271.37) | 27525 (20079-36865) | 259.35 (189.19-347.35) | -8.52 (-9.17--7.86) |
| Comoros | 22104 (13725-34184) | 10392.6 (6452.85-16072.18) | 4644 (2628-7345) | 1933.72 (1094.5-3058.53) | -5.32 (-5.72--4.93) |
| Congo | 151229 (97686-227013) | 14362.12 (9277.19-21559.31) | 37810 (19319-74481) | 1959.8 (1001.34-3860.56) | -6.02 (-6.73--5.31) |
| Cook Islands | 13 (9-18) | 202.83 (140.29-279.12) | 10 (6-15) | 270.29 (169.63-394.87) | 0.62 (0.47-0.77) |
| Costa Rica | 9370 (8231-10418) | 833.45 (732.17-926.73) | 1936 (1397-2606) | 190.26 (137.34-256.19) | -4 (-4.61--3.39) |
| Croatia | 656 (547-779) | 66.51 (55.4-78.93) | 478 (357-614) | 80.11 (59.83-102.84) | 2.07 (0.46-3.71) |
| Cuba | 15059 (13594-16700) | 601.37 (542.89-666.93) | 3121 (2187-4254) | 175.65 (123.05-239.36) | -3.29 (-3.93--2.65) |
| Cyprus | 502 (332-663) | 253.56 (167.76-334.86) | 335 (221-488) | 153 (101.14-222.93) | -0.88 (-1.19--0.56) |
| Czechia | 3286 (2706-3931) | 149.09 (122.77-178.39) | 2961 (2372-3629) | 172.53 (138.18-211.45) | 2.22 (0.54-3.93) |
| C?te d'Ivoire | 792113 (556462-1085157) | 13888.68 (9756.84-19026.83) | 338853 (201885-551035) | 2928.27 (1744.64-4761.88) | -4.22 (-4.68--3.76) |
| Democratic People's Republic of Korea | 16344 (11845-22664) | 274.72 (199.11-380.96) | 10457 (6812-15659) | 219.06 (142.7-328.02) | 0.09 (-0.31-0.5) |
| Democratic Republic of the Congo | 3069148 (2121888-4074175) | 17336.13 (11985.52-23013.04) | 745561 (388949-1312816) | 1962.06 (1023.58-3454.88) | -6.44 (-7.65--5.21) |
| Denmark | 1276 (921-1762) | 144.5 (104.31-199.43) | 1480 (1023-2081) | 155.15 (107.26-218.05) | 0.79 (0.32-1.26) |
| Djibouti | 27753 (18836-39299) | 15939.38 (10818.07-22570.74) | 7474 (4339-12513) | 1808.91 (1050.07-3028.56) | -6.68 (-7.09--6.26) |
| Dominica | 152 (112-198) | 613.35 (452.31-797.25) | 53 (36-75) | 386.18 (262.14-549.05) | -1.3 (-1.75--0.84) |
| Dominican Republic | 199661 (158895-252996) | 7407.33 (5894.91-9386.01) | 26337 (15549-37444) | 896.3 (529.16-1274.28) | -6.62 (-7.08--6.15) |
| Ecuador | 186513 (171196-203740) | 4825.33 (4429.07-5271.01) | 8074 (6055-10510) | 159.22 (119.4-207.26) | -11.1 (-11.56--10.65) |
| Egypt | 2555029 (1871550-3404129) | 11518.62 (8437.35-15346.54) | 300436 (219481-418000) | 815.18 (595.52-1134.17) | -7.49 (-7.89--7.09) |
| El Salvador | 167005 (136101-201985) | 7738.58 (6306.57-9359.46) | 7077 (4888-9766) | 389.12 (268.73-536.98) | -9.9 (-10.57--9.23) |
| Equatorial Guinea | 66035 (38666-93969) | 33534.84 (19635.61-47720.43) | 4925 (2966-7942) | 841.82 (506.99-1357.59) | -13 (-13.55--12.45) |
| Eritrea | 413863 (293373-540968) | 25998.33 (18429.29-33982.92) | 95020 (58420-144566) | 3763.73 (2314.02-5726.22) | -6.12 (-6.28--5.96) |
| Estonia | 698 (578-846) | 199.87 (165.6-242.45) | 167 (109-253) | 77.43 (50.64-117.16) | -2.67 (-3.03--2.31) |
| Eswatini | 68794 (53673-86074) | 17834.34 (13914.29-22314.02) | 19965 (12841-29528) | 4838.33 (3112.05-7155.92) | -3.96 (-4.54--3.38) |
| Ethiopia | 5249041 (2799749-8010053) | 21544.34 (11491.38-32876.73) | 1456383 (1041954-2061778) | 3283.81 (2349.37-4648.83) | -6.31 (-6.49--6.13) |
| Fiji | 3708 (2736-5076) | 1317.46 (972.23-1803.56) | 2036 (1364-2880) | 746.98 (500.59-1056.6) | -1.23 (-1.57--0.89) |
| Finland | 1126 (711-1694) | 116.64 (73.67-175.56) | 628 (392-948) | 74.17 (46.3-111.87) | -1.25 (-2.07--0.42) |
| France | 18157 (12707-24789) | 155 (108.47-211.62) | 24412 (15593-36456) | 210.31 (134.33-314.07) | 1.37 (1.06-1.69) |
| Gabon | 36733 (19347-66925) | 9014.16 (4747.75-16423.11) | 6262 (2900-12723) | 979.73 (453.78-1990.61) | -6.66 (-6.98--6.34) |
| Gambia | 71183 (47778-96481) | 15431.89 (10357.92-20916.24) | 22662 (15343-31001) | 2281.15 (1544.44-3120.57) | -6.15 (-6.5--5.79) |
| Georgia | 19673 (16638-23127) | 1437.31 (1215.6-1689.71) | 506 (386-684) | 68.75 (52.43-92.9) | -9.64 (-10.17--9.1) |
| Germany | 19253 (12637-28173) | 148.72 (97.61-217.62) | 9340 (6955-12590) | 78.06 (58.13-105.22) | -1.23 (-2.16--0.28) |
| Ghana | 745580 (490256-1035034) | 11100.59 (7299.19-15410.12) | 173084 (114652-243726) | 1343.5 (889.94-1891.83) | -6.29 (-6.59--6) |
| Greece | 2298 (1395-3605) | 113.56 (68.94-178.13) | 1313 (755-1980) | 94.11 (54.14-141.93) | 0.19 (-0.49-0.87) |
| Greenland | 21 (11-29) | 144.52 (76.16-206.9) | 6 (4-8) | 50.57 (36.32-69.57) | -2.68 (-2.96--2.39) |
| Grenada | 211 (170-261) | 631.38 (509.39-782.74) | 61 (39-88) | 278.52 (179.59-403.85) | -2.26 (-2.66--1.85) |
| Guam | 123 (95-163) | 296.07 (227.83-390.47) | 125 (88-170) | 342.4 (240.8-465.13) | 0.82 (0.62-1.02) |
| Guatemala | 564274 (509469-630198) | 13894.19 (12544.71-15517.42) | 98507 (76301-126215) | 1996.49 (1546.42-2558.06) | -5.67 (-5.95--5.38) |
| Guinea | 761407 (499456-1050400) | 27669 (18149.88-38170.82) | 155102 (81294-278303) | 2565.47 (1344.64-4603.29) | -7.01 (-7.3--6.71) |
| Guinea-Bissau | 110084 (77507-150804) | 22820.19 (16066.94-31261.21) | 23167 (14181-34891) | 2579.41 (1578.87-3884.79) | -6.87 (-7.74--5.99) |
| Guyana | 16615 (13504-19917) | 5652.48 (4594.4-6775.98) | 1496 (1110-2011) | 701.17 (520.23-942.5) | -6.66 (-7--6.32) |
| Haiti | 845432 (663053-1021265) | 31161.29 (24439.07-37642.19) | 322639 (213705-452262) | 7412.26 (4909.62-10390.21) | -4.15 (-4.66--3.64) |
| Honduras | 209647 (173260-248477) | 9488.75 (7841.84-11246.22) | 32719 (21157-45320) | 998.38 (645.59-1382.89) | -7.25 (-7.47--7.03) |
| Hungary | 3490 (2835-4270) | 163.76 (133.05-200.36) | 2936 (2244-3667) | 211.46 (161.58-264.13) | 1.82 (0.21-3.46) |
| Iceland | 76 (48-113) | 119.3 (75.24-178.51) | 86 (54-129) | 126.61 (79.65-191.71) | 0.67 (0.14-1.2) |
| India | 47778088 (37523642-58094120) | 14632.47 (11491.95-17791.85) | 5065367 (3481226-6979144) | 1382.47 (950.12-1904.79) | -7.29 (-7.62--6.97) |
| Indonesia | 10447569 (7219938-13063112) | 15423.67 (10658.74-19284.97) | 914254 (703887-1156206) | 1358.71 (1046.08-1718.28) | -7.54 (-7.68--7.4) |
| Iran (Islamic Republic of) | 384800 (268056-620670) | 1515.88 (1055.98-2445.07) | 24035 (17444-33006) | 119.11 (86.45-163.56) | -6.67 (-6.99--6.34) |
| Iraq | 224523 (158995-313699) | 2725.97 (1930.38-3808.66) | 58786 (42272-78292) | 436.69 (314.01-581.58) | -6.19 (-6.45--5.94) |
| Ireland | 1124 (692-1733) | 114.36 (70.46-176.37) | 1190 (727-1826) | 119.37 (72.92-183.14) | 2.32 (1.29-3.37) |
| Israel | 2337 (1734-3140) | 152.46 (113.08-204.79) | 2687 (1978-3624) | 102.24 (75.28-137.93) | -0.33 (-1.15-0.49) |
| Italy | 6818 (4278-10332) | 73.87 (46.35-111.94) | 3842 (2995-5078) | 50.55 (39.4-66.82) | -0.67 (-0.94--0.4) |
| Jamaica | 15590 (13540-17994) | 1866.61 (1621.2-2154.42) | 1341 (1023-1718) | 229.7 (175.27-294.18) | -5.57 (-6.82--4.31) |
| Japan | 36599 (24010-52008) | 158.51 (103.99-225.25) | 29273 (19101-42307) | 189.54 (123.67-273.93) | 1.08 (0.7-1.46) |
| Jordan | 10845 (8050-14353) | 663.96 (492.84-878.75) | 6054 (4614-7913) | 166.65 (126.99-217.82) | -4.24 (-4.5--3.98) |
| Kazakhstan | 154360 (135829-172492) | 2970.62 (2614-3319.55) | 4443 (3410-5834) | 81.88 (62.83-107.51) | -12.45 (-13.06--11.84) |
| Kenya | 1617177 (1017121-2090133) | 14477.66 (9105.71-18711.76) | 520662 (397039-668411) | 2789.48 (2127.16-3581.05) | -4.76 (-5.24--4.28) |
| Kiribati | 3104 (2282-4215) | 10511.73 (7727.09-14272.29) | 897 (590-1264) | 2135.98 (1404.15-3009.47) | -4.68 (-4.99--4.38) |
| Kuwait | 1743 (1447-2121) | 314.47 (261.11-382.65) | 969 (651-1390) | 114.57 (76.96-164.46) | -1.46 (-1.97--0.96) |
| Kyrgyzstan | 71283 (60963-81261) | 4249.17 (3633.96-4843.93) | 5827 (4695-7245) | 256.21 (206.44-318.56) | -8.98 (-9.52--8.44) |
| Lao People's Democratic Republic | 494398 (318764-691312) | 26824.13 (17294.88-37507.93) | 40012 (25920-60419) | 1742.4 (1128.74-2631.08) | -8.83 (-9.04--8.62) |
| Latvia | 1043 (849-1274) | 183.3 (149.26-223.96) | 151 (98-222) | 50.85 (33.13-74.69) | -3.77 (-4.17--3.37) |
| Lebanon | 10728 (6500-15179) | 1025.78 (621.45-1451.36) | 4150 (2955-5838) | 324.74 (231.2-456.82) | -3.52 (-3.71--3.32) |
| Lesotho | 132281 (108801-160635) | 19379.23 (15939.35-23533.16) | 60327 (40510-82990) | 9568.25 (6425.24-13162.79) | -2.15 (-2.43--1.87) |
| Liberia | 414402 (297767-534523) | 36666.99 (26346.94-47295.54) | 97357 (54823-175615) | 4453.8 (2508-8033.9) | -6.74 (-7.18--6.29) |
| Libya | 32712 (19625-49840) | 1806.34 (1083.7-2752.19) | 3509 (1907-5106) | 235.24 (127.85-342.3) | -5.66 (-6.33--4.99) |
| Lithuania | 1927 (1578-2308) | 231.93 (190.01-277.84) | 330 (213-472) | 80.88 (52.3-115.78) | -3.69 (-4.33--3.04) |
| Luxembourg | 86 (60-119) | 129.46 (90.24-179.35) | 92 (67-126) | 91.17 (66.5-124.78) | -0.95 (-1.52--0.38) |
| Madagascar | 1448231 (1147461-1774662) | 26543.98 (21031.31-32527) | 883384 (533432-1431861) | 7528.72 (4546.22-12203.16) | -3.48 (-3.82--3.13) |
| Malawi | 1555863 (998343-2042408) | 34197.66 (21943.44-44891.84) | 260112 (165319-377824) | 3201.83 (2034.98-4650.79) | -7.61 (-7.86--7.37) |
| Malaysia | 44487 (32210-56403) | 676.81 (490.03-858.1) | 24860 (17383-34629) | 326.52 (228.31-454.83) | -1.69 (-2.39--0.98) |
| Maldives | 12520 (8438-15929) | 11919.39 (8032.86-15164.4) | 508 (384-674) | 507.54 (383.44-672.4) | -9.23 (-10.22--8.24) |
| Mali | 1046084 (659095-1469280) | 25330.07 (15959.45-35577.43) | 421550 (280774-638527) | 3641.66 (2425.53-5516.06) | -5.93 (-6.3--5.57) |
| Malta | 100 (59-155) | 113.89 (67.98-177.36) | 80 (49-122) | 124.3 (76.38-190.57) | 0.8 (0.36-1.24) |
| Marshall Islands | 318 (195-474) | 1449.98 (890.58-2159.29) | 101 (68-144) | 579.63 (390.78-827.1) | -2.92 (-3.67--2.15) |
| Mauritania | 123923 (77517-188502) | 13406.32 (8386.01-20392.7) | 35423 (20023-62318) | 1911.63 (1080.56-3363.06) | -6.11 (-6.72--5.5) |
| Mauritius | 3472 (3057-3978) | 1051.81 (926.3-1205.34) | 698 (563-850) | 336.31 (271.42-409.65) | -2.32 (-2.7--1.94) |
| Mexico | 2068659 (1872947-2309859) | 6190.58 (5604.9-6912.38) | 86191 (65149-112842) | 268.79 (203.16-351.89) | -9.39 (-9.82--8.97) |
| Micronesia (Federated States of) | 764 (529-1080) | 1664.14 (1152.24-2352.1) | 120 (84-167) | 392.58 (273.72-545.37) | -4.49 (-5.54--3.43) |
| Monaco | 4 (2-6) | 113.07 (70.91-174.21) | 6 (4-9) | 118.68 (71.52-179.39) | 0.43 (0.03-0.84) |
| Mongolia | 19452 (11913-30735) | 2161.3 (1323.68-3415) | 2156 (908-4516) | 198.45 (83.59-415.62) | -6.72 (-7.13--6.31) |
| Montenegro | 85 (62-111) | 52.29 (38.37-68.96) | 22 (15-33) | 20.05 (13.57-29.25) | -2.42 (-2.92--1.93) |
| Morocco | 1018659 (787996-1300253) | 10409.61 (8052.48-13287.21) | 64574 (41194-98506) | 659.47 (420.7-1006) | -8.37 (-8.69--8.05) |
| Mozambique | 1401066 (863707-2115824) | 22582.7 (13921.43-34103.34) | 414100 (250017-671615) | 2902.7 (1752.53-4707.8) | -6.54 (-6.92--6.15) |
| Myanmar | 2466693 (1224170-3987316) | 16693.53 (8284.67-26984.47) | 191342 (133791-264172) | 1225.39 (856.82-1691.81) | -8.44 (-8.63--8.24) |
| Namibia | 77957 (58402-100175) | 12976.96 (9721.79-16675.48) | 34471 (21077-49719) | 4176.1 (2553.49-6023.39) | -3.14 (-3.5--2.78) |
| Nauru | 52 (36-74) | 1225.72 (841.85-1754.34) | 24 (17-33) | 602.35 (428.91-841.11) | -2.33 (-2.89--1.76) |
| Nepal | 1332777 (1012375-1709689) | 15818.97 (12016.06-20292.61) | 73944 (50338-105281) | 801.37 (545.54-1140.98) | -9.38 (-9.58--9.19) |
| Netherlands | 4146 (2583-6169) | 152.13 (94.79-226.36) | 7104 (4469-10590) | 264.86 (166.64-394.85) | 2.39 (1.77-3.01) |
| New Zealand | 934 (694-1308) | 116.76 (86.77-163.49) | 539 (416-687) | 54.87 (42.4-69.93) | -1.15 (-1.81--0.48) |
| Nicaragua | 194160 (157303-249799) | 10661.26 (8637.43-13716.35) | 10301 (6758-14440) | 520.15 (341.23-729.12) | -9.56 (-10.03--9.09) |
| Niger | 2119058 (1568644-2708038) | 52155.65 (38608.49-66652.02) | 992257 (629530-1565769) | 7773.88 (4932.08-12267.08) | -6.56 (-7.04--6.08) |
| Nigeria | 14871856 (10361389-18418487) | 38011.57 (26483.09-47076.55) | 9153604 (6090290-13110736) | 9010.42 (5995.03-12905.66) | -4.36 (-4.87--3.85) |
| Niue | 5 (4-7) | 676 (495.8-909.84) | 4 (2-5) | 943.14 (593.12-1250.12) | -0.81 (-1.47--0.15) |
| North Macedonia | 11742 (9197-15370) | 2228.97 (1745.95-2917.84) | 655 (481-905) | 200.08 (146.86-276.3) | -6.14 (-7.23--5.04) |
| Northern Mariana Islands | 34 (26-45) | 280.11 (209.74-367.15) | 32 (22-45) | 279.76 (194.75-396.85) | -0.03 (-0.18-0.12) |
| Norway | 692 (431-1053) | 86.65 (53.99-131.86) | 521 (335-766) | 56.41 (36.25-82.9) | -1.82 (-2.1--1.54) |
| Oman | 8008 (4627-13149) | 952.95 (550.56-1564.7) | 2243 (1621-3058) | 183.37 (132.55-250.01) | -3.77 (-4.8--2.72) |
| Pakistan | 7497607 (6011930-9289786) | 15226.03 (12208.94-18865.56) | 1724971 (1137819-2536979) | 2018.88 (1331.68-2969.24) | -5.51 (-5.9--5.13) |
| Palau | 58 (38-91) | 1282.42 (828.24-2006.55) | 16 (12-22) | 492.22 (358.45-670.98) | -3.04 (-3.14--2.94) |
| Palestine | 14727 (9923-22233) | 1520.97 (1024.79-2296.11) | 2355 (1685-3198) | 126.13 (90.22-171.28) | -6.76 (-7.19--6.32) |
| Panama | 17756 (15068-20689) | 2129.22 (1806.87-2480.92) | 7214 (5726-9090) | 625.44 (496.46-788.17) | -3.07 (-3.4--2.74) |
| Papua New Guinea | 154938 (106320-227356) | 9113.66 (6253.89-13373.36) | 157476 (98300-244842) | 4020.28 (2509.55-6250.69) | -2.05 (-2.29--1.81) |
| Paraguay | 54982 (42564-70075) | 3293.17 (2549.4-4197.14) | 5751 (3496-9273) | 286.46 (174.1-461.87) | -8.43 (-8.72--8.14) |
| Peru | 382775 (308783-472851) | 4611.24 (3719.88-5696.38) | 29018 (19111-40438) | 304.26 (200.38-423.99) | -8.81 (-9.23--8.38) |
| Philippines | 1616854 (1178351-2086875) | 6412.44 (4673.33-8276.54) | 289282 (227544-373636) | 850.87 (669.28-1098.98) | -6.09 (-6.26--5.92) |
| Poland | 6940 (6035-7981) | 72.47 (63.01-83.34) | 3776 (2975-4656) | 64.16 (50.54-79.11) | 1.85 (0.18-3.56) |
| Portugal | 2678 (2044-3517) | 126.57 (96.62-166.23) | 856 (616-1180) | 62.84 (45.19-86.65) | -1.89 (-2.06--1.73) |
| Puerto Rico | 2351 (1861-2975) | 236.15 (186.93-298.77) | 637 (446-898) | 143.31 (100.38-202.1) | -1.76 (-2--1.53) |
| Qatar | 379 (277-506) | 303.48 (221.16-404.81) | 550 (399-744) | 111.29 (80.76-150.68) | -2.87 (-3.08--2.67) |
| Republic of Korea | 14105 (10918-18387) | 124.05 (96.02-161.71) | 2693 (1839-3619) | 44.34 (30.27-59.58) | -2.82 (-2.95--2.69) |
| Republic of Moldova | 9843 (8198-11676) | 796.44 (663.32-944.74) | 533 (402-691) | 102.14 (77.03-132.28) | -6.15 (-6.56--5.74) |
| Romania | 29787 (25304-34583) | 534.97 (454.45-621.1) | 3855 (2966-4815) | 128.07 (98.54-159.96) | -4.24 (-4.95--3.52) |
| Russian Federation | 144444 (127546-164783) | 416.28 (367.58-474.9) | 18878 (13854-24849) | 72.39 (53.13-95.29) | -4.99 (-5.38--4.6) |
| Rwanda | 587408 (365978-828590) | 17312.82 (10786.57-24421.24) | 122495 (81778-176142) | 2464.48 (1645.28-3543.8) | -7.02 (-7.8--6.23) |
| Saint Kitts and Nevis | 288 (253-327) | 2041.99 (1790.34-2313.67) | 44 (34-59) | 450.08 (344.1-594.93) | -4.57 (-5.29--3.85) |
| Saint Lucia | 531 (435-637) | 1030.53 (843.15-1235.45) | 88 (63-117) | 297.91 (211.77-395.54) | -3.69 (-4.41--2.96) |
| Saint Vincent and the Grenadines | 736 (584-903) | 1791.76 (1422.15-2196.62) | 72 (55-91) | 289.56 (221.37-366.62) | -5.51 (-6.14--4.88) |
| Samoa | 309 (175-446) | 433.21 (246.05-626.27) | 214 (140-295) | 267.21 (175.73-369.01) | -1.36 (-1.55--1.16) |
| San Marino | 4 (3-6) | 105.72 (67.03-158.39) | 5 (3-7) | 104.42 (62.11-163.02) | 0.36 (-0.14-0.86) |
| Sao Tome and Principe | 8293 (6399-10403) | 14635.48 (11292.47-18357.6) | 501 (342-706) | 643.89 (439.12-906.9) | -9.8 (-10.45--9.16) |
| Saudi Arabia | 122002 (82645-184920) | 1861.67 (1261.1-2821.76) | 14666 (9931-19698) | 193.86 (131.27-260.37) | -7.05 (-7.44--6.65) |
| Senegal | 870635 (658334-1093055) | 23844.62 (18030.2-29936.2) | 114211 (76427-162825) | 1795.61 (1201.57-2559.91) | -8.03 (-8.63--7.43) |
| Serbia | 3760 (1831-5528) | 173.39 (84.44-254.9) | 674 (472-957) | 50.74 (35.51-72.05) | -3.43 (-3.71--3.15) |
| Seychelles | 147 (114-190) | 619.43 (481.66-799.2) | 63 (44-85) | 268.57 (188.39-362.02) | -1.76 (-2.27--1.24) |
| Sierra Leone | 507029 (338288-730778) | 27972.05 (18662.88-40315.92) | 148235 (95565-238545) | 4145.04 (2672.24-6670.34) | -6.33 (-6.92--5.73) |
| Singapore | 475 (420-539) | 73.22 (64.66-82.95) | 158 (129-193) | 19.45 (15.84-23.81) | -2.81 (-3.47--2.14) |
| Slovakia | 905 (669-1156) | 68.27 (50.48-87.22) | 541 (347-880) | 63.16 (40.52-102.76) | 0.99 (0.33-1.66) |
| Slovenia | 166 (131-213) | 40.03 (31.63-51.44) | 122 (94-159) | 39.19 (30.11-50.77) | 1.24 (0.6-1.89) |
| Solomon Islands | 5004 (3324-7335) | 3213.69 (2134.5-4711.13) | 1921 (1359-2556) | 738.78 (522.42-982.93) | -4.33 (-4.51--4.15) |
| Somalia | 1152389 (662084-1644550) | 29581.4 (16995.45-42215) | 942304 (560375-1392471) | 9122.52 (5425.04-13480.62) | -3.89 (-4.08--3.69) |
| South Africa | 1747007 (1492602-2016566) | 12832.9 (10964.13-14812.98) | 485354 (382498-620308) | 3192.03 (2515.58-4079.59) | -3.86 (-4.33--3.39) |
| South Sudan | 883176 (553340-1246994) | 33655.65 (21086.4-47519.85) | 676407 (429888-999143) | 15748.89 (10009.15-23263.19) | -2.2 (-2.38--2.02) |
| Spain | 8642 (5474-12815) | 110.28 (69.86-163.54) | 6595 (4346-9282) | 101.77 (67.05-143.22) | 0.3 (-0.17-0.76) |
| Sri Lanka | 99285 (79105-128508) | 1794.4 (1429.68-2322.56) | 12085 (8662-16572) | 236.77 (169.71-324.69) | -6.03 (-6.46--5.6) |
| Sudan | 1407843 (759613-2506726) | 15831.91 (8542.23-28189.41) | 186550 (93804-438917) | 1124.52 (565.45-2645.79) | -8.37 (-8.84--7.91) |
| Suriname | 5453 (4006-7117) | 4185.66 (3074.99-5463.64) | 1305 (871-1836) | 911.2 (608.14-1281.23) | -5 (-5.28--4.73) |
| Sweden | 1155 (709-1774) | 74.77 (45.88-114.84) | 1262 (865-1872) | 69.31 (47.49-102.84) | 0.67 (0.33-1.01) |
| Switzerland | 1796 (1244-2557) | 155.44 (107.68-221.26) | 2056 (1370-2999) | 154.26 (102.78-225.06) | 0.52 (0.12-0.92) |
| Syrian Arab Republic | 78534 (56308-111392) | 1326.11 (950.81-1880.96) | 6374 (4238-8741) | 174.01 (115.7-238.61) | -6.1 (-6.66--5.54) |
| Taiwan (Province of China) | 9684 (8263-11620) | 175.81 (150.01-210.95) | 4860 (3074-7294) | 164.94 (104.33-247.54) | 0.76 (0.1-1.41) |
| Tajikistan | 311355 (252611-381351) | 13408.65 (10878.81-16423.08) | 121902 (83173-169728) | 3400.94 (2320.45-4735.22) | -4.6 (-4.78--4.42) |
| Thailand | 327178 (159321-534314) | 1940.72 (945.04-3169.39) | 31972 (24737-40574) | 327.37 (253.29-415.45) | -5.28 (-5.63--4.93) |
| Timor-Leste | 84660 (47790-115877) | 25451.56 (14367.3-34836.62) | 8458 (5381-12586) | 1624.51 (1033.45-2417.39) | -9.33 (-9.77--8.9) |
| Togo | 381765 (250534-563085) | 21659.96 (14214.42-31947.44) | 142791 (80051-248170) | 4315.16 (2419.15-7499.74) | -4.64 (-5.16--4.12) |
| Tokelau | 10 (6-16) | 1646.2 (972.82-2743.2) | 9 (6-14) | 2364.3 (1504.28-3655.45) | -2.63 (-4.28--0.96) |
| Tonga | 205 (144-282) | 491.3 (343.89-675.23) | 109 (76-150) | 279.49 (193.89-383.8) | -1.35 (-1.52--1.18) |
| Trinidad and Tobago | 3194 (2614-3849) | 786.13 (643.26-947.18) | 534 (395-705) | 195.95 (145.04-258.97) | -4.31 (-4.55--4.07) |
| Tunisia | 32646 (17068-47986) | 1051.24 (549.61-1545.19) | 5729 (3618-7759) | 207.15 (130.81-280.55) | -4.27 (-4.75--3.8) |
| Turkmenistan | 144485 (125136-167682) | 9626.79 (8337.59-11172.36) | 4404 (3361-5749) | 288.95 (220.51-377.24) | -12.55 (-13.16--11.94) |
| Tuvalu | 115 (68-173) | 3305.22 (1958.67-4967.28) | 18 (13-24) | 478.16 (347.4-643.86) | -5.56 (-6.22--4.9) |
| Türkiye | 511062 (359052-776978) | 2494.36 (1752.44-3792.23) | 37984 (28255-49675) | 205.09 (152.56-268.22) | -7.84 (-7.95--7.73) |
| Uganda | 1533595 (711216-2421863) | 18214.53 (8447.12-28764.51) | 433064 (245135-742447) | 2183.25 (1235.82-3742.98) | -6.67 (-6.88--6.45) |
| Ukraine | 26009 (21729-30852) | 228.65 (191.02-271.23) | 4140 (2774-6025) | 65.24 (43.71-94.95) | -4.89 (-5.38--4.39) |
| United Arab Emirates | 2836 (1933-3822) | 481.13 (327.89-648.53) | 2020 (1539-2724) | 150.89 (114.93-203.5) | -1.96 (-2.57--1.33) |
| United Kingdom | 11615 (7057-17813) | 106.36 (64.62-163.12) | 7129 (4483-10461) | 60.5 (38.05-88.78) | -1.17 (-2.34-0.02) |
| United Republic of Tanzania | 2095231 (1508925-2827831) | 17351.06 (12495.74-23417.88) | 686483 (428865-1017776) | 2813.12 (1757.44-4170.71) | -5.69 (-5.99--5.39) |
| United States of America | 38533 (30853-50005) | 68.92 (55.18-89.44) | 10669 (9429-12032) | 17.95 (15.86-20.24) | -3.93 (-4.39--3.47) |
| United States Virgin Islands | 101 (74-136) | 317.5 (232.49-425.84) | 19 (13-27) | 142.14 (95.28-199.63) | -2.39 (-2.46--2.32) |
| Uruguay | 5565 (4939-6232) | 679.83 (603.35-761.3) | 826 (648-1037) | 125.26 (98.22-157.24) | -5.15 (-5.45--4.84) |
| Uzbekistan | 375463 (330962-424003) | 4388.49 (3868.35-4955.84) | 9072 (7055-11994) | 89.91 (69.91-118.86) | -13.09 (-13.92--12.25) |
| Vanuatu | 1869 (1107-2980) | 2744.42 (1626.19-4377.1) | 947 (580-1585) | 812.8 (498.1-1359.81) | -3.73 (-3.93--3.54) |
| Venezuela (Bolivarian Republic of) | 296339 (277640-317154) | 4177.36 (3913.77-4470.78) | 53318 (30621-70996) | 804.93 (462.28-1071.81) | -5.51 (-6.33--4.69) |
| Viet Nam | 366738 (207357-550086) | 1383.27 (782.11-2074.82) | 49684 (33507-70550) | 200.64 (135.31-284.91) | -5.58 (-6.18--4.98) |
| Yemen | 1309632 (875297-1882025) | 18460.19 (12337.92-26528.46) | 138925 (61339-260552) | 1007.54 (444.86-1889.63) | -8.7 (-9.07--8.33) |
| Zambia | 919526 (604076-1271732) | 24491.06 (16089.22-33871.86) | 238345 (160556-341064) | 2881.5 (1941.06-4123.34) | -6.75 (-7.4--6.09) |
| Zimbabwe | 226379 (166489-280725) | 4700.25 (3456.78-5828.64) | 205484 (120375-294283) | 3264.84 (1912.58-4675.72) | -0.35 (-1.32-0.63) |

eTable 4. Risk factors for diarrhea disease in children.

| measure | location | rei | percent | year |
| --- | --- | --- | --- | --- |
| Death cases | Global | Ambient particulate matter pollution | 0.001322702 | 1990 |
| Death cases | Global | Household air pollution from solid fuels | 0.006805085 | 1990 |
| Death cases | Global | No access to handwashing facility | 0.072529461 | 1990 |
| Death cases | Global | Unsafe water source | 0.228795434 | 1990 |
| Death cases | Global | Child wasting | 0.160833382 | 1990 |
| Death cases | Global | Child stunting | 0.092933114 | 1990 |
| Death cases | Global | Short gestation for birth weight | 0.010219015 | 1990 |
| Death cases | Global | Low birth weight for gestation | 0.020937966 | 1990 |
| Death cases | Global | Child underweight | 0.127027813 | 1990 |
| Death cases | Global | Vitamin A deficiency | 0.018096543 | 1990 |
| Death cases | Global | Zinc deficiency | 0.003084139 | 1990 |
| Death cases | Global | Unsafe sanitation | 0.195544567 | 1990 |
| Death cases | Global | Non-exclusive breastfeeding | 0.054199101 | 1990 |
| Death cases | Global | Discontinued breastfeeding | 0.007671679 | 1990 |
| Death cases | High SDI | Ambient particulate matter pollution | 0.004486671 | 1990 |
| Death cases | High SDI | Household air pollution from solid fuels | 0.000340791 | 1990 |
| Death cases | High SDI | No access to handwashing facility | 0.032587942 | 1990 |
| Death cases | High SDI | Unsafe water source | 0.230290456 | 1990 |
| Death cases | High SDI | Child wasting | 0.214006192 | 1990 |
| Death cases | High SDI | Child stunting | 0.082150355 | 1990 |
| Death cases | High SDI | Short gestation for birth weight | 0.001913606 | 1990 |
| Death cases | High SDI | Low birth weight for gestation | 0.0199257 | 1990 |
| Death cases | High SDI | Child underweight | 0.105501223 | 1990 |
| Death cases | High SDI | Vitamin A deficiency | 0.004272504 | 1990 |
| Death cases | High SDI | Zinc deficiency | 0.001399157 | 1990 |
| Death cases | High SDI | Unsafe sanitation | 0.162412731 | 1990 |
| Death cases | High SDI | Non-exclusive breastfeeding | 0.116248873 | 1990 |
| Death cases | High SDI | Discontinued breastfeeding | 0.0244638 | 1990 |
| Death cases | High-middle SDI | Ambient particulate matter pollution | 0.002616052 | 1990 |
| Death cases | High-middle SDI | Household air pollution from solid fuels | 0.002047128 | 1990 |
| Death cases | High-middle SDI | No access to handwashing facility | 0.03792705 | 1990 |
| Death cases | High-middle SDI | Unsafe water source | 0.256372321 | 1990 |
| Death cases | High-middle SDI | Child wasting | 0.176249483 | 1990 |
| Death cases | High-middle SDI | Child stunting | 0.084215641 | 1990 |
| Death cases | High-middle SDI | Short gestation for birth weight | 0.003520424 | 1990 |
| Death cases | High-middle SDI | Low birth weight for gestation | 0.016137871 | 1990 |
| Death cases | High-middle SDI | Child underweight | 0.100310151 | 1990 |
| Death cases | High-middle SDI | Vitamin A deficiency | 0.011539697 | 1990 |
| Death cases | High-middle SDI | Zinc deficiency | 0.000859249 | 1990 |
| Death cases | High-middle SDI | Unsafe sanitation | 0.216406901 | 1990 |
| Death cases | High-middle SDI | Non-exclusive breastfeeding | 0.068814716 | 1990 |
| Death cases | High-middle SDI | Discontinued breastfeeding | 0.022983316 | 1990 |
| Death cases | Low-middle SDI | Ambient particulate matter pollution | 0.001485595 | 1990 |
| Death cases | Low-middle SDI | Household air pollution from solid fuels | 0.008358432 | 1990 |
| Death cases | Low-middle SDI | No access to handwashing facility | 0.070669251 | 1990 |
| Death cases | Low-middle SDI | Unsafe water source | 0.2260979 | 1990 |
| Death cases | Low-middle SDI | Child wasting | 0.157351447 | 1990 |
| Death cases | Low-middle SDI | Child stunting | 0.091651661 | 1990 |
| Death cases | Low-middle SDI | Short gestation for birth weight | 0.013372794 | 1990 |
| Death cases | Low-middle SDI | Low birth weight for gestation | 0.025602057 | 1990 |
| Death cases | Low-middle SDI | Child underweight | 0.128660318 | 1990 |
| Death cases | Low-middle SDI | Vitamin A deficiency | 0.016955515 | 1990 |
| Death cases | Low-middle SDI | Zinc deficiency | 0.003557523 | 1990 |
| Death cases | Low-middle SDI | Unsafe sanitation | 0.194480259 | 1990 |
| Death cases | Low-middle SDI | Non-exclusive breastfeeding | 0.055368228 | 1990 |
| Death cases | Low-middle SDI | Discontinued breastfeeding | 0.006389019 | 1990 |
| Death cases | Low SDI | Ambient particulate matter pollution | 0.00086768 | 1990 |
| Death cases | Low SDI | Household air pollution from solid fuels | 0.005790539 | 1990 |
| Death cases | Low SDI | No access to handwashing facility | 0.085930414 | 1990 |
| Death cases | Low SDI | Unsafe water source | 0.228161872 | 1990 |
| Death cases | Low SDI | Child wasting | 0.159955864 | 1990 |
| Death cases | Low SDI | Child stunting | 0.094579153 | 1990 |
| Death cases | Low SDI | Short gestation for birth weight | 0.00831477 | 1990 |
| Death cases | Low SDI | Low birth weight for gestation | 0.016089367 | 1990 |
| Death cases | Low SDI | Child underweight | 0.129519384 | 1990 |
| Death cases | Low SDI | Vitamin A deficiency | 0.021444852 | 1990 |
| Death cases | Low SDI | Zinc deficiency | 0.003046484 | 1990 |
| Death cases | Low SDI | Unsafe sanitation | 0.194163658 | 1990 |
| Death cases | Low SDI | Non-exclusive breastfeeding | 0.046242704 | 1990 |
| Death cases | Low SDI | Discontinued breastfeeding | 0.00589326 | 1990 |
| Death cases | Middle SDI | Ambient particulate matter pollution | 0.001759193 | 1990 |
| Death cases | Middle SDI | Household air pollution from solid fuels | 0.005059033 | 1990 |
| Death cases | Middle SDI | No access to handwashing facility | 0.050325587 | 1990 |
| Death cases | Middle SDI | Unsafe water source | 0.235526475 | 1990 |
| Death cases | Middle SDI | Child wasting | 0.171471935 | 1990 |
| Death cases | Middle SDI | Child stunting | 0.093859654 | 1990 |
| Death cases | Middle SDI | Short gestation for birth weight | 0.005988706 | 1990 |
| Death cases | Middle SDI | Low birth weight for gestation | 0.018879714 | 1990 |
| Death cases | Middle SDI | Child underweight | 0.119140379 | 1990 |
| Death cases | Middle SDI | Vitamin A deficiency | 0.014379402 | 1990 |
| Death cases | Middle SDI | Zinc deficiency | 0.001989959 | 1990 |
| Death cases | Middle SDI | Unsafe sanitation | 0.200018475 | 1990 |
| Death cases | Middle SDI | Non-exclusive breastfeeding | 0.067561962 | 1990 |
| Death cases | Middle SDI | Discontinued breastfeeding | 0.014039527 | 1990 |
| Death cases | Global | Ambient particulate matter pollution | 0.001428463 | 2021 |
| Death cases | Global | Household air pollution from solid fuels | 0.004585787 | 2021 |
| Death cases | Global | No access to handwashing facility | 0.084564515 | 2021 |
| Death cases | Global | Unsafe water source | 0.252779296 | 2021 |
| Death cases | Global | Child wasting | 0.16393413 | 2021 |
| Death cases | Global | Child stunting | 0.082126915 | 2021 |
| Death cases | Global | Short gestation for birth weight | 0.007193761 | 2021 |
| Death cases | Global | Low birth weight for gestation | 0.015265372 | 2021 |
| Death cases | Global | Child underweight | 0.11874067 | 2021 |
| Death cases | Global | Vitamin A deficiency | 0.010227872 | 2021 |
| Death cases | Global | Zinc deficiency | 0.001478061 | 2021 |
| Death cases | Global | Unsafe sanitation | 0.199772258 | 2021 |
| Death cases | Global | Non-exclusive breastfeeding | 0.050231938 | 2021 |
| Death cases | Global | Discontinued breastfeeding | 0.007670964 | 2021 |
| Death cases | High SDI | Ambient particulate matter pollution | 0.009091441 | 2021 |
| Death cases | High SDI | Household air pollution from solid fuels | 4.07E-05 | 2021 |
| Death cases | High SDI | No access to handwashing facility | 0.015939999 | 2021 |
| Death cases | High SDI | Unsafe water source | 0.153598303 | 2021 |
| Death cases | High SDI | Child wasting | 0.239610577 | 2021 |
| Death cases | High SDI | Child stunting | 0.06058846 | 2021 |
| Death cases | High SDI | Short gestation for birth weight | 0.016308178 | 2021 |
| Death cases | High SDI | Low birth weight for gestation | 0.078167971 | 2021 |
| Death cases | High SDI | Child underweight | 0.078901308 | 2021 |
| Death cases | High SDI | Vitamin A deficiency | 0.00225403 | 2021 |
| Death cases | High SDI | Zinc deficiency | 0.001101518 | 2021 |
| Death cases | High SDI | Unsafe sanitation | 0.051098306 | 2021 |
| Death cases | High SDI | Non-exclusive breastfeeding | 0.24442229 | 2021 |
| Death cases | High SDI | Discontinued breastfeeding | 0.048876913 | 2021 |
| Death cases | High-middle SDI | Ambient particulate matter pollution | 0.007025386 | 2021 |
| Death cases | High-middle SDI | Household air pollution from solid fuels | 0.00013909 | 2021 |
| Death cases | High-middle SDI | No access to handwashing facility | 0.023061095 | 2021 |
| Death cases | High-middle SDI | Unsafe water source | 0.28032207 | 2021 |
| Death cases | High-middle SDI | Child wasting | 0.199781542 | 2021 |
| Death cases | High-middle SDI | Child stunting | 0.080534596 | 2021 |
| Death cases | High-middle SDI | Short gestation for birth weight | 0.013639513 | 2021 |
| Death cases | High-middle SDI | Low birth weight for gestation | 0.039650961 | 2021 |
| Death cases | High-middle SDI | Child underweight | 0.104364284 | 2021 |
| Death cases | High-middle SDI | Vitamin A deficiency | 0.004248369 | 2021 |
| Death cases | High-middle SDI | Zinc deficiency | 0.000837125 | 2021 |
| Death cases | High-middle SDI | Unsafe sanitation | 0.107174987 | 2021 |
| Death cases | High-middle SDI | Non-exclusive breastfeeding | 0.116581176 | 2021 |
| Death cases | High-middle SDI | Discontinued breastfeeding | 0.022639806 | 2021 |
| Death cases | Low-middle SDI | Ambient particulate matter pollution | 0.002455451 | 2021 |
| Death cases | Low-middle SDI | Household air pollution from solid fuels | 0.005510267 | 2021 |
| Death cases | Low-middle SDI | No access to handwashing facility | 0.0708125 | 2021 |
| Death cases | Low-middle SDI | Unsafe water source | 0.264222369 | 2021 |
| Death cases | Low-middle SDI | Child wasting | 0.161528641 | 2021 |
| Death cases | Low-middle SDI | Child stunting | 0.077650187 | 2021 |
| Death cases | Low-middle SDI | Short gestation for birth weight | 0.010512685 | 2021 |
| Death cases | Low-middle SDI | Low birth weight for gestation | 0.021458042 | 2021 |
| Death cases | Low-middle SDI | Child underweight | 0.114185681 | 2021 |
| Death cases | Low-middle SDI | Vitamin A deficiency | 0.008709348 | 2021 |
| Death cases | Low-middle SDI | Zinc deficiency | 0.001812539 | 2021 |
| Death cases | Low-middle SDI | Unsafe sanitation | 0.195015191 | 2021 |
| Death cases | Low-middle SDI | Non-exclusive breastfeeding | 0.05726351 | 2021 |
| Death cases | Low-middle SDI | Discontinued breastfeeding | 0.00886359 | 2021 |
| Death cases | Low SDI | Ambient particulate matter pollution | 0.000847841 | 2021 |
| Death cases | Low SDI | Household air pollution from solid fuels | 0.0045268 | 2021 |
| Death cases | Low SDI | No access to handwashing facility | 0.092171243 | 2021 |
| Death cases | Low SDI | Unsafe water source | 0.247202781 | 2021 |
| Death cases | Low SDI | Child wasting | 0.162391539 | 2021 |
| Death cases | Low SDI | Child stunting | 0.083606447 | 2021 |
| Death cases | Low SDI | Short gestation for birth weight | 0.005946117 | 2021 |
| Death cases | Low SDI | Low birth weight for gestation | 0.012572227 | 2021 |
| Death cases | Low SDI | Child underweight | 0.120646495 | 2021 |
| Death cases | Low SDI | Vitamin A deficiency | 0.011083705 | 2021 |
| Death cases | Low SDI | Zinc deficiency | 0.00139722 | 2021 |
| Death cases | Low SDI | Unsafe sanitation | 0.206145279 | 2021 |
| Death cases | Low SDI | Non-exclusive breastfeeding | 0.04505083 | 2021 |
| Death cases | Low SDI | Discontinued breastfeeding | 0.006411475 | 2021 |
| Death cases | Middle SDI | Ambient particulate matter pollution | 0.003722082 | 2021 |
| Death cases | Middle SDI | Household air pollution from solid fuels | 0.001810775 | 2021 |
| Death cases | Middle SDI | No access to handwashing facility | 0.054974487 | 2021 |
| Death cases | Middle SDI | Unsafe water source | 0.27105978 | 2021 |
| Death cases | Middle SDI | Child wasting | 0.18910491 | 2021 |
| Death cases | Middle SDI | Child stunting | 0.083181877 | 2021 |
| Death cases | Middle SDI | Short gestation for birth weight | 0.007958873 | 2021 |
| Death cases | Middle SDI | Low birth weight for gestation | 0.019974178 | 2021 |
| Death cases | Middle SDI | Child underweight | 0.116070565 | 2021 |
| Death cases | Middle SDI | Vitamin A deficiency | 0.006727443 | 2021 |
| Death cases | Middle SDI | Zinc deficiency | 0.001095028 | 2021 |
| Death cases | Middle SDI | Unsafe sanitation | 0.150634804 | 2021 |
| Death cases | Middle SDI | Non-exclusive breastfeeding | 0.077294502 | 2021 |
| Death cases | Middle SDI | Discontinued breastfeeding | 0.016390697 | 2021 |
| Deaths rate | Global | Ambient particulate matter pollution | 0.001322702 | 1990 |
| Deaths rate | Global | Household air pollution from solid fuels | 0.006805086 | 1990 |
| Deaths rate | Global | No access to handwashing facility | 0.072529461 | 1990 |
| Deaths rate | Global | Unsafe water source | 0.228795434 | 1990 |
| Deaths rate | Global | Child wasting | 0.160833381 | 1990 |
| Deaths rate | Global | Child stunting | 0.092933114 | 1990 |
| Deaths rate | Global | Short gestation for birth weight | 0.010219015 | 1990 |
| Deaths rate | Global | Low birth weight for gestation | 0.020937966 | 1990 |
| Deaths rate | Global | Child underweight | 0.127027813 | 1990 |
| Deaths rate | Global | Vitamin A deficiency | 0.018096543 | 1990 |
| Deaths rate | Global | Zinc deficiency | 0.003084139 | 1990 |
| Deaths rate | Global | Unsafe sanitation | 0.195544567 | 1990 |
| Deaths rate | Global | Non-exclusive breastfeeding | 0.054199101 | 1990 |
| Deaths rate | Global | Discontinued breastfeeding | 0.007671679 | 1990 |
| Deaths rate | High SDI | Ambient particulate matter pollution | 0.004486671 | 1990 |
| Deaths rate | High SDI | Household air pollution from solid fuels | 0.00034079 | 1990 |
| Deaths rate | High SDI | No access to handwashing facility | 0.032587942 | 1990 |
| Deaths rate | High SDI | Unsafe water source | 0.230290456 | 1990 |
| Deaths rate | High SDI | Child wasting | 0.214006192 | 1990 |
| Deaths rate | High SDI | Child stunting | 0.082150356 | 1990 |
| Deaths rate | High SDI | Short gestation for birth weight | 0.001913606 | 1990 |
| Deaths rate | High SDI | Low birth weight for gestation | 0.0199257 | 1990 |
| Deaths rate | High SDI | Child underweight | 0.105501223 | 1990 |
| Deaths rate | High SDI | Vitamin A deficiency | 0.004272504 | 1990 |
| Deaths rate | High SDI | Zinc deficiency | 0.001399156 | 1990 |
| Deaths rate | High SDI | Unsafe sanitation | 0.162412731 | 1990 |
| Deaths rate | High SDI | Non-exclusive breastfeeding | 0.116248873 | 1990 |
| Deaths rate | High SDI | Discontinued breastfeeding | 0.0244638 | 1990 |
| Deaths rate | High-middle SDI | Ambient particulate matter pollution | 0.002616052 | 1990 |
| Deaths rate | High-middle SDI | Household air pollution from solid fuels | 0.002047128 | 1990 |
| Deaths rate | High-middle SDI | No access to handwashing facility | 0.03792705 | 1990 |
| Deaths rate | High-middle SDI | Unsafe water source | 0.256372321 | 1990 |
| Deaths rate | High-middle SDI | Child wasting | 0.176249483 | 1990 |
| Deaths rate | High-middle SDI | Child stunting | 0.084215641 | 1990 |
| Deaths rate | High-middle SDI | Short gestation for birth weight | 0.003520424 | 1990 |
| Deaths rate | High-middle SDI | Low birth weight for gestation | 0.016137871 | 1990 |
| Deaths rate | High-middle SDI | Child underweight | 0.100310151 | 1990 |
| Deaths rate | High-middle SDI | Vitamin A deficiency | 0.011539697 | 1990 |
| Deaths rate | High-middle SDI | Zinc deficiency | 0.000859249 | 1990 |
| Deaths rate | High-middle SDI | Unsafe sanitation | 0.216406901 | 1990 |
| Deaths rate | High-middle SDI | Non-exclusive breastfeeding | 0.068814716 | 1990 |
| Deaths rate | High-middle SDI | Discontinued breastfeeding | 0.022983316 | 1990 |
| Deaths rate | Low-middle SDI | Ambient particulate matter pollution | 0.001485595 | 1990 |
| Deaths rate | Low-middle SDI | Household air pollution from solid fuels | 0.008358432 | 1990 |
| Deaths rate | Low-middle SDI | No access to handwashing facility | 0.070669251 | 1990 |
| Deaths rate | Low-middle SDI | Unsafe water source | 0.2260979 | 1990 |
| Deaths rate | Low-middle SDI | Child wasting | 0.157351447 | 1990 |
| Deaths rate | Low-middle SDI | Child stunting | 0.091651661 | 1990 |
| Deaths rate | Low-middle SDI | Short gestation for birth weight | 0.013372794 | 1990 |
| Deaths rate | Low-middle SDI | Low birth weight for gestation | 0.025602057 | 1990 |
| Deaths rate | Low-middle SDI | Child underweight | 0.128660318 | 1990 |
| Deaths rate | Low-middle SDI | Vitamin A deficiency | 0.016955515 | 1990 |
| Deaths rate | Low-middle SDI | Zinc deficiency | 0.003557523 | 1990 |
| Deaths rate | Low-middle SDI | Unsafe sanitation | 0.194480259 | 1990 |
| Deaths rate | Low-middle SDI | Non-exclusive breastfeeding | 0.055368228 | 1990 |
| Deaths rate | Low-middle SDI | Discontinued breastfeeding | 0.006389019 | 1990 |
| Deaths rate | Low SDI | Ambient particulate matter pollution | 0.00086768 | 1990 |
| Deaths rate | Low SDI | Household air pollution from solid fuels | 0.005790539 | 1990 |
| Deaths rate | Low SDI | No access to handwashing facility | 0.085930414 | 1990 |
| Deaths rate | Low SDI | Unsafe water source | 0.228161871 | 1990 |
| Deaths rate | Low SDI | Child wasting | 0.159955864 | 1990 |
| Deaths rate | Low SDI | Child stunting | 0.094579153 | 1990 |
| Deaths rate | Low SDI | Short gestation for birth weight | 0.00831477 | 1990 |
| Deaths rate | Low SDI | Low birth weight for gestation | 0.016089367 | 1990 |
| Deaths rate | Low SDI | Child underweight | 0.129519384 | 1990 |
| Deaths rate | Low SDI | Vitamin A deficiency | 0.021444852 | 1990 |
| Deaths rate | Low SDI | Zinc deficiency | 0.003046484 | 1990 |
| Deaths rate | Low SDI | Unsafe sanitation | 0.194163658 | 1990 |
| Deaths rate | Low SDI | Non-exclusive breastfeeding | 0.046242704 | 1990 |
| Deaths rate | Low SDI | Discontinued breastfeeding | 0.00589326 | 1990 |
| Deaths rate | Middle SDI | Ambient particulate matter pollution | 0.001759193 | 1990 |
| Deaths rate | Middle SDI | Household air pollution from solid fuels | 0.005059033 | 1990 |
| Deaths rate | Middle SDI | No access to handwashing facility | 0.050325587 | 1990 |
| Deaths rate | Middle SDI | Unsafe water source | 0.235526475 | 1990 |
| Deaths rate | Middle SDI | Child wasting | 0.171471935 | 1990 |
| Deaths rate | Middle SDI | Child stunting | 0.093859654 | 1990 |
| Deaths rate | Middle SDI | Short gestation for birth weight | 0.005988706 | 1990 |
| Deaths rate | Middle SDI | Low birth weight for gestation | 0.018879714 | 1990 |
| Deaths rate | Middle SDI | Child underweight | 0.119140379 | 1990 |
| Deaths rate | Middle SDI | Vitamin A deficiency | 0.014379402 | 1990 |
| Deaths rate | Middle SDI | Zinc deficiency | 0.001989959 | 1990 |
| Deaths rate | Middle SDI | Unsafe sanitation | 0.200018475 | 1990 |
| Deaths rate | Middle SDI | Non-exclusive breastfeeding | 0.067561962 | 1990 |
| Deaths rate | Middle SDI | Discontinued breastfeeding | 0.014039527 | 1990 |
| Deaths rate | Global | Ambient particulate matter pollution | 0.001428463 | 2021 |
| Deaths rate | Global | Household air pollution from solid fuels | 0.004585787 | 2021 |
| Deaths rate | Global | No access to handwashing facility | 0.084564515 | 2021 |
| Deaths rate | Global | Unsafe water source | 0.252779296 | 2021 |
| Deaths rate | Global | Child wasting | 0.16393413 | 2021 |
| Deaths rate | Global | Child stunting | 0.082126915 | 2021 |
| Deaths rate | Global | Short gestation for birth weight | 0.007193761 | 2021 |
| Deaths rate | Global | Low birth weight for gestation | 0.015265372 | 2021 |
| Deaths rate | Global | Child underweight | 0.11874067 | 2021 |
| Deaths rate | Global | Vitamin A deficiency | 0.010227872 | 2021 |
| Deaths rate | Global | Zinc deficiency | 0.001478061 | 2021 |
| Deaths rate | Global | Unsafe sanitation | 0.199772258 | 2021 |
| Deaths rate | Global | Non-exclusive breastfeeding | 0.050231938 | 2021 |
| Deaths rate | Global | Discontinued breastfeeding | 0.007670964 | 2021 |
| Deaths rate | High SDI | Ambient particulate matter pollution | 0.009091442 | 2021 |
| Deaths rate | High SDI | Household air pollution from solid fuels | 4.07E-05 | 2021 |
| Deaths rate | High SDI | No access to handwashing facility | 0.015940001 | 2021 |
| Deaths rate | High SDI | Unsafe water source | 0.153598307 | 2021 |
| Deaths rate | High SDI | Child wasting | 0.239610584 | 2021 |
| Deaths rate | High SDI | Child stunting | 0.060588462 | 2021 |
| Deaths rate | High SDI | Short gestation for birth weight | 0.016308179 | 2021 |
| Deaths rate | High SDI | Low birth weight for gestation | 0.078167974 | 2021 |
| Deaths rate | High SDI | Child underweight | 0.078901309 | 2021 |
| Deaths rate | High SDI | Vitamin A deficiency | 0.00225403 | 2021 |
| Deaths rate | High SDI | Zinc deficiency | 0.00110152 | 2021 |
| Deaths rate | High SDI | Unsafe sanitation | 0.051098307 | 2021 |
| Deaths rate | High SDI | Non-exclusive breastfeeding | 0.244422296 | 2021 |
| Deaths rate | High SDI | Discontinued breastfeeding | 0.048876913 | 2021 |
| Deaths rate | High-middle SDI | Ambient particulate matter pollution | 0.007025386 | 2021 |
| Deaths rate | High-middle SDI | Household air pollution from solid fuels | 0.00013909 | 2021 |
| Deaths rate | High-middle SDI | No access to handwashing facility | 0.023061096 | 2021 |
| Deaths rate | High-middle SDI | Unsafe water source | 0.28032207 | 2021 |
| Deaths rate | High-middle SDI | Child wasting | 0.199781542 | 2021 |
| Deaths rate | High-middle SDI | Child stunting | 0.080534596 | 2021 |
| Deaths rate | High-middle SDI | Short gestation for birth weight | 0.013639513 | 2021 |
| Deaths rate | High-middle SDI | Low birth weight for gestation | 0.039650961 | 2021 |
| Deaths rate | High-middle SDI | Child underweight | 0.104364284 | 2021 |
| Deaths rate | High-middle SDI | Vitamin A deficiency | 0.004248369 | 2021 |
| Deaths rate | High-middle SDI | Zinc deficiency | 0.000837125 | 2021 |
| Deaths rate | High-middle SDI | Unsafe sanitation | 0.107174987 | 2021 |
| Deaths rate | High-middle SDI | Non-exclusive breastfeeding | 0.116581176 | 2021 |
| Deaths rate | High-middle SDI | Discontinued breastfeeding | 0.022639806 | 2021 |
| Deaths rate | Low-middle SDI | Ambient particulate matter pollution | 0.002455451 | 2021 |
| Deaths rate | Low-middle SDI | Household air pollution from solid fuels | 0.005510267 | 2021 |
| Deaths rate | Low-middle SDI | No access to handwashing facility | 0.0708125 | 2021 |
| Deaths rate | Low-middle SDI | Unsafe water source | 0.264222369 | 2021 |
| Deaths rate | Low-middle SDI | Child wasting | 0.161528641 | 2021 |
| Deaths rate | Low-middle SDI | Child stunting | 0.077650187 | 2021 |
| Deaths rate | Low-middle SDI | Short gestation for birth weight | 0.010512685 | 2021 |
| Deaths rate | Low-middle SDI | Low birth weight for gestation | 0.021458042 | 2021 |
| Deaths rate | Low-middle SDI | Child underweight | 0.114185681 | 2021 |
| Deaths rate | Low-middle SDI | Vitamin A deficiency | 0.008709348 | 2021 |
| Deaths rate | Low-middle SDI | Zinc deficiency | 0.001812539 | 2021 |
| Deaths rate | Low-middle SDI | Unsafe sanitation | 0.195015191 | 2021 |
| Deaths rate | Low-middle SDI | Non-exclusive breastfeeding | 0.05726351 | 2021 |
| Deaths rate | Low-middle SDI | Discontinued breastfeeding | 0.00886359 | 2021 |
| Deaths rate | Low SDI | Ambient particulate matter pollution | 0.000847841 | 2021 |
| Deaths rate | Low SDI | Household air pollution from solid fuels | 0.0045268 | 2021 |
| Deaths rate | Low SDI | No access to handwashing facility | 0.092171243 | 2021 |
| Deaths rate | Low SDI | Unsafe water source | 0.247202781 | 2021 |
| Deaths rate | Low SDI | Child wasting | 0.162391539 | 2021 |
| Deaths rate | Low SDI | Child stunting | 0.083606447 | 2021 |
| Deaths rate | Low SDI | Short gestation for birth weight | 0.005946117 | 2021 |
| Deaths rate | Low SDI | Low birth weight for gestation | 0.012572227 | 2021 |
| Deaths rate | Low SDI | Child underweight | 0.120646495 | 2021 |
| Deaths rate | Low SDI | Vitamin A deficiency | 0.011083705 | 2021 |
| Deaths rate | Low SDI | Zinc deficiency | 0.00139722 | 2021 |
| Deaths rate | Low SDI | Unsafe sanitation | 0.206145279 | 2021 |
| Deaths rate | Low SDI | Non-exclusive breastfeeding | 0.04505083 | 2021 |
| Deaths rate | Low SDI | Discontinued breastfeeding | 0.006411475 | 2021 |
| Deaths rate | Middle SDI | Ambient particulate matter pollution | 0.003722082 | 2021 |
| Deaths rate | Middle SDI | Household air pollution from solid fuels | 0.001810775 | 2021 |
| Deaths rate | Middle SDI | No access to handwashing facility | 0.054974487 | 2021 |
| Deaths rate | Middle SDI | Unsafe water source | 0.27105978 | 2021 |
| Deaths rate | Middle SDI | Child wasting | 0.18910491 | 2021 |
| Deaths rate | Middle SDI | Child stunting | 0.083181877 | 2021 |
| Deaths rate | Middle SDI | Short gestation for birth weight | 0.007958873 | 2021 |
| Deaths rate | Middle SDI | Low birth weight for gestation | 0.019974178 | 2021 |
| Deaths rate | Middle SDI | Child underweight | 0.116070565 | 2021 |
| Deaths rate | Middle SDI | Vitamin A deficiency | 0.006727443 | 2021 |
| Deaths rate | Middle SDI | Zinc deficiency | 0.001095028 | 2021 |
| Deaths rate | Middle SDI | Unsafe sanitation | 0.150634804 | 2021 |
| Deaths rate | Middle SDI | Non-exclusive breastfeeding | 0.077294502 | 2021 |
| Deaths rate | Middle SDI | Discontinued breastfeeding | 0.016390697 | 2021 |
| DALYs cases | Global | Ambient particulate matter pollution | 0.001324079 | 1990 |
| DALYs cases | Global | Household air pollution from solid fuels | 0.006807494 | 1990 |
| DALYs cases | Global | No access to handwashing facility | 0.072940955 | 1990 |
| DALYs cases | Global | Unsafe water source | 0.230738543 | 1990 |
| DALYs cases | Global | Child wasting | 0.158870022 | 1990 |
| DALYs cases | Global | Child stunting | 0.091839474 | 1990 |
| DALYs cases | Global | Short gestation for birth weight | 0.010201649 | 1990 |
| DALYs cases | Global | Low birth weight for gestation | 0.020902384 | 1990 |
| DALYs cases | Global | Child underweight | 0.125997 | 1990 |
| DALYs cases | Global | Vitamin A deficiency | 0.018088258 | 1990 |
| DALYs cases | Global | Zinc deficiency | 0.003087874 | 1990 |
| DALYs cases | Global | Unsafe sanitation | 0.197186444 | 1990 |
| DALYs cases | Global | Non-exclusive breastfeeding | 0.054311198 | 1990 |
| DALYs cases | Global | Discontinued breastfeeding | 0.007704629 | 1990 |
| DALYs cases | High SDI | Ambient particulate matter pollution | 0.003998689 | 1990 |
| DALYs cases | High SDI | Household air pollution from solid fuels | 0.00031037 | 1990 |
| DALYs cases | High SDI | No access to handwashing facility | 0.035775724 | 1990 |
| DALYs cases | High SDI | Unsafe water source | 0.254073596 | 1990 |
| DALYs cases | High SDI | Child wasting | 0.186662365 | 1990 |
| DALYs cases | High SDI | Child stunting | 0.075299882 | 1990 |
| DALYs cases | High SDI | Short gestation for birth weight | 0.001647693 | 1990 |
| DALYs cases | High SDI | Low birth weight for gestation | 0.01715687 | 1990 |
| DALYs cases | High SDI | Child underweight | 0.101342867 | 1990 |
| DALYs cases | High SDI | Vitamin A deficiency | 0.004519527 | 1990 |
| DALYs cases | High SDI | Zinc deficiency | 0.001946961 | 1990 |
| DALYs cases | High SDI | Unsafe sanitation | 0.178927622 | 1990 |
| DALYs cases | High SDI | Non-exclusive breastfeeding | 0.109142617 | 1990 |
| DALYs cases | High SDI | Discontinued breastfeeding | 0.029195217 | 1990 |
| DALYs cases | High-middle SDI | Ambient particulate matter pollution | 0.002540767 | 1990 |
| DALYs cases | High-middle SDI | Household air pollution from solid fuels | 0.001999741 | 1990 |
| DALYs cases | High-middle SDI | No access to handwashing facility | 0.038909278 | 1990 |
| DALYs cases | High-middle SDI | Unsafe water source | 0.264049171 | 1990 |
| DALYs cases | High-middle SDI | Child wasting | 0.168128373 | 1990 |
| DALYs cases | High-middle SDI | Child stunting | 0.081230988 | 1990 |
| DALYs cases | High-middle SDI | Short gestation for birth weight | 0.003376259 | 1990 |
| DALYs cases | High-middle SDI | Low birth weight for gestation | 0.015477029 | 1990 |
| DALYs cases | High-middle SDI | Child underweight | 0.097707162 | 1990 |
| DALYs cases | High-middle SDI | Vitamin A deficiency | 0.011546582 | 1990 |
| DALYs cases | High-middle SDI | Zinc deficiency | 0.000912683 | 1990 |
| DALYs cases | High-middle SDI | Unsafe sanitation | 0.223246253 | 1990 |
| DALYs cases | High-middle SDI | Non-exclusive breastfeeding | 0.067922903 | 1990 |
| DALYs cases | High-middle SDI | Discontinued breastfeeding | 0.022952812 | 1990 |
| DALYs cases | Low-middle SDI | Ambient particulate matter pollution | 0.001487461 | 1990 |
| DALYs cases | Low-middle SDI | Household air pollution from solid fuels | 0.008367646 | 1990 |
| DALYs cases | Low-middle SDI | No access to handwashing facility | 0.071151964 | 1990 |
| DALYs cases | Low-middle SDI | Unsafe water source | 0.227677648 | 1990 |
| DALYs cases | Low-middle SDI | Child wasting | 0.155686254 | 1990 |
| DALYs cases | Low-middle SDI | Child stunting | 0.090689289 | 1990 |
| DALYs cases | Low-middle SDI | Short gestation for birth weight | 0.013367421 | 1990 |
| DALYs cases | Low-middle SDI | Low birth weight for gestation | 0.025591772 | 1990 |
| DALYs cases | Low-middle SDI | Child underweight | 0.127762162 | 1990 |
| DALYs cases | Low-middle SDI | Vitamin A deficiency | 0.016969693 | 1990 |
| DALYs cases | Low-middle SDI | Zinc deficiency | 0.003559645 | 1990 |
| DALYs cases | Low-middle SDI | Unsafe sanitation | 0.1958312 | 1990 |
| DALYs cases | Low-middle SDI | Non-exclusive breastfeeding | 0.055461909 | 1990 |
| DALYs cases | Low-middle SDI | Discontinued breastfeeding | 0.006395936 | 1990 |
| DALYs cases | Low SDI | Ambient particulate matter pollution | 0.000873181 | 1990 |
| DALYs cases | Low SDI | Household air pollution from solid fuels | 0.0058286 | 1990 |
| DALYs cases | Low SDI | No access to handwashing facility | 0.086338314 | 1990 |
| DALYs cases | Low SDI | Unsafe water source | 0.229231694 | 1990 |
| DALYs cases | Low SDI | Child wasting | 0.15868405 | 1990 |
| DALYs cases | Low SDI | Child stunting | 0.093750012 | 1990 |
| DALYs cases | Low SDI | Short gestation for birth weight | 0.008353114 | 1990 |
| DALYs cases | Low SDI | Low birth weight for gestation | 0.016163559 | 1990 |
| DALYs cases | Low SDI | Child underweight | 0.128773411 | 1990 |
| DALYs cases | Low SDI | Vitamin A deficiency | 0.021443968 | 1990 |
| DALYs cases | Low SDI | Zinc deficiency | 0.003036315 | 1990 |
| DALYs cases | Low SDI | Unsafe sanitation | 0.195084886 | 1990 |
| DALYs cases | Low SDI | Non-exclusive breastfeeding | 0.046541018 | 1990 |
| DALYs cases | Low SDI | Discontinued breastfeeding | 0.005897878 | 1990 |
| DALYs cases | Middle SDI | Ambient particulate matter pollution | 0.001740737 | 1990 |
| DALYs cases | Middle SDI | Household air pollution from solid fuels | 0.005003997 | 1990 |
| DALYs cases | Middle SDI | No access to handwashing facility | 0.051121181 | 1990 |
| DALYs cases | Middle SDI | Unsafe water source | 0.239604929 | 1990 |
| DALYs cases | Middle SDI | Child wasting | 0.167548762 | 1990 |
| DALYs cases | Middle SDI | Child stunting | 0.092050035 | 1990 |
| DALYs cases | Middle SDI | Short gestation for birth weight | 0.005897786 | 1990 |
| DALYs cases | Middle SDI | Low birth weight for gestation | 0.018593107 | 1990 |
| DALYs cases | Middle SDI | Child underweight | 0.117518745 | 1990 |
| DALYs cases | Middle SDI | Vitamin A deficiency | 0.014417113 | 1990 |
| DALYs cases | Middle SDI | Zinc deficiency | 0.002048568 | 1990 |
| DALYs cases | Middle SDI | Unsafe sanitation | 0.203408617 | 1990 |
| DALYs cases | Middle SDI | Non-exclusive breastfeeding | 0.067045083 | 1990 |
| DALYs cases | Middle SDI | Discontinued breastfeeding | 0.014001336 | 1990 |
| DALYs cases | Global | Ambient particulate matter pollution | 0.001406152 | 2021 |
| DALYs cases | Global | Household air pollution from solid fuels | 0.004499106 | 2021 |
| DALYs cases | Global | No access to handwashing facility | 0.085764758 | 2021 |
| DALYs cases | Global | Unsafe water source | 0.260453783 | 2021 |
| DALYs cases | Global | Child wasting | 0.158270975 | 2021 |
| DALYs cases | Global | Child stunting | 0.079348541 | 2021 |
| DALYs cases | Global | Short gestation for birth weight | 0.007017758 | 2021 |
| DALYs cases | Global | Low birth weight for gestation | 0.01489189 | 2021 |
| DALYs cases | Global | Child underweight | 0.115278736 | 2021 |
| DALYs cases | Global | Vitamin A deficiency | 0.010044106 | 2021 |
| DALYs cases | Global | Zinc deficiency | 0.001476172 | 2021 |
| DALYs cases | Global | Unsafe sanitation | 0.204525498 | 2021 |
| DALYs cases | Global | Non-exclusive breastfeeding | 0.049463868 | 2021 |
| DALYs cases | Global | Discontinued breastfeeding | 0.007558656 | 2021 |
| DALYs cases | High SDI | Ambient particulate matter pollution | 0.006234526 | 2021 |
| DALYs cases | High SDI | Household air pollution from solid fuels | 2.73E-05 | 2021 |
| DALYs cases | High SDI | No access to handwashing facility | 0.034174067 | 2021 |
| DALYs cases | High SDI | Unsafe water source | 0.261367806 | 2021 |
| DALYs cases | High SDI | Child wasting | 0.167814218 | 2021 |
| DALYs cases | High SDI | Child stunting | 0.051716144 | 2021 |
| DALYs cases | High SDI | Short gestation for birth weight | 0.010710543 | 2021 |
| DALYs cases | High SDI | Low birth weight for gestation | 0.051337567 | 2021 |
| DALYs cases | High SDI | Child underweight | 0.081087057 | 2021 |
| DALYs cases | High SDI | Vitamin A deficiency | 0.00222384 | 2021 |
| DALYs cases | High SDI | Zinc deficiency | 0.002926047 | 2021 |
| DALYs cases | High SDI | Unsafe sanitation | 0.087513782 | 2021 |
| DALYs cases | High SDI | Non-exclusive breastfeeding | 0.184111443 | 2021 |
| DALYs cases | High SDI | Discontinued breastfeeding | 0.058755707 | 2021 |
| DALYs cases | High-middle SDI | Ambient particulate matter pollution | 0.005649506 | 2021 |
| DALYs cases | High-middle SDI | Household air pollution from solid fuels | 0.000111873 | 2021 |
| DALYs cases | High-middle SDI | No access to handwashing facility | 0.028114342 | 2021 |
| DALYs cases | High-middle SDI | Unsafe water source | 0.344987504 | 2021 |
| DALYs cases | High-middle SDI | Child wasting | 0.158372613 | 2021 |
| DALYs cases | High-middle SDI | Child stunting | 0.066285844 | 2021 |
| DALYs cases | High-middle SDI | Short gestation for birth weight | 0.010726701 | 2021 |
| DALYs cases | High-middle SDI | Low birth weight for gestation | 0.031183247 | 2021 |
| DALYs cases | High-middle SDI | Child underweight | 0.088497349 | 2021 |
| DALYs cases | High-middle SDI | Vitamin A deficiency | 0.00394978 | 2021 |
| DALYs cases | High-middle SDI | Zinc deficiency | 0.000998366 | 2021 |
| DALYs cases | High-middle SDI | Unsafe sanitation | 0.143085009 | 2021 |
| DALYs cases | High-middle SDI | Non-exclusive breastfeeding | 0.097073164 | 2021 |
| DALYs cases | High-middle SDI | Discontinued breastfeeding | 0.0209647 | 2021 |
| DALYs cases | Low-middle SDI | Ambient particulate matter pollution | 0.002359678 | 2021 |
| DALYs cases | Low-middle SDI | Household air pollution from solid fuels | 0.00527964 | 2021 |
| DALYs cases | Low-middle SDI | No access to handwashing facility | 0.073147658 | 2021 |
| DALYs cases | Low-middle SDI | Unsafe water source | 0.276262866 | 2021 |
| DALYs cases | Low-middle SDI | Child wasting | 0.152543244 | 2021 |
| DALYs cases | Low-middle SDI | Child stunting | 0.073587305 | 2021 |
| DALYs cases | Low-middle SDI | Short gestation for birth weight | 0.010000094 | 2021 |
| DALYs cases | Low-middle SDI | Low birth weight for gestation | 0.02041177 | 2021 |
| DALYs cases | Low-middle SDI | Child underweight | 0.108919448 | 2021 |
| DALYs cases | Low-middle SDI | Vitamin A deficiency | 0.008445092 | 2021 |
| DALYs cases | Low-middle SDI | Zinc deficiency | 0.001788221 | 2021 |
| DALYs cases | Low-middle SDI | Unsafe sanitation | 0.203496688 | 2021 |
| DALYs cases | Low-middle SDI | Non-exclusive breastfeeding | 0.055210873 | 2021 |
| DALYs cases | Low-middle SDI | Discontinued breastfeeding | 0.008547422 | 2021 |
| DALYs cases | Low SDI | Ambient particulate matter pollution | 0.000844479 | 2021 |
| DALYs cases | Low SDI | Household air pollution from solid fuels | 0.004508779 | 2021 |
| DALYs cases | Low SDI | No access to handwashing facility | 0.093384008 | 2021 |
| DALYs cases | Low SDI | Unsafe water source | 0.250961499 | 2021 |
| DALYs cases | Low SDI | Child wasting | 0.159004696 | 2021 |
| DALYs cases | Low SDI | Child stunting | 0.081768537 | 2021 |
| DALYs cases | Low SDI | Short gestation for birth weight | 0.005894485 | 2021 |
| DALYs cases | Low SDI | Low birth weight for gestation | 0.012463056 | 2021 |
| DALYs cases | Low SDI | Child underweight | 0.118404457 | 2021 |
| DALYs cases | Low SDI | Vitamin A deficiency | 0.010986463 | 2021 |
| DALYs cases | Low SDI | Zinc deficiency | 0.001392973 | 2021 |
| DALYs cases | Low SDI | Unsafe sanitation | 0.20919489 | 2021 |
| DALYs cases | Low SDI | Non-exclusive breastfeeding | 0.044862843 | 2021 |
| DALYs cases | Low SDI | Discontinued breastfeeding | 0.006328835 | 2021 |
| DALYs cases | Middle SDI | Ambient particulate matter pollution | 0.00345626 | 2021 |
| DALYs cases | Middle SDI | Household air pollution from solid fuels | 0.001680162 | 2021 |
| DALYs cases | Middle SDI | No access to handwashing facility | 0.056706571 | 2021 |
| DALYs cases | Middle SDI | Unsafe water source | 0.296266104 | 2021 |
| DALYs cases | Middle SDI | Child wasting | 0.17236669 | 2021 |
| DALYs cases | Middle SDI | Child stunting | 0.076719118 | 2021 |
| DALYs cases | Middle SDI | Short gestation for birth weight | 0.007278547 | 2021 |
| DALYs cases | Middle SDI | Low birth weight for gestation | 0.018266801 | 2021 |
| DALYs cases | Middle SDI | Child underweight | 0.108378957 | 2021 |
| DALYs cases | Middle SDI | Vitamin A deficiency | 0.006540581 | 2021 |
| DALYs cases | Middle SDI | Zinc deficiency | 0.00116257 | 2021 |
| DALYs cases | Middle SDI | Unsafe sanitation | 0.163348263 | 2021 |
| DALYs cases | Middle SDI | Non-exclusive breastfeeding | 0.072294487 | 2021 |
| DALYs cases | Middle SDI | Discontinued breastfeeding | 0.01553489 | 2021 |
| DALYs rate | Global | Ambient particulate matter pollution | 0.001324079 | 1990 |
| DALYs rate | Global | Household air pollution from solid fuels | 0.006807494 | 1990 |
| DALYs rate | Global | No access to handwashing facility | 0.072940955 | 1990 |
| DALYs rate | Global | Unsafe water source | 0.230738543 | 1990 |
| DALYs rate | Global | Child wasting | 0.158870022 | 1990 |
| DALYs rate | Global | Child stunting | 0.091839474 | 1990 |
| DALYs rate | Global | Short gestation for birth weight | 0.010201649 | 1990 |
| DALYs rate | Global | Low birth weight for gestation | 0.020902384 | 1990 |
| DALYs rate | Global | Child underweight | 0.125997 | 1990 |
| DALYs rate | Global | Vitamin A deficiency | 0.018088258 | 1990 |
| DALYs rate | Global | Zinc deficiency | 0.003087874 | 1990 |
| DALYs rate | Global | Unsafe sanitation | 0.197186443 | 1990 |
| DALYs rate | Global | Non-exclusive breastfeeding | 0.054311198 | 1990 |
| DALYs rate | Global | Discontinued breastfeeding | 0.007704629 | 1990 |
| DALYs rate | High SDI | Ambient particulate matter pollution | 0.003998689 | 1990 |
| DALYs rate | High SDI | Household air pollution from solid fuels | 0.00031037 | 1990 |
| DALYs rate | High SDI | No access to handwashing facility | 0.035775724 | 1990 |
| DALYs rate | High SDI | Unsafe water source | 0.254073596 | 1990 |
| DALYs rate | High SDI | Child wasting | 0.186662365 | 1990 |
| DALYs rate | High SDI | Child stunting | 0.075299882 | 1990 |
| DALYs rate | High SDI | Short gestation for birth weight | 0.001647693 | 1990 |
| DALYs rate | High SDI | Low birth weight for gestation | 0.01715687 | 1990 |
| DALYs rate | High SDI | Child underweight | 0.101342867 | 1990 |
| DALYs rate | High SDI | Vitamin A deficiency | 0.004519527 | 1990 |
| DALYs rate | High SDI | Zinc deficiency | 0.001946961 | 1990 |
| DALYs rate | High SDI | Unsafe sanitation | 0.178927622 | 1990 |
| DALYs rate | High SDI | Non-exclusive breastfeeding | 0.109142617 | 1990 |
| DALYs rate | High SDI | Discontinued breastfeeding | 0.029195217 | 1990 |
| DALYs rate | High-middle SDI | Ambient particulate matter pollution | 0.002540767 | 1990 |
| DALYs rate | High-middle SDI | Household air pollution from solid fuels | 0.001999741 | 1990 |
| DALYs rate | High-middle SDI | No access to handwashing facility | 0.038909278 | 1990 |
| DALYs rate | High-middle SDI | Unsafe water source | 0.264049171 | 1990 |
| DALYs rate | High-middle SDI | Child wasting | 0.168128373 | 1990 |
| DALYs rate | High-middle SDI | Child stunting | 0.081230988 | 1990 |
| DALYs rate | High-middle SDI | Short gestation for birth weight | 0.003376259 | 1990 |
| DALYs rate | High-middle SDI | Low birth weight for gestation | 0.015477029 | 1990 |
| DALYs rate | High-middle SDI | Child underweight | 0.097707162 | 1990 |
| DALYs rate | High-middle SDI | Vitamin A deficiency | 0.011546582 | 1990 |
| DALYs rate | High-middle SDI | Zinc deficiency | 0.000912683 | 1990 |
| DALYs rate | High-middle SDI | Unsafe sanitation | 0.223246253 | 1990 |
| DALYs rate | High-middle SDI | Non-exclusive breastfeeding | 0.067922903 | 1990 |
| DALYs rate | High-middle SDI | Discontinued breastfeeding | 0.022952812 | 1990 |
| DALYs rate | Low-middle SDI | Ambient particulate matter pollution | 0.001487461 | 1990 |
| DALYs rate | Low-middle SDI | Household air pollution from solid fuels | 0.008367646 | 1990 |
| DALYs rate | Low-middle SDI | No access to handwashing facility | 0.071151964 | 1990 |
| DALYs rate | Low-middle SDI | Unsafe water source | 0.227677648 | 1990 |
| DALYs rate | Low-middle SDI | Child wasting | 0.155686255 | 1990 |
| DALYs rate | Low-middle SDI | Child stunting | 0.090689289 | 1990 |
| DALYs rate | Low-middle SDI | Short gestation for birth weight | 0.013367421 | 1990 |
| DALYs rate | Low-middle SDI | Low birth weight for gestation | 0.025591772 | 1990 |
| DALYs rate | Low-middle SDI | Child underweight | 0.127762162 | 1990 |
| DALYs rate | Low-middle SDI | Vitamin A deficiency | 0.016969693 | 1990 |
| DALYs rate | Low-middle SDI | Zinc deficiency | 0.003559645 | 1990 |
| DALYs rate | Low-middle SDI | Unsafe sanitation | 0.1958312 | 1990 |
| DALYs rate | Low-middle SDI | Non-exclusive breastfeeding | 0.055461909 | 1990 |
| DALYs rate | Low-middle SDI | Discontinued breastfeeding | 0.006395936 | 1990 |
| DALYs rate | Low SDI | Ambient particulate matter pollution | 0.000873181 | 1990 |
| DALYs rate | Low SDI | Household air pollution from solid fuels | 0.0058286 | 1990 |
| DALYs rate | Low SDI | No access to handwashing facility | 0.086338314 | 1990 |
| DALYs rate | Low SDI | Unsafe water source | 0.229231694 | 1990 |
| DALYs rate | Low SDI | Child wasting | 0.15868405 | 1990 |
| DALYs rate | Low SDI | Child stunting | 0.093750012 | 1990 |
| DALYs rate | Low SDI | Short gestation for birth weight | 0.008353114 | 1990 |
| DALYs rate | Low SDI | Low birth weight for gestation | 0.016163559 | 1990 |
| DALYs rate | Low SDI | Child underweight | 0.128773411 | 1990 |
| DALYs rate | Low SDI | Vitamin A deficiency | 0.021443968 | 1990 |
| DALYs rate | Low SDI | Zinc deficiency | 0.003036315 | 1990 |
| DALYs rate | Low SDI | Unsafe sanitation | 0.195084886 | 1990 |
| DALYs rate | Low SDI | Non-exclusive breastfeeding | 0.046541018 | 1990 |
| DALYs rate | Low SDI | Discontinued breastfeeding | 0.005897878 | 1990 |
| DALYs rate | Middle SDI | Ambient particulate matter pollution | 0.001740737 | 1990 |
| DALYs rate | Middle SDI | Household air pollution from solid fuels | 0.005003997 | 1990 |
| DALYs rate | Middle SDI | No access to handwashing facility | 0.051121181 | 1990 |
| DALYs rate | Middle SDI | Unsafe water source | 0.239604929 | 1990 |
| DALYs rate | Middle SDI | Child wasting | 0.167548762 | 1990 |
| DALYs rate | Middle SDI | Child stunting | 0.092050035 | 1990 |
| DALYs rate | Middle SDI | Short gestation for birth weight | 0.005897786 | 1990 |
| DALYs rate | Middle SDI | Low birth weight for gestation | 0.018593107 | 1990 |
| DALYs rate | Middle SDI | Child underweight | 0.117518745 | 1990 |
| DALYs rate | Middle SDI | Vitamin A deficiency | 0.014417113 | 1990 |
| DALYs rate | Middle SDI | Zinc deficiency | 0.002048568 | 1990 |
| DALYs rate | Middle SDI | Unsafe sanitation | 0.203408618 | 1990 |
| DALYs rate | Middle SDI | Non-exclusive breastfeeding | 0.067045083 | 1990 |
| DALYs rate | Middle SDI | Discontinued breastfeeding | 0.014001336 | 1990 |
| DALYs rate | Global | Ambient particulate matter pollution | 0.001406152 | 2021 |
| DALYs rate | Global | Household air pollution from solid fuels | 0.004499106 | 2021 |
| DALYs rate | Global | No access to handwashing facility | 0.085764758 | 2021 |
| DALYs rate | Global | Unsafe water source | 0.260453783 | 2021 |
| DALYs rate | Global | Child wasting | 0.158270975 | 2021 |
| DALYs rate | Global | Child stunting | 0.079348541 | 2021 |
| DALYs rate | Global | Short gestation for birth weight | 0.007017758 | 2021 |
| DALYs rate | Global | Low birth weight for gestation | 0.01489189 | 2021 |
| DALYs rate | Global | Child underweight | 0.115278736 | 2021 |
| DALYs rate | Global | Vitamin A deficiency | 0.010044106 | 2021 |
| DALYs rate | Global | Zinc deficiency | 0.001476172 | 2021 |
| DALYs rate | Global | Unsafe sanitation | 0.204525498 | 2021 |
| DALYs rate | Global | Non-exclusive breastfeeding | 0.049463868 | 2021 |
| DALYs rate | Global | Discontinued breastfeeding | 0.007558656 | 2021 |
| DALYs rate | High SDI | Ambient particulate matter pollution | 0.006234526 | 2021 |
| DALYs rate | High SDI | Household air pollution from solid fuels | 2.73E-05 | 2021 |
| DALYs rate | High SDI | No access to handwashing facility | 0.034174067 | 2021 |
| DALYs rate | High SDI | Unsafe water source | 0.261367806 | 2021 |
| DALYs rate | High SDI | Child wasting | 0.167814218 | 2021 |
| DALYs rate | High SDI | Child stunting | 0.051716144 | 2021 |
| DALYs rate | High SDI | Short gestation for birth weight | 0.010710543 | 2021 |
| DALYs rate | High SDI | Low birth weight for gestation | 0.051337567 | 2021 |
| DALYs rate | High SDI | Child underweight | 0.081087057 | 2021 |
| DALYs rate | High SDI | Vitamin A deficiency | 0.00222384 | 2021 |
| DALYs rate | High SDI | Zinc deficiency | 0.002926047 | 2021 |
| DALYs rate | High SDI | Unsafe sanitation | 0.087513782 | 2021 |
| DALYs rate | High SDI | Non-exclusive breastfeeding | 0.184111443 | 2021 |
| DALYs rate | High SDI | Discontinued breastfeeding | 0.058755707 | 2021 |
| DALYs rate | High-middle SDI | Ambient particulate matter pollution | 0.005649506 | 2021 |
| DALYs rate | High-middle SDI | Household air pollution from solid fuels | 0.000111873 | 2021 |
| DALYs rate | High-middle SDI | No access to handwashing facility | 0.028114342 | 2021 |
| DALYs rate | High-middle SDI | Unsafe water source | 0.344987504 | 2021 |
| DALYs rate | High-middle SDI | Child wasting | 0.158372613 | 2021 |
| DALYs rate | High-middle SDI | Child stunting | 0.066285844 | 2021 |
| DALYs rate | High-middle SDI | Short gestation for birth weight | 0.010726701 | 2021 |
| DALYs rate | High-middle SDI | Low birth weight for gestation | 0.031183247 | 2021 |
| DALYs rate | High-middle SDI | Child underweight | 0.088497349 | 2021 |
| DALYs rate | High-middle SDI | Vitamin A deficiency | 0.00394978 | 2021 |
| DALYs rate | High-middle SDI | Zinc deficiency | 0.000998366 | 2021 |
| DALYs rate | High-middle SDI | Unsafe sanitation | 0.143085009 | 2021 |
| DALYs rate | High-middle SDI | Non-exclusive breastfeeding | 0.097073164 | 2021 |
| DALYs rate | High-middle SDI | Discontinued breastfeeding | 0.0209647 | 2021 |
| DALYs rate | Low-middle SDI | Ambient particulate matter pollution | 0.002359678 | 2021 |
| DALYs rate | Low-middle SDI | Household air pollution from solid fuels | 0.00527964 | 2021 |
| DALYs rate | Low-middle SDI | No access to handwashing facility | 0.073147658 | 2021 |
| DALYs rate | Low-middle SDI | Unsafe water source | 0.276262866 | 2021 |
| DALYs rate | Low-middle SDI | Child wasting | 0.152543244 | 2021 |
| DALYs rate | Low-middle SDI | Child stunting | 0.073587305 | 2021 |
| DALYs rate | Low-middle SDI | Short gestation for birth weight | 0.010000094 | 2021 |
| DALYs rate | Low-middle SDI | Low birth weight for gestation | 0.02041177 | 2021 |
| DALYs rate | Low-middle SDI | Child underweight | 0.108919448 | 2021 |
| DALYs rate | Low-middle SDI | Vitamin A deficiency | 0.008445092 | 2021 |
| DALYs rate | Low-middle SDI | Zinc deficiency | 0.001788221 | 2021 |
| DALYs rate | Low-middle SDI | Unsafe sanitation | 0.203496688 | 2021 |
| DALYs rate | Low-middle SDI | Non-exclusive breastfeeding | 0.055210873 | 2021 |
| DALYs rate | Low-middle SDI | Discontinued breastfeeding | 0.008547422 | 2021 |
| DALYs rate | Low SDI | Ambient particulate matter pollution | 0.000844479 | 2021 |
| DALYs rate | Low SDI | Household air pollution from solid fuels | 0.004508779 | 2021 |
| DALYs rate | Low SDI | No access to handwashing facility | 0.093384008 | 2021 |
| DALYs rate | Low SDI | Unsafe water source | 0.250961499 | 2021 |
| DALYs rate | Low SDI | Child wasting | 0.159004696 | 2021 |
| DALYs rate | Low SDI | Child stunting | 0.081768537 | 2021 |
| DALYs rate | Low SDI | Short gestation for birth weight | 0.005894485 | 2021 |
| DALYs rate | Low SDI | Low birth weight for gestation | 0.012463056 | 2021 |
| DALYs rate | Low SDI | Child underweight | 0.118404457 | 2021 |
| DALYs rate | Low SDI | Vitamin A deficiency | 0.010986463 | 2021 |
| DALYs rate | Low SDI | Zinc deficiency | 0.001392973 | 2021 |
| DALYs rate | Low SDI | Unsafe sanitation | 0.20919489 | 2021 |
| DALYs rate | Low SDI | Non-exclusive breastfeeding | 0.044862843 | 2021 |
| DALYs rate | Low SDI | Discontinued breastfeeding | 0.006328835 | 2021 |
| DALYs rate | Middle SDI | Ambient particulate matter pollution | 0.00345626 | 2021 |
| DALYs rate | Middle SDI | Household air pollution from solid fuels | 0.001680162 | 2021 |
| DALYs rate | Middle SDI | No access to handwashing facility | 0.056706571 | 2021 |
| DALYs rate | Middle SDI | Unsafe water source | 0.296266104 | 2021 |
| DALYs rate | Middle SDI | Child wasting | 0.17236669 | 2021 |
| DALYs rate | Middle SDI | Child stunting | 0.076719118 | 2021 |
| DALYs rate | Middle SDI | Short gestation for birth weight | 0.007278547 | 2021 |
| DALYs rate | Middle SDI | Low birth weight for gestation | 0.018266801 | 2021 |
| DALYs rate | Middle SDI | Child underweight | 0.108378957 | 2021 |
| DALYs rate | Middle SDI | Vitamin A deficiency | 0.006540581 | 2021 |
| DALYs rate | Middle SDI | Zinc deficiency | 0.00116257 | 2021 |
| DALYs rate | Middle SDI | Unsafe sanitation | 0.163348263 | 2021 |
| DALYs rate | Middle SDI | Non-exclusive breastfeeding | 0.072294487 | 2021 |
| DALYs rate | Middle SDI | Discontinued breastfeeding | 0.01553489 | 2021 |
